# Genetic structure and demographic history of *Lymantria dispar* (Linnaeus, 1758) (Lepidoptera: Erebidae) in its area of origin and adjacent areas

**DOI:** 10.1002/ece3.3467

**Published:** 2017-09-30

**Authors:** Tae Hwa Kang, Sang Hoon Han, Heung Sik Lee

**Affiliations:** ^1^ Bio Control Research Center Jeonnam Bioindustry Foundation Gokseong‐gun Korea; ^2^ Department of Life Science College of Natural Science Kyonggi University Suwon Korea; ^3^ Plant Quarantine Technology Center Animal and Plant Quarantine Agency Gimcheon‐si Korea

**Keywords:** demographic history, Far East Asia, *Lymantria dispar*, population genetic structure, species origin region

## Abstract

We analyzed the population genetic structure and demographic history of 20 *Lymantria dispar* populations from Far East Asia using microsatellite loci and mitochondrial genes. In the microsatellite analysis, the genetic distances based on pairwise *F*_ST_ values ranged from 0.0087 to 0.1171. A NeighborNet network based on pairwise *F*_ST_ genetic distances showed that the 20 regional populations were divided into five groups. Bayesian clustering analysis (*K* = 3) demonstrated the same groupings. The populations in the Korean Peninsula and adjacent regions, in particular, showed a mixed genetic pattern. In the mitochondrial genetic analysis based on 98 haplotypes, the median‐joining network exhibited a star shape that was focused on three high‐frequency haplotypes (Haplotype 1: central Korea and adjacent regions, Group 1; Haplotype 37: southern Korea, Group 2; and Haplotype 90: Hokkaido area, Group 3) connected by low‐frequency haplotypes. The mismatch distribution dividing the three groups was unimodal. In the neutral test, Tajima's D and Fu's FS tests were negative. We can thus infer that the Far East Asian populations of *L. dispar* underwent a sudden population expansion. Based on the age expansion parameter, the expansion time was inferred to be approximately 53,652 years before present (ybp) for Group 1, approximately 65,043 ybp for Group 2, and approximately 76,086 ybp for Group 3. We propose that the mixed genetic pattern of the inland populations of Far East Asia is due to these expansions and that the inland populations of the region should be treated as valid subspecies that are distinguishable from other subspecies by genetic traits.

## 1 INTRODUCTION

**Figure 1:**
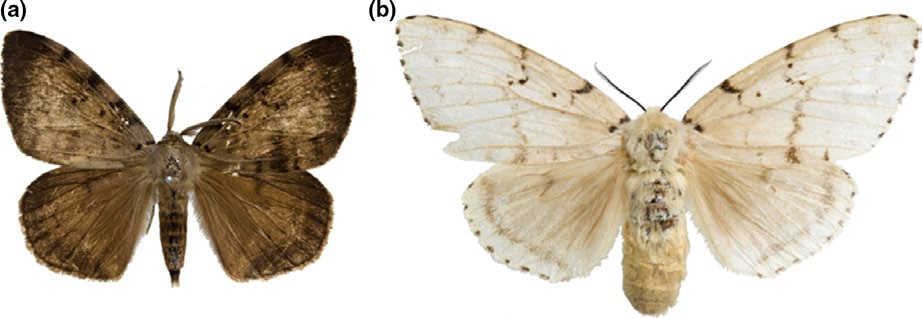
Adult habitus of *Lymantria dispar asiatica* (a, male; b, female)

The gypsy moth, *Lymantria dispar* (Linnaeus, 1758), originating from Hokkaido, Japan (Bogdanowicz, Mastro, Prasher, & Harrison, 1997; Bogdanowicz, Schaefer, & Harrison, 2000; Goldschmidt, 1934, 1940), is widely distributed in the Palearctic region (Pogue & Schaefer, 2007; Schintlmeister, 2004). There are three subspecies: *L. dispar dispar*,* L. dispar asiatica* Vnukovskij, 1926, and *L. dispar japonica* Motschulsky, 1860 (Pogue & Schaefer, 2007). *Lymantria dispar dispar* is mainly distributed in Europe, *L. dispar asiatica* occurs from Central Asia to East Asia, and *L. dispar japonica* is present only in Japan (Pogue & Schaefer, 2007). Among these subspecies, the validity of the scientific name *L. dispar asiatica* (Figure 1) has been debated by many authors (Lee, Kang, Jeong, Ryu, & Lee, 2015). Schintlmeister treated *L. dispar asiatica* as a synonym of *L. dispar dispar* on the basis of their type locality; however, Pogue and Schaefer treated the subspecies as valid based on the morphological characteristics of the females, which have larger wings than the females of *L. dispar dispar* (Lee et al., 2015; Pogue & Schaefer, 2007; Schintlmeister, 2004). The dispersal ability of the two subspecies may differ because of these differences in wing size. Based on research of male deaths after interbreeding, Higashiura et al. (2011) accepted the five subspecies of Inoue (1982). Thus, the subspecies of *L. dispar* are clearly in a state of confusion.

*Lymantria dispar dispar* was intentionally brought to North America for hybridization experiments; however, some individuals escaped in either 1868 or 1869 (Liebhold, Mastro, & Schaefer, 1989). Since then, the subspecies has become an invasive forest pest, causing injury to approximately 400 species of plants (Lowe, Browne, Boudjelas, & De Poorter, 2000; Pogue & Schaefer, 2007). Approximately US$11 million is spent on European gypsy moth control every year (Pimentel, Zuniga, & Morrison, 2005; Pogue & Schaefer, 2007). For these reasons, *L. dispar asiatica,* which has higher flight capability than *L. dispar dispar,* has been treated as a quarantine pest in North America (Pogue & Schaefer, 2007).

Due to the quarantine and danger this invasive species represents, studies of the differences among local populations are actively conducted. In previous decades, population genetic analyses of *L. dispar* were performed using various methods, such as allozyme detection, amplified fragment length polymorphism, restriction fragment length polymorphism, sequence‐based analysis, and microsatellites (Bogdanowicz et al., 1997, 2000; deWaard et al., 2010; George, 1984; Kang, Lee, & Lee, 2015; Keena, Cȏté, Grinberg, & Wallner, 2008; Koshio, Tomishima, Shimizu, Kim, & Takenaka, 2002; Qian et al., 2014; Wu et al., 2015). Area of origin studies, in particular, using microsatellite loci were mainly conducted by North American researchers. In the first attempt by Bogdanowicz et al. (1997), four markers were developed and used to assay allelic variation in four gypsy moth populations (Japan, Far East Russia, China, and North America). Subsequently, Keena et al. (2008) evaluated flight capability and related traits using four microsatellite loci (from Bogdanowicz et al., 1997) and mitochondrial DNA analyses of samples obtained from 46 geographic strains. In Far East Asia, Koshio et al. (2002) compared the allele types of regional populations using three microsatellite loci of Japanese samples from three local populations; however, they did not consider population structure because of small sample sizes. Recently, Wu et al. (2015) thoroughly analyzed the population structure of the Holarctic gypsy moth and performed an origin test for each regional population using nine microsatellite loci, including three from Bogdanowicz et al. (1997).

These studies were conducted from the perspective of quarantine inspection (or invasive species control), and the number of sampled individuals was large; however, the number of sampled areas in each region was small, leading to taxonomic confusion with respect to the subspecies of *L. dispar*. For example, it was reported that two Asian subspecies, *L. dispar asiatica* and *L. dispar japonica,* were difficult to distinguish using morphological characters, with individuals of *L. dispar asiatica* collected from the southern coastal area of Korea having characteristics similar to *L. dispar japonica* (Lee et al., 2015; Pogue & Schaefer, 2007). To resolve this taxonomic confusion at the subspecific level, a demographic history of the Far East Asian populations of *L. dispar* based on intensive sampling is required. Therefore, the goal of this study was to reveal the population genetic structure and demographic history of *L. dispar* in Far East Asia, including in the region of species origin: Hokkaido, Japan. For this purpose, we analyzed the genetic diversity and demographic history of *L. dispar* from Far East Asia using eight microsatellite loci and three mitochondrial genes (cytochrome c oxidase I [COI], ATP6, and ATP8 genes).

Genetic diversity analyses using microsatellite loci have been conducted for various eukaryotes (Balloux & Lugon‐Moulin, 2002; Sakai et al., 2001; Sunnucks, 2000). Recently, they have been used to track the influx of invasive species (Hess, Swalla, & Moran, 2008; Hunter & Hart, 2013; Keena et al., 2008; Kim et al., 2011; King, Eackles, & Chapman, 2011; Tóth, Gáspári, & Jurka, 2000). For the use of microsatellite loci, however, a primer set for each polymorphic locus is required. The general method employed is an enrichment strategy (López‐Uribe, Santiago, Bogdanowicz, & Danforth, 2012; Richardson, Stanley, & Sherman, 2012), which is expensive and time‐consuming, as it is based on traditional cloning strategies (Perry & Rowe, 2011; Santana et al., 2009; Zane, Bargelloni, & Patarnello, 2002). However, the next‐generation sequencing (NGS) technique is very useful for the construction of microsatellite loci libraries at a lower cost and far more quickly than traditional cloning‐based approaches (Hess et al., 2008; Kang, Han, & Park, 2016; Kang, Han, & Park, 2015; Perry & Rowe, 2011; Yu, Won, Jun, Lim, & Kwak, 2011). Because of the problems associated with the traditional cloning strategies, we used Illumina sequencing, one of the NGS techniques, for reading the genomic DNA of *L. dispar* and then developed microsatellite markers from the results.

## 2 MATERIALS AND METHODS

### 2.1 Sampling and genomic DNA extraction for NGS and pyrosequencing

**Figure 2:**
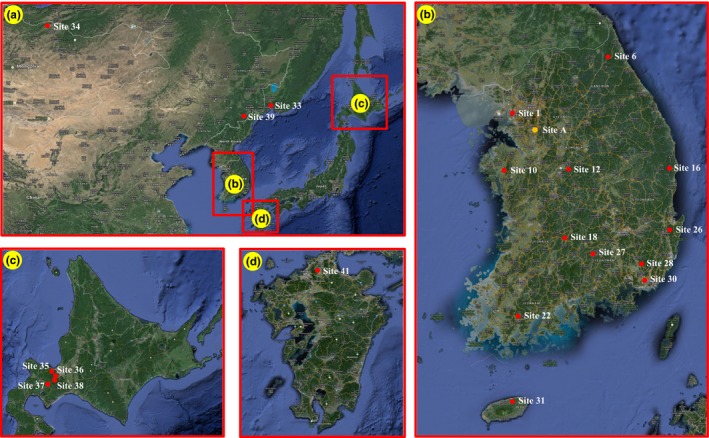
Collection sites of *Lymantria dispar* in Far East Asia

For NGS, we extracted genomic DNA from an egg mass of *L. dispar*. The egg mass was collected from Suwon, Korea (37°14.092′N, 127°02.840′E; Figure 2b: Site A). In the egg mass, we selected 50 eggs and extracted genomic DNA using a NucleoSpin^®^ Tissue Kit (Macherey‐Nagel GmbH, Düren, Germany) following the manufacturer's instructions. The sequencing was performed with a MiSeq Sequencer (Illumina, San Diego, CA, USA) by the DNA sequencing company DisGene (Daejeon, Korea). The resulting contigs were assembled in CLC workbench (CLC Bio, Aarhus, Denmark).

### 2.2 Sampling and genomic DNA extraction for genetic structure analysis

For polymerase chain reaction (PCR) analysis of polymorphisms with the developed microsatellite markers and for genetic structure analysis, 552 samples were collected from 20 sites in Mongolia (1), Russia (1), China (1), Korea (12), and Japan (5) using pheromone attraction traps (Figure 2, Table 1). The thoracic muscle of each individual was removed for the extraction of genomic DNA. For morphological examination, fore and hind wings were prepared as specimens on a glue board. Abdomens were maintained at −20°C for examination of genitalia. Genomic DNA was extracted using a DNeasy^®^ Blood & Tissue Kit (Qiagen, Leipzig, Germany) according to the manufacturer's instructions.

**Table 1:** Collection sites of *Lymantria dispar* in Far East Asia

Sn	CL	GPS	CIn	Sn	SSn	COI GAn	ATP6/ATP8 GAn	GSn
A	Korea, GG, Suwon‐si, Yeongtong‐gu, Mangpo‐dong	37°14.092′N 127°02.840′E	Egg mass	For NGS	–	–	–	–
1	Incheon, Gyeyang‐gu, Gyesan‐dong	37°32′57.9″N 126°43′42.7″E	30	192–221	30	KT245170–KT245199	KX945522–KX945551	20
6	GW, Inje‐gun, Buk‐myeon, Hangye‐ri	38°08′09.5″N 128°15′40.1″E	30	312–341	28	KT245288–KT245317	KX945552–KX945579	20
10	CN, Seosan‐si, Haemi‐myeon, Daegok‐ri	36°41′55.4″N 126°35′35.1″E	30	432–461	26	KT245405–KT245430	KX945580–KX945605	20
12	CB, Cheongweon‐gun, Miweon‐myeon, Daesin‐ri	36°41′46.2″N 127°36′27.3″E	30	492–521	23	KT245496–KT245480	KX945606–KX945628	20
16	GB, Yeongyang‐gun, Subi‐myeon, Suha‐ri	36°50′23.4″N 129°16′22.5″E	30	612–641	17	KT245558–KT245584	KX945629–KX945645	20
18	JB, Jinan‐gun, Jinan‐eup, Danyang‐ri	35°45′55.8″N 127°25′00.6″E	30	672–701	28	KT245609–KT245636	KX945646–KX945673	20
22	JN, Gangjin‐gun, Jakcheon‐myeon, Galdong‐ri	34°43′00.3″N 126°43′49.5″E	30	792–821	29	KT245722–KT245750	KX945674–KX945702	20
26	GB, Gyeongju‐si, Yonggang‐dong	35°51′45.4″N 129°14′14.7″E	30	912–941	30	KT245840–KT245869	KX945703–KX945732	20
27	GN, Hapcheon‐gun, Daebyeong‐myeon, Hageum‐ri	35°31′27.9″N 127°59′12.1″E	30	942–971	22	KT245870–KT245899	KX945733–KX945754	20
28	GN, Milyang‐si, Bubuk‐myeon, Jeonsapo‐ri	35°27′30.5″N 128°44′11.6″E	30	972–1,001	28	KT245900–KT245929	KX945755–KX945782	20
30	GN, Gimhae‐si, Saman‐dong	35°15′16.0″N 128°54′51.3″E	30	1,032–1,061	30	KT245960–KT245989	KX945783–KX945812	20
31	JJ, Jeju‐si, Bonggae‐dong	33°26′15.0″N 126°37′43.8″E	30	1,062–1,091	30	KT245990–KT246019	KX945813–KX945842	20
33	Russia, Vladivostok	43°23′44.6″N 132°09′56.6″E	30	1,703–1,732	28	KT246046–KT246075	KX945843–KX945870	30
34	Mongolia Selenge Province Shaganuur	50°15′N105°30′E	30	1,733–1,749	13	KX945391–KX945403	KX945871–KX945883	17
35	Japan Hokkaido Otaru Asarigawa‐onsen, 1 Chome	43°8.056′N141°2.395′E	30	1,870–1,899	23	KX945404–KX945426	KX945884–KX945906	30
36	Japan Hokkaido Sapporo Minami‐ku Jozankei Jozankei Lakeline	43°0.296′N)141°8.88′E	30	1,900–1,929	23	KX945427–KX945449	KX945907–KX945929	30
37	Japan Hokkaido Abuta Kimobetsu‐cho Fushimi	42°48.098′N 140°58.172′E	30	1,930–1,959	23	KX945450–KX945472	KX945930–KX945952	30
38	Japan Hokkaido Sapporo Minami‐ku Jozankei‐onsen higashi 4 Chome	42°57.666′N 141°9.431′E	30	1,960–1,989	26	KX945473–KX945498	KX945953–KX945978	30
39	China Jilin Helong Qingshanli	42°26′22.6″N 128°51′50.3″E	18	1,990–2,007	17	KX945499–KX945515	KX945979–KX945995	18
41	Japan Kyushu Fukuoka Miyawaka Mt. Inunaki	33°40′54.0″N 130°33′15.4″E	7	2,013–2,019	6	KX945516–KX945521	KX945996–KX946001	7
Total	20 sites	–	565	–	480	–	–	432

Sn, site number; CL, collecting location; CIn, number of collected individuals; Sn, sample number; SSn, number of sequenced samples; GAn, GenBank accession number; GSn, number of genotyped samples.

### 2.3 Microsatellite locus identification and marker development

Microsatellite loci were identified using Phobos ver. 3.3.12 (Leese, Mayer, & Held, 2008; Mayer, Leese, & Tollrian, 2010) with the following conditions: repeated sequence length, 2–4 base pairs (bp) and repeat count, greater than four. AT‐rich loci were excluded from the investigated microsatellite loci, and for loci that were repeated more than six times, primer sets were chosen using the primer design software PRIMER 3 (Koressaar & Remm, 2007; Untergrasser et al., 2012) with the following criteria: melting temperature, 55.5–56.5°C; GC content, over 30%; and primer length, 18–22 bp. A hundred and fifty primer sets were designed, and PCR tested for specificity and the presence of polymorphic amplification using one sample from each of the twelve regional populations from Korea. PCRs for the primer qualification test were conducted with AccuPower PCR PreMix (Bioneer, Daejeon, Korea) in a final volume of 20 μl containing 30 ng of template DNA and 5 pmol of each primer. Extra MgCl_2_ was not added. The amplification profile was 5 min at 94°C; 30 cycles of 10 s at 94°C, 10 s at 56°C, and 20 s at 72°C; and a final 5 min extension at 72°C. The specificity and presence of polymorphic amplification for each primer set were checked using a QIAxcel DNA high‐resolution cartridge (Qiagen, Leipzig, Germany). For the markers showing polymorphism in the electrophoresis, each forward (sense) primer for genotyping was labeled with 6‐carboxyfluorescein at the 5′ end (Schuelke, 2000). Of the labeled markers, eight were selected for microsatellite marker assessment by a PCR amplification test. For microsatellite marker assessment, 432 samples from the 20 regional populations were genotyped (Table 1). These PCRs were performed by the DNA sequencing company Bionics (Seoul, Korea).

### 2.4 Mitochondrial DNA sequencing

For the analysis of *L. dispar* genealogy in Far East Asia, we selected three mitochondrial genes: COI, ATP6, and ATP8. The COI gene may not be suitable for population analysis because its intraspecific variation is relatively low and its interspecific variation is relatively high (Cameron & Whiting, 2008; Wu et al., 2015); however, when combined with other genes, it may be useful (Hajibabaei, Singer, Hebert, & Hickey, 2007). The ATP6 and ATP8 genes show relatively higher intraspecific variation and are known to be suitable for population genetic analysis (Cameron & Whiting, 2008; Wu et al., 2015). The former region of the COI gene was amplified using the LCO1490 (5′‐GGTCAACAAATCATAAAGATATTGG‐3′) and HCO2198 (5′TAAACTTCAGGGTGACCAA AAAATCA‐3′) primer set (Folmer, Black, Hoeh, Lutz, & Vrijenhock, 1994) and a GeneMax Tc‐s‐B PCR cycler (BIOER, Hangzhou, China). PCR conditions were set as in Hebert, Cywinska, Ball, and deWaard (2003). The ATP6 and ATP8 genes were amplified using the primer set from Wu et al. (2015) and an ABI Veriti 96‐well Thermal Cycler (Applied Biosystems^®^; Thermo Fisher Scientific Inc., MA, USA). PCR products were checked using 1% agarose gel electrophoresis. The PCR products were purified and sequenced using the sequencing services of Macrogen (Seoul, Korea) and Bionics (Seoul, Korea). The obtained sequences were submitted to NCBI GenBank (Table 1).

### 2.5 Microsatellite loci data analysis

Genotyping errors (such as null alleles and scoring errors) on selected markers were checked with MICRO‐CHECKER ver. 2.2.3 (Oosterhout, Hutchinson, Wills, & Shipley, 2004). The pairwise linkage disequilibrium values for pairs of loci were then examined using Arlequin ver. 3.1 (Excoffier, Laval, & Schneider, 2005). Genetic diversity parameters such as allele frequency, genotype number, allele type, gene diversity, heterozygosity, and polymorphism information content (PIC) were calculated with PowerMarker ver. 3.5 (Liu & Muse, 2005). Hardy–Weinberg equilibrium (HWE) across loci was estimated after sequential Bonferroni correction (Rice, 1989). To test the isolation by distance (IBD) model, the correlation between genetic distance and geographic distance was calculated using Mantel's test with 30,000 randomizations in IBD ver. 3.23 (Jensen, Bohonak, & Kelley, 2005). To estimate genetic differentiation among regional populations, analysis of molecular variance (AMOVA) was used. AMOVA was calculated using the Kimura two‐parameter model in Arlequin ver. 3.1 (Excoffier et al., 2005). We ascertained the allele type frequencies based on microsatellite loci for each population and estimated the pairwise genetic distances between the populations based on allele type frequencies with PowerMarker ver. 3.5 (Liu & Muse, 2005). Based on the pairwise genetic distances, a network estimating the genealogical relations among the 20 regional populations was calculated with SplitTree4 (Huson & Bryant, 2006). We tested the genetic differentiation among the populations using a model‐based Bayesian analysis with STRUCTURE ver. 2.3.4 (Falush, Stephens, & Pritchard, 2003; Pritchard, Stephens, & Donnelly, 2000) under the following conditions: a correlated‐allele model with a 500,000 burn‐in period, 750,000 MCMC reps after burn‐in, *K* from 2 to 8, and 20 iterations. The value of the ad hoc statistics ∆(*K*) was then estimated with Harvester (Earl & von Holdt, 2012) using the average value of LnP(*D*) to estimate the number of genetic groups (Evanno, Regnaut, & Goudet, 2005).

### 2.6 Mitochondrial sequence data analysis

The obtained sequences were manipulated as a raw data set using MEGA 6 (Tamura, Stecher, Peterson, Filipski, & Kumar, 2013), and sequence divergence was estimated. The standard diversity indices (the number of haplotypes and polymorphic sites) were estimated using DnaSP ver. 5.10.01 (Librado & Rozas, 2009), and the raw data set was converted for analysis in Arlequin and NETWORK. The molecular diversity indices (haplotype diversity and nucleotide diversity) were estimated using Arlequin ver. 3.1 (Excoffier et al., 2005). To estimate the genealogical relations among the haplotypes, a median‐joining network was calculated using NETWORK ver. 4.6.1.3 (http://www.fluxus-engineering.com). *F*_ST_ distances among all pairs in the population were used to assess the genetic structure of *L. dispar asiatica*. Population pairwise *F*_ST_ values were calculated using the Kimura two‐parameter model in Arlequin ver. 3.1 (Excoffier et al., 2005) (significance test = 0.05; significance level = 1,000 permutations). To estimate genetic differentiation among regional populations, AMOVA was used following the Kimura two‐parameter model in Arlequin ver. 3.1 (Excoffier et al., 2005). To test the IBD model, the correlation between genetic distance and geographic distance was calculated using Mantel's test with 30,000 randomizations in IBD ver. 3.23 (Jensen et al., 2005).

To estimate the demographic history of the gypsy moth populations, a mismatch distribution analysis was conducted using Arlequin ver. 3.1 (Excoffier et al., 2005). Sudden expansion of the population was first estimated in a mismatch distribution graph: unimodal or not unimodal. Deviation from the demographic expansion model was estimated using the sum of the squared deviation and Harpending's raggedness index (Harpending, 1994). To estimate population equilibrium, Fu's FS test (Fu, 1997) and Tajima's *D* test (Tajima, 1989) were conducted. If population expansion was detected by the mismatch distribution, the expansion time of the population was calculated using the formula tau = 2*ut* (tau = age expansion parameter; *u = *the aggregate mutation rate over the region of DNA under study; and *t *= generation time) (Roger & Harpending, 1992), with the assumption that the mutation rate of insect mitochondrial DNA is 2.3% per million years (Brower, 1994).

## 3 RESULTS

### 3.1 NGS sequencing and microsatellite marker development

Illumina sequencing of the genomic DNA from *L. dispar* eggs obtained 3,974,358,483 bp from 15,988,036 reads, with an average of 248 bp per read, which assembled into 718,940 contigs, with an average of 511 bp per contig (Table  S1). The contigs contained 1,867 microsatellite loci (excluding AT repeats; the length of the repeated base, 2–4 bp; and repeated more than four times). Of these, 430 loci showing more than six repeated motifs were tested for the probability of marker design with PRIMER3 (Koressaar & Remm, 2007; Untergrasser et al., 2012). We were able to design primer sets for 207 loci, from which we randomly selected 150 loci for PCR tests to examine polymorphism. Capillary electrophoresis revealed that 29 of 150 loci showed clear polymorphism (Table 2). From PCR amplification tests on the labeled markers of selected 29 loci, we selected eight microsatellite markers (39,767, 58,587, 124,259, 134,079, 230,995, 297,455, 344,041, and 346,977) (Table 3). The sequences of the eight selected microsatellite loci were submitted to NCBI GenBank (Table 3).

**Table 2:** Selected primer sets (29 of 150) showing clear polymorphism

No	MSL no	SPS	RM
1	19,028	GCGTACAAACTACGCAAGTC	(CT)_14_
		ATAGCCATGAAGCGAGTGTA	
2	20,500	CCCCTAGTCATTCCGTTAAAC	(ATG)_9_
		AGCAAACATTCGACGACTC	
3	22,651	GTGGCAACCGTAGACATAAC	(ATC)_10_
		CGTCTGACCAACGAGATAAA	
4	39,767	AGCGCTTCCTAATTGGTTAT	(GT)_15_
		ACGCGTGGTTATAACTTTCA	
5	44,678	GGATGAAGTTGATGGGTGAG	(ATC)_9_
		CGCGATGCTGATGAAGTTAT	
6	58,587	TGCAGTCGAATTTAGGCAAA	(ATG)_8_
		TTGAACAAAGCCAATCGGAT	
7	109,715	GGGTTTCCTGACTTTGATACA	(AC)_13_
		CTCCATGAGATGACTGGCTA	
8	119,274	GCGACCGGTCATAAAACTAT	(ATG)_9_
		ATTTCTCTCTCACGCCAGT	
9	124,259	TTGACACTGCACCGTAAATT	(AG)_13_
		ATATTGCGCATATGACCCAC	
10	134,079	TGAAAGACGACTAAAGCACG	(ATC)_9_
		GACTCTTGAGCAATTGGGTT	
11	167,938	GAAATTTGCACCAGTTTGAA	(AG)_15_
		TGGCAATGAATTCTGCTTAT	
12	178,435	CTTGCCCGTGAATATCGAAA	(GT)_13_
		AGTTTACATGAAGCGACAGTT	
13	178,855	AATGTCACAGAACGAAGTGG	(GT)_16_
		GGCAACGAATTTGCTTAGTA	
14	203,511	GACTTTAACGAGTGCACAGT	(ACAT)_7_
		TGACCATGAACCAATTAGCG	
15	205,435	GGTGGGGTGTGTTTAGACTA	(CT)_13_
		GGTGATATGGCAGAACAGC	
16	206,922	CCATGAAGCTACAAGTTCGAT	(GT)_13_
		AGGCTATATTTCCTACCGGG	
17	230,995	CCATCTGACCATTGTGCTAT	(ATC)_10_
		TGAGGCACTATGTCCTTGAT	
18	233,404	TTGACAGCCGTTATTGAGAT	(AC)_16_
		AACTACCGCCATCATTATCA	
19	239,543	TTTGTGGCGAAACATGAGAT	(ATG)_8_
		AAACAAACGGGGTAAGCTAAA	
20	243,906	ACGGAACCCTAAAAATGAAC	(ATC)_10_
		TTACCTGGAATGGTCGAATA	
21	253,129	GAGTACCCGACATTGATTGA	(GT)_17_
		AGTGCACGTCTACACTACCG	
22	297,455	GTGTGCGTTCTGTGGTATG	(CT)_23_
		GTGGACTCGCTGTAACACTC	
23	306,436	CGTCTGCGTACTATCATATTGA	(GT)_13_
		GTTGTACTGTTACTCCTCGC	
24	314,848	CTGACCAGCGTATCAATTTC	(AGG)_14_
		ATCAAATACGAACGCGATAA	
25	327,335	TTTTGTTTGTAGTGCCGAAC	(GT)_22_
		CAATATGACCCAACGTCATT	
26	331,393	TTCTCGCAAAACCAAGACC	(ATG)_9_
		AAGTGAATGTTAGCAGGGTG	
27	335,162	ATCTGCTGATATCGCAATGG	(ATC)_8_
		GAGGCAAACAGTGGGATTTA	
28	344,041	GTGGCACGTGAACAAATATAC	(ATC)_9_
		CTTTGCTTGTGGGTGTCATA	
29	346,977	CTTGCTGGACTTATCTGTGG	(AGTC)_8_
		ACGTTTTTCAGTGGGTAGGT	

MSL, microsatellite loci; SPS, sequence of primer set; RM, repeat motif.

**Table 3:** Ten selected markers for microsatellite loci analysis of *Lymantria dispar*

MSL no	Marker name	Sequence	RM	Size	GAn
39,767	39767‐FAM	AGCGCTTCCTAATTGGTTAT	(GT)_15_	129–179	KT633401
	39767R	ACGCGTGGTTATAACTTTCA			
58,587	58587‐FAM	TGCAGTCGAATTTAGGCAAA	(ATG)_8_	214–299	KT633402
	58587R	TTGAACAAAGCCAATCGGAT			
124,259	124259‐FAM	TTGACACTGCACCGTAAATT	(AG)_13_	184–218	KT633403
	124259R	ATATTGCGCATATGACCCAC			
134,079	134079‐FAM	TGAAAGACGACTAAAGCACG	(ATC)_9_	159–270	KT633404
	134079R	GACTCTTGAGCAATTGGGTT			
230,995	230995‐FAM	CCATCTGACCATTGTGCTAT	(ATC)_10_	148–196	KT633405
	230995R	TGAGGCACTATGTCCTTGAT			
297,455	297455‐FAM	GTGTGCGTTCTGTGGTATG	(CT)_23_	170–254	KT633407
	297455R	GTGGACTCGCTGTAACACTC			
344,041	344041‐FAM	GTGGCACGTGAACAAATATAC	(ATC)_9_	131–329	KT633409
	344041R	CTTTGCTTGTGGGTGTCATA			
346,977	346977‐FAM	CTTGCTGGACTTATCTGTGG	(AGTC)_8_	165–201	KT633410
	346977R	ACGTTTTTCAGTGGGTAGGT			

MSL, microsatellite loci; RM, repeat motif; GAn, GenBank accession number.

### 3.2 Microsatellite marker assessment

Microsatellite markers were initially assessed using 20 regional populations (one population from Mongolia, one from China, one from Russia, 12 from Korea, and five from Japan). Testing for genotyping errors at each locus revealed that one marker (346,977 in Site 22) showed evidence of null alleles (Table 4). However, the null allele frequency of the marker was 0.1947, which is lower than 0.20. In microsatellite analysis, the frequencies of null alleles are almost always *p *<* *.40 and usually *p *<* *.20 (Dakin & Avise, 2004). When microsatellite null alleles are uncommon to rare (*p *<* *.20), their presence causes a slight underestimate of the average exclusion probability at a locus; however, this is usually not of sufficient magnitude to warrant great concern. For *p *>* *.20, however, the mean “estimated with null” exclusion probability can be much higher than the “true” and “estimated without null” values (Dakin & Avise, 2004). Therefore, we retained the marker showing *p *<* *.20 in our population genetic analysis. We estimated that these markers were independently evolved within our samples; however, the tests for linkage disequilibrium were not significant. Therefore, genetic diversity indices (major allele frequency, genotype number, allele number, gene diversity, observed heterozygosity, and PIC) of each regional population were estimated with eight markers. We determined the allele type frequencies based on microsatellite loci for each population (Table  S2). For each marker, gene diversity, observed heterozygosity, and PIC ranged from 0.2500 to 0.8963, 0.2778 to 1.0000, and 0.2374 to 0.8877, respectively. The genetic diversity was high, ranging from 2 to 16 alleles for each marker (Table S3). The exact *p‐*values of HWE were calculated for each regional population after sequential Bonferroni corrections (*p *=* *.0003). Deviations from HWE were not detected in the exact *p‐*values; however, some markers were identified as relatively low exact *p‐*values in each population (Table S3). Thus, we compared the differences between gene diversity and observed heterozygosity in each population for each marker. Two populations, Sites 12 and 26, had observed heterozygosity values lower than the gene diversity values in all loci except locus 344,041. Lower observed heterozygosity values than the gene diversity values suggest significant homozygosity, and this implies the presence of null alleles or allelic dropout, linkage of alleles, or inbreeding (Damm, Armstrong, Arjo, & Piaggio, 2015). However, we tested for the presence of null alleles or allelic dropout and linkage of alleles through the previous analyses. Lastly, if the violation were a consequence of inbreeding, we would have expected to observe such a phenotype at many or all loci, not just at a single locus (Damm et al., 2015; Selkoe & Toonen, 2006). The samples from Sites 12 and 26 might indicate inbreeding or sib sampling. In this study, however, we retained the samples from the two sites in our analysis because the deviation from HWE was not detected at all analyzed loci (Table S3). Therefore, we suggest that the developed eight novel microsatellite markers may be useful for a population genetic analysis of *L. dispar*.

**Table 4:** Results of the test of null alleles and the PCR error present in eight filtered markers

MSL	Site 1		Site 6		Site 10		Site 12	
	NP	Freq	NP	Freq	NP	Freq	NP	Freq
39,767	No	0.0164	No	−0.0053	No	−0.1204	No	0.0735
58,587	No	−0.1305	No	−0.5138	No	−0.4287	No	0.0323
124,259	No	−0.0127	No	0.0763	No	−0.0307	No	0.0725
134,079	No	−0.0313	No	−0.1476	No	−0.0360	No	0.0456
230,995	No	−0.0139	No	0.0417	No	−0.0508	No	−0.0014
297,455	No	−0.0548	No	−0.0010	No	−0.0810	No	0.0379
344,041	No	−0.1074	No	−0.0994	No	−0.1225	No	−0.1475
346,977	No	−0.0233	No	0.0644	No	−0.0468	No	0.0002

MSL	Site 16		Site 18		Site 22		Site 26	
	NP	Freq	NP	Freq	NP	Freq	NP	Freq
39,767	No	0.0704	No	0.0236	Yes	0.1386	No	0.0301
58,587	No	−0.2454	No	−0.4557	No	0.0147	No	0.0096
124,259	No	0.0164	No	−0.0084	No	−0.0284	No	0.1223
134,079	No	−0.0530	No	0.0206	No	0.0575	Yes	0.1540
230,995	No	0.0303	No	−0.0086	No	−0.0073	No	0.0995
297,455	No	0.0070	No	−0.0123	No	0.0362	No	0.0708
344,041	No	−0.1725	No	−0.1539	No	−0.1185	No	−0.1949
346,977	No	−0.0357	No	0.0835	Yes	0.1947	No	0.1397

MSL	Site 27		Site 28		Site 30		Site 31	
	NP	Freq	NP	Freq	NP	Freq	NP	Freq
39,767	No	0.0022	No	−0.1312	No	0.0307	No	0.1159
58,587	No	0.0443	No	0.1366	No	0.1281	No	0.0343
124,259	No	−0.0552	No	0.0166	No	−0.0625	No	−0.1003
134,079	No	−0.1057	No	−0.0089	No	−0.0659	No	−0.0150
230,995	No	−0.0280	Yes	0.1603	No	0.0402	No	−0.0294
297,455	No	0.0071	No	0.0192	No	0.0271	No	−0.0036
344,041	No	−0.5174	No	−0.2371	No	−0.1892	No	−0.1916
346,977	No	0.0421	No	0.0802	No	0.0023	No	0.1520

MSL	Site 33		Site 34		Site 35		Site 36	
	NP	Freq	NP	Freq	NP	Freq	NP	Freq
39,767	No	0.0554	No	−0.1725	No	−0.0752	No	−0.0275
58,587	No	−0.0017	No	−0.0801	No	0.0340	No	0.0901
124,259	No	0.0812	No	0.0861	No	0.0094	No	−0.1114
134,079	No	−0.0264	No	−0.1606	No	0.0087	No	0.0502
230,995	Yes	0.0980	No	−0.6078	No	−0.0350	No	−0.0157
297,455	No	0.0196	No	−0.0727	No	−0.2100	No	−0.0614
344,041	No	−0.0685	No	−0.1697	No	−0.2168	No	−0.3067
346,977	No	−0.0182	No	0.0000	No	0.0964	No	0.0824

MSL	Site 37		Site 38		Site 39		Site 41	
	NP	Freq	NP	Freq	NP	Freq	NP	Freq
39,767	No	0.0402	No	−0.0001	No	0.0300	No	0.0332
58,587	No	−0.0135	No	−0.0135	No	−0.1435	No	−0.0089
124,259	No	0.0296	No	0.0049	No	−0.0051	No	−0.1271
134,079	Yes	0.1016	No	0.0658	No	−0.0389	No	0.0774
230,995	No	−0.0234	No	−0.0062	No	−0.0010	No	0.0138
297,455	No	−0.1981	No	−0.1566	No	−0.0962	No	−0.0671
344,041	No	−0.1294	No	−0.2479	No	−0.1748	No	−0.5257
346,977	No	0.0093	No	−0.1832	No	−0.0479	No	−0.4195

MSL, microsatellite loci; NP, null present; Freq, null allele frequency.

### 3.3 Population structure using microsatellite loci

#### 3.3.1 Pairwise *F*_ST_ genetic distances

**Figure 3:**
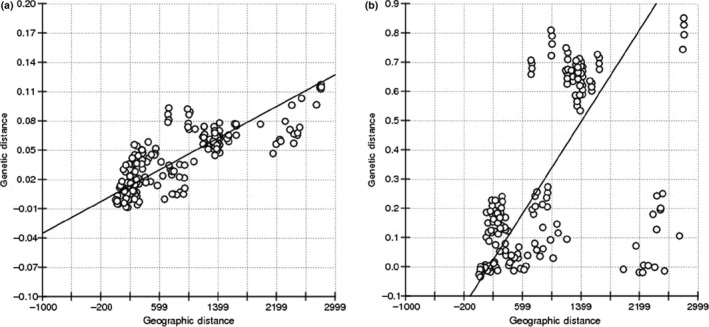
Isolation by distance for matrix correlation between genetic distance and geographic distance (a, microsatellite loci, *r *=* *0.7909, *p *=* *.0000; b, mitochondrial genes, *r *=* *0.5312, *p *=* *.0006)

The population genetic structure of *L. dispar* in Far East Asia was calculated with *F*_ST_ values. Pairwise *F*_ST_ distances among regional populations ranged from −0.0087 to 0.1171 (Table 5: Lower side). Considering the genetic distances in each geographical region, the regional populations in Hokkaido (Sites 35–38), the species origin region, showed relatively low genetic distances (from −0.0055 to −0.0010); however, compared to other regional populations, their genetic distance was relatively high (0.0472 to 0.1171). A Mongolian regional population (Site 34), which was further from Hokkaido than other regional populations, showed relatively high genetic distances of 0.0464 to 0.1171 from other regional populations. A Vladivostok population (Site 33) was similar to a Korean inland population (Site 6) in population genetic structure (*F*_ST_ = −0.00006), yet showed a high genetic distance of 0.1030 when compared to a Jeju regional population (Site 31). A Chinese regional population (Site 39) was more similar to a Korean inland population (Site 28) with a relatively large geographical distance (genetic distance: 0.0050 and geographic distance: 776.35 km) than to a Russian population (Site 33) with a relatively short distance (genetic distance: 0.0199 and geographic distance: 289.10 km). A Kyushu regional population (Site 41) with a small sample size had a genetic distance of 0.0307 to 0.0964 from other populations. Lastly, the Korean inland populations (Sites 1–30) ranged from −0.0087 to 0.0358 and were similar in genetic structure. In the analysis of isolation by distance, genetic distance increased with increasing geographic distance (Figure 3a, *r* = 0.7909, *p *=* *.0000).

**Table 5:** Pairwise *F*_ST_ distances among regional populations of *Lymantria dispar* in Far East Asia

RP	Site 1	Site 6	Site 10	Site 12	Site 16	Site 18	Site 22	Site 26	Site 27	Site 28	Site 30	Site 31	Site 33	Site 34	Site 35	Site 36	Site 37	Site 38	Site 39	Site 41
Site 1	–	−0.00406	−0.01463	0.00437	−0.01334	0.30988	0.26683	0.34350	0.36249	0.37666	0.42026	0.05208	−0.01758	0.01696	0.06167	0.03623	0.05189	0.05030	−0.01908	0.09112
Site 6	0.0082	–	0.02061	0.04141	0.01087	0.31302	0.27210	0.34671	0.36975	0.37793	0.42483	0.03678	−0.01293	−0.02022	0.01923	−0.00276	0.01680	0.01657	−0.03187	0.08292
Site 10	0.0041	0.0046	–	−0.00547	−0.00048	0.27709	0.23826	0.31041	0.32849	0.34123	0.38091	0.06691	0.00744	0.04545	0.07984	0.06011	0.08359	0.08000	0.01072	0.05150
Site 12	0.0095	−0.0087	0.0047	–	−0.01072	0.19614	0.16332	0.23044	0.24587	0.25756	0.28768	0.06457	0.02960	0.05829	0.09211	0.08333	0.10071	0.09608	0.02959	0.04092
Site 16	0.0215	0.0085	0.0249	0.0023	–	0.24157	0.20618	0.27773	0.29740	0.30568	0.34216	0.04450	0.00193	0.02222	0.06184	0.04432	0.05908	0.05632	−0.00227	0.05484
Site 18	0.0154	0.0044	0.0074	0.0048	0.0179	–	−0.02895	−0.02244	0.00001	−0.02079	−0.01230	0.16783	0.34885	0.33869	0.32523	0.34119	0.37860	0.36973	0.33390	0.26957
Site 22	0.0211	0.0223	0.0096	0.0350	0.0427	0.0082	–	−0.01622	0.00406	−0.01105	−0.00127	0.14439	0.29900	0.28227	0.28579	0.29604	0.32803	0.31966	0.27852	0.22710
Site 26	0.0067	0.0132	0.0076	0.0172	0.0302	0.0055	−0.0002	–	−0.01407	−0.02450	−0.02393	0.20924	0.37848	0.35742	0.35378	0.36854	0.40218	0.39334	0.35680	0.28884
Site 27	0.0195	0.0167	0.0135	0.0278	0.0358	0.0116	−0.0082	−0.0041	–	−0.00950	−0.01735	0.24545	0.39655	0.36943	0.37486	0.38864	0.41977	0.41071	0.36976	0.29797
Site 28	0.0024	−0.0061	0.0083	−0.0011	0.0167	0.0079	0.0233	0.0031	0.0136	–	−0.02665	0.22708	0.41510	0.40396	0.38466	0.40379	0.44125	0.43236	0.40187	0.32700
Site 30	0.0153	−0.0029	0.0146	−0.0006	−0.0006	0.0004	0.0158	0.0130	0.0180	0.0034	–	0.26873	0.46777	0.46701	0.43298	0.45685	0.49709	0.48818	0.46079	0.37824
Site 31	0.0418	0.0463	0.0502	0.0470	0.0582	0.0280	0.0347	0.0359	0.0451	0.0542	0.0364	–	0.05305	0.01264	0.05657	0.03449	0.04812	0.04617	0.02447	0.05201
Site 33	0.0050	−0.0003	0.0069	0.0043	0.0248	0.0215	0.0383	0.0225	0.0346	0.0049	0.0110	0.0629	–	0.01113	0.06291	0.02699	0.04549	0.04535	−0.03346	0.13820
Site 34	0.0561	0.0607	0.0590	0.0794	0.0960	0.0709	0.0569	0.0651	0.0664	0.0676	0.0732	0.1030	0.0464	–	0.02929	−0.02552	0.00137	0.00592	−0.01196	0.15323
Site 35	0.0585	0.0738	0.0698	0.0616	0.0619	0.0554	0.0639	0.0582	0.0626	0.0676	0.0624	0.0748	0.0865	0.1141	–	−0.00877	0.01998	0.01630	0.03854	0.05446
Site 36	0.0558	0.0659	0.0642	0.0594	0.0572	0.0472	0.0588	0.0568	0.0572	0.0591	0.0533	0.0652	0.0809	0.1171	−0.0012	–	−0.01555	−0.01582	−0.00622	0.08947
Site 37	0.0510	0.0636	0.0643	0.0557	0.0560	0.0526	0.0617	0.0509	0.0568	0.0575	0.0588	0.0769	0.0784	0.1127	−0.0055	−0.0051	–	−0.03829	0.01818	0.11981
Site 38	0.0655	0.0741	0.0779	0.0739	0.0662	0.0618	0.0654	0.0610	0.0627	0.0697	0.0676	0.0767	0.0931	0.1144	−0.0038	−0.0010	−0.0022	–	0.01992	0.10472
Site 39	0.0137	0.0178	0.0167	0.0237	0.0380	0.0302	0.0353	0.0345	0.0282	0.0050	0.0283	0.0890	0.0199	0.0766	0.0774	0.0713	0.0735	0.0863	–	0.13201
Site 41	0.0516	0.0448	0.0453	0.0380	0.0467	0.0398	0.0307	0.0359	0.0442	0.0555	0.0418	0.0501	0.0715	0.0964	0.0494	0.0550	0.0445	0.0513	0.0921	–

RP, regional population; lower side, microsatellite loci; upper side, mitochondrial genes.

#### 3.3.2 NeighborNet network

**Figure 4:**
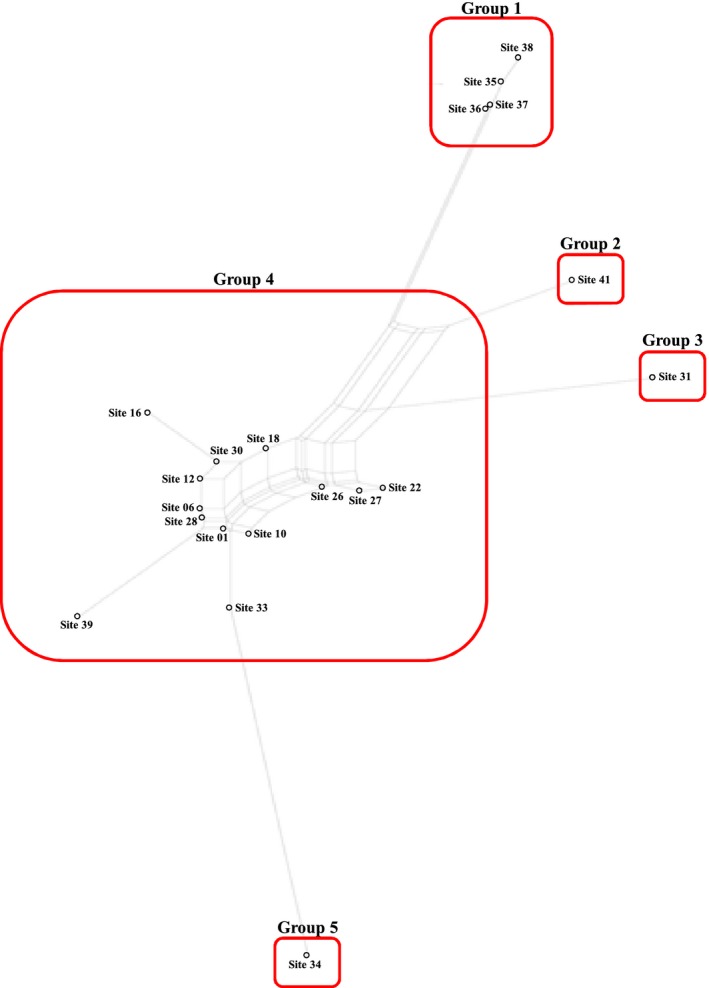
NeighborNet network using pairwise *F*_ST_ distances from 20 regional populations of *Lymantria dispar* from Far East Asia

In a NeighborNet network based on pairwise *F*_ST_ genetic distances, the 20 regional populations could be divided into five groups: Group 1, Hokkaido (Sites 35, 36, 37, and 38); Group 2, Kyushu (Site 41); Group 3, Jeju Island (Site 31); Group 4, Korean Peninsula and adjacent areas (Sites 1, 6, 10, 12, 16, 18, 22, 26, 27, 28, 30, 33, and 39); and Group 5, Mongolia Selenge (Site 34) (Figure 4). Among these sites, Sites 28 and 30 from inland Korea were closest to each other in geographic distance; however, their genetic distance was similar to Site 1 (geographically close to Incheon Harbor) and Site 16 (geographically close to Uljin Harbor). These two regions are geographically close to Busan Harbor, which is a frequent entry port for vessels (Choi, 2014). We therefore suspect that these two regional populations may frequently interbreed with the regional populations near Incheon Harbor and Uljin Harbor.

#### 3.3.3 Bayesian clustering

**Figure 5:**
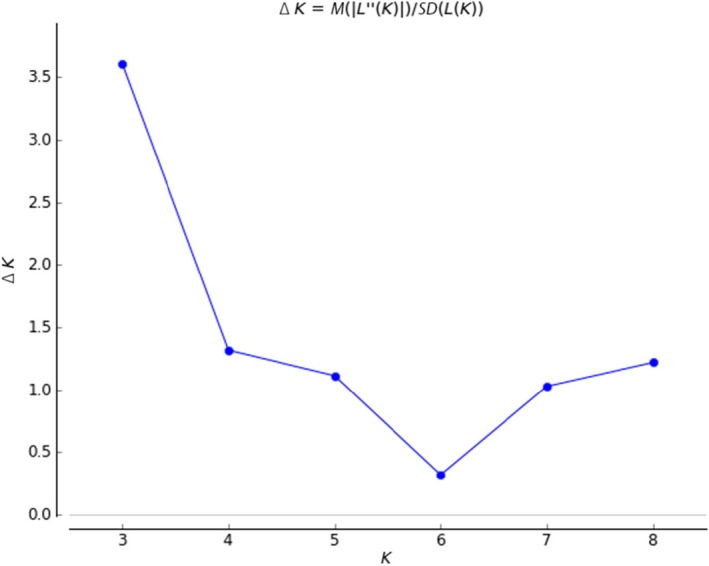
The ad hoc statistics Δ(*K*) on the basis of LnP(D) estimated from 20 iterations for each *K*. The ad hoc statistics exhibited a signal of at best *K *=* *3

**Figure 6:**
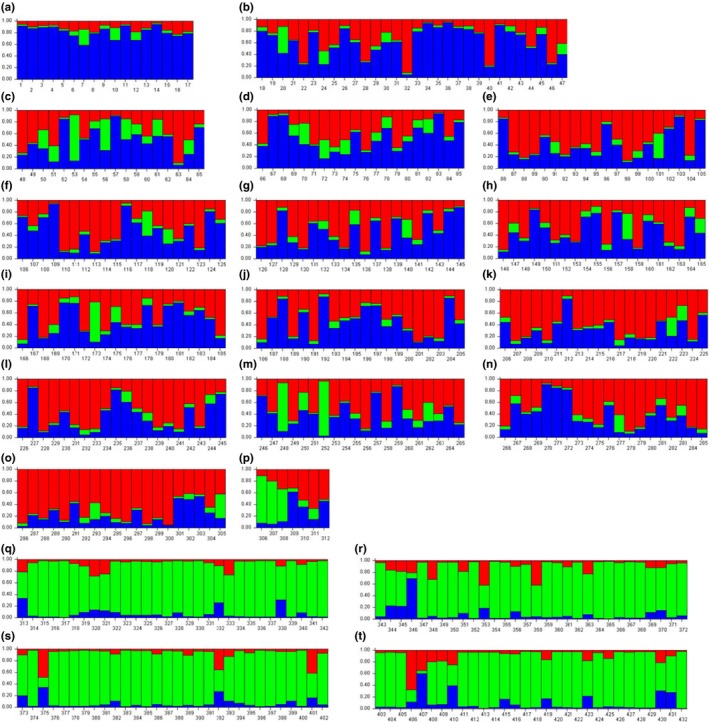
Bar plots estimated by STRUCTURE. The best *K* was estimated as three based on the ad hoc statistics Δ(*K*) (a, Site 34; b, Site 33; c, Site 39; d, Site 01; e, Site 10; f, Site 28; g, Site 30; h, Site 16; i, Site 12; j, Site 06; k, Site 18; l, Site 22; m, Site 27; n, Site 26; o, Site 31; p, Site 41; q, Site 38; r, Site 35; s, Site 37; t, Site 36)

For the model‐based Bayesian analysis, *K* was estimated by varying it from two to eight, and the ad hoc statistics ∆(*K*) (Evanno et al., 2005) indicate the maximum level of structure in three genetic groups (Figure 5). *Lymantria dispar* has been divided into two subspecies in Asia, *L. dispar asiatica* (or *L. dispar dispar*) and *L. dispar japonica*, based on mitochondrial DNA and microsatellite analysis (Bogdanowicz et al., 2000; Wu et al., 2015). Our study showed similar results; however, the Far East Asian gypsy moth populations were distinguishable as three types according to sampling region (Figure 6). Comparing the individual colored bar plots among the regional populations revealed that the frequency of the green‐colored genetic content was high in Hokkaido regional populations (the species origin region) (Figures 6q, r, s, and t), the frequency of the red genetic content was high in Jeju regional populations (Figure 6o), and the frequency of the blue genetic content was high in Mongolian regional populations (Figure 6a). The regional populations from the Korean Peninsula and adjacent areas showed a mixed pattern in comparison with the Jeju regional populations and Mongolian regional populations.

Comparing the individual colored bar plots of each regional population, Sites 35, 36, 37, and 38 from Hokkaido were clearly distinct in genetic makeup from the regional populations of the Korean Peninsula and adjacent areas (Figure 6). Several individuals (Figure 6r: individual 346; Figure 6s: individual 375; and Figure 6t: individuals 406, 407, and 410) showed a genetic makeup similar to that of other regional populations; however, in the majority of individuals, the main genetic makeup was the green‐colored one. A genetic content frequency similar to that of the Hokkaido regional populations could be seen in Site 41 (Kyushu population), Site 27 (Hapcheon population), Site 12 (Cheongwon population), and Site 39 (Jilin population). Among them, the Kyushu regional population, with only seven individuals analyzed, was divided into two types: three individuals showed features similar to the Hokkaido regional populations, and four individuals showed features similar to the Jeju regional populations (Figure 6p).

The individual colored bar plots of the Korean inland populations show high frequencies of the blue or red genetic content in each individual. These two genetic content types showed similar frequencies in several individuals. This result may be caused by the higher genetic diversity in these populations than in other regions, and the gene flow among the Korean inland regions may be relatively higher than with other regions (Table 6, *F*_ST_ = 0.04192). In the Chinese and Russian regional populations, however, the blue genetic content was higher than other genetic content types. Several individuals (Figure 6i: individual 173; Figure 6m: individuals 248 and 262; and Figure 6c: individuals 53 and 56) had features similar to those of the Hokkaido regional populations.

**Table 6:** AMOVA for microsatellites and mitochondrial genes of *Lymantria dispar* from Far East Asia

Source of variation	Sum of squares	Variance components	Percentage of variation	*F*‐statistics	*p*‐value
MS					
Among local sites	158.192	0.12761	4.19163	*F*_ST_ = 0.04192	.00000
Among individuals within local sites	1180.222	−0.02834	−0.93072	*F*_IS_ = −0.00971	.85239
Within individuals	1262.500	2.94520	96.73909	*F*_IT_ = 0.03261	.00000
MT					
Among groups	177.317	0.57813 Va	48.28000	*F*_CT_ = 0.48280	.00000
Among local sites within groups	12.714	0.00565 Vb	0.47000	*F*_SC_ = 0.00913	.04790
Within local sites	282.283	0.61366 Vc	51.25000	*F*_ST_ = 0.48753	.00000

MS, microsatellite loci; MT, mitochondrial genes.

### 3.4 Population structure using mitochondrial DNA

#### 3.4.1 Mitochondrial DNA sequence variation

DNA barcodes of the COI, ATP6, and ATP8 genes were sequenced from 480 of 552 *L. dispar asiatica* samples collected from the 20 study sites (*n* = 6–30 per site). Mitochondrial DNA sequence divergences obtained from the 480 samples ranged from null to 0.5%, with 98 haplotypes distinguished by 85 polymorphic sites (Table S4). The mean gene diversity was 0.6529 ± 0.0929 (lowest value 0.1538 ± 0.1261 from Site 34 and highest value 0.9407 ± 0.0432 from Site 12), and the mean nucleotide diversity was 0.013798 ± 0.010223 (lowest value 0.001810 ± 0.003024 from Site 34 and highest value 0.027156 ± 0.017203 from Site 12) (Table S5).

#### 3.4.2 Mitochondrial genealogy

**Figure 7:**
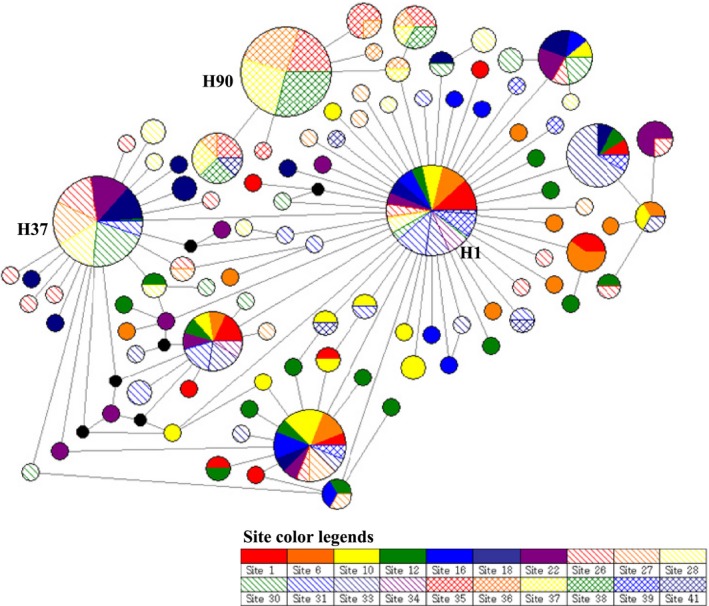
Median‐joining network using mitochondrial genes of *Lymantria dispar* from Far East Asia

In the median‐joining network, three high‐frequency haplotypes (H1, 151ex; H37, 75ex; and H90, 73ex) were connected to each other by low‐frequency haplotypes (Figure 7). This pattern was revealed in the pairwise *F*_ST_ distances (Table 5: Upper side). We found that the 20 studied populations of *L. dispar* were divided into three groups according to genetic distance: Group 1, Korean inland region and adjacent areas (Sites 01, 06, 10, 12, 16, 31, 33, 34, 39, and 41); Group 2, Korean southern region (Sites 18, 22, 26, 27, 28, and 30); and Group 3, Hokkaido region (Sites 35, 36, 37, and 38) (Table S5, *F*_CT_ = 0.48280, *F*_ST_ = 0.48753). The results of the analysis of IBD were similar to the microsatellite results (Figure 3b, *r* = 0.5312, *p *=* *.0006). In particular, haplotype H90 appeared only in Hokkaido regional populations and was connected with haplotype H1 by haplotype H95, which is another Hokkaido haplotype (Figure 7). The Kyushu regional population (Site 41) contained five haplotypes, of which three haplotypes showed in the inland (H1, 2ex; H27, 1ex; and H82, 1ex), one in Hokkaido (H93, 1ex), and one in only Kyushu (H98, 1ex). One of the high‐frequency haplotypes, H1, was distributed in all the inland collecting regions and was detected in an individual from Site 38 in Hokkaido (Figure 7). Haplotype H1 was connected with Haplotype H37, a high‐frequency haplotype in the southern area of the Korean Peninsula, by low‐frequency haplotypes. Overall, the Far East Asian gypsy moth populations showed a star‐shaped network in which three high‐frequency haplotypes (H1, 151ex; H37, 75ex; and H90, 73ex) were connected with each other through low‐frequency haplotypes (Figure 7). Therefore, the Far East Asian gypsy moth populations may have undergone sudden population expansion.

#### 3.4.3 Mitochondrial DNA haplotype mismatch distribution

**Figure 8:**
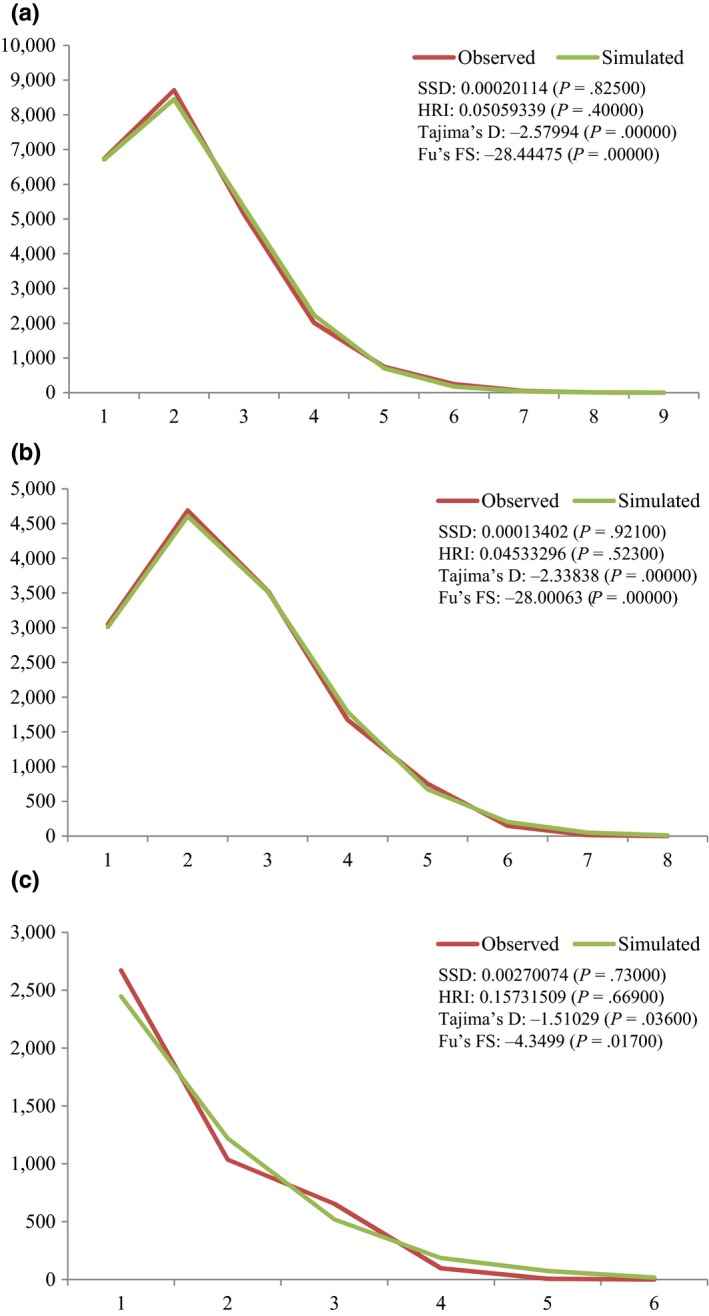
The mismatch distributions of each group of *Lymantria dispar* from Far East Asia (a, Group 1; b, Group 2; c, Group 3)

The median‐joining network revealed a star‐shaped mtDNA genealogy, so we analyzed the mismatch distribution, applying a sudden population expansion model. We conducted the analysis using the three groups recognized above, and we found that the mismatch graphs of the groups were unimodal and the mismatch parameters were insignificant (Figure 8). In neutral equilibrium, Tajima's D and Fu's FS tests also had negative values in all three groups (Figure 8). We therefore consider that the mismatch analysis supports a sudden population expansion.

The expansion time of each group was inferred using the observed value of the age expansion parameter (tau), the equation *t* = tau/2u (Roger & Harpending, 1992), and an insect mtDNA mutation rate of 2.3% per MY per lineage for silent sites (Brower, 1994). The tau of each group was 1.234 in Group 1, 1.496 in Group 2, and 1.750 in Group 3, and the expansion times were estimated to be 53,652 generations ago in Group 1, 65,043 in Group 2, and 76,086 in Group 3. Considering that *L. dispar* produces one generation per year (Pogue & Schaefer, 2007), the population expansion time of each group in Far East Asia was inferred to be approximately 53,652 years before present (ybp) in Group 1, 65,043 ybp in Group 2, and 76,086 ybp in Group 3.

## 4 DISCUSSION

The taxonomic status of the two subspecies of *L. dispar* in Far East Asia has been debated (Arimoto & Iwaizumi, 2014; Pogue & Schaefer, 2007; Schintlmeister, 2004). In a recent study using molecular data (Wu et al., 2015), *L. dispar dispar* (European subspecies) was clearly distinct from the Asian two subspecies; however, the Asian subspecies were difficult to distinguish from each other. The Japanese subspecies, *L. dispar japonica*, was genetically similar to the populations from the southern end of the Korean Peninsula, and the Korean populations had mixed genetic content (Wu et al., 2015). We examined the previous study's collecting sites and found they were mainly located near seaports. We therefore included inland populations in the present study (Figure 2).

In mitochondrial genealogy, we found that three lineages of *L. dispar* were distributed in Far East Asia: two in the Korean Peninsula and adjacent inland areas, and one in Hokkaido, Japan. Inferring the demographic history of each lineage through mismatch analysis, Group 1 expanded suddenly approximately 53,652 ybp, Group 2 approximately 65,043 ybp, and Group 3 approximately 76,086 ybp, all within the Würm glacial period (110,000–12,000 ybp) (Gao, Hou, & Guo, 2016).

The Würm glacial period can be divided into three glacial stages and two subinterglacial stages (Gao et al., 2016; Han & Meng, 1996; Ma, Yu, Wang, & Yao, 2006). The mean temperature during the period was approximately 5°C lower than at present, based on the snow‐line elevation on Mt. Fuji in Japan (Kim, 2011). In Europe, three ice sheets (Scandinavian, British, and Alpine) developed to cover a large part of the continent (Trojan, 1997). The advancing glacier forced the flora and fauna of the warm and temperate zones southward, and refugia were formed in the Mediterranean region (Trojan, 1997). In a great amount of Siberia, large ice masses eliminated all plants and animals; however, eastern regions (including Ussuri Land, Korea, Manchuria, and Japan) remained ice‐free as fauna‐ and flora‐preserving areas during the glaciation period (Trojan, 1997). During these periods, the flora of the southern part of Korea showed the features of a cool, temperate climate (Chung, Lee, Lim, & Kim, 2005). For example, Polypodiaceae, *Alnus* spp., *Carpinus* spp., and deciduous *Quercus* spp. were distributed in the area (Chung et al., 2005; Kim, 2011). *Carpinus* spp. and *Quercus* spp. are the food plants of *L. dispar asiatica* in Korea (Lee et al., 2002). During the last glacial maximum (approximately 20,000–18,000 ybp), however, *Picea* spp., *Abies* spp., *Pinus* spp., and *Larix* spp. were distributed in Far East Asia as it changed to a subarctic climate (Kim, 2011; Yoon & Hwang, 2009). The coastline during this period was quite different from the present. The west sea of the Korean Peninsula was a low hilly area because the sea level was approximately 30–130 m below present levels (Kim, 2011; Park & Cho, 1998; Park, Yoo, Lee, & Lee, 2000). The Japanese islands were connected with Sakhalin and the southeast part of the Korean Peninsula by a land bridge (Park et al., 2000; Trojan, 1997).

The sudden expansion of the Japanese Hokkaido lineage (Group 3) may have taken place in the middle of the Würm glacial stage I (approximately 76,086 ybp), a period with a cold and dry climate that might have led them to move to more southern regions. The southern lineage of the Korean Peninsula (Group 2) might have expanded during the late Würm glacial stage I (approximately 65,043 ybp). During this period, the gypsy moth populations might have dispersed into the southern part of the Korean Peninsula because of the cold climate. Lastly, Group 1 might have dispersed into the Vladivostok area, the middle region of Korea, and even Mongolia because the estimated expansion period is approximately 53,652 ybp, which is known as the subinterglacial stage I (60,000–50,000 ybp), a period with a hot and wet climate. We can thus infer that the gypsy moth populations dispersed from Far East Asia into middle Asia. In the Korean Peninsula, however, they may not have dispersed southward because the Noryeong and Charyeong Mountains were formed in the Miocene (Park & Son, 2008), and therefore, genetic interaction between Group 1 and Group 2 would not have been possible. In the model‐based Bayesian analysis using microsatellite loci, *K* (assumed as the number of populations) was calculated to be three, the same as the number of groups examined in the mitochondrial genealogy. The genetic diversity of the regional populations was higher in the Korean Peninsula than in other regions, with the Korean Peninsula populations showing the same mixed pattern reported previously (Wu et al., 2015). We suggest that this genetic pattern might have been caused by multiple sudden population expansions, and the demographic patterns caused by the Würm glacial period may have resulted in the present genetic diversity. In genetic makeup, however, the regional populations near the Busan seaport (Sites 27 and 28) were similar to the middle area of the Korean Peninsula. This might have been caused by vessels arriving in Korea and anchoring at the ports of Incheon or Busan (Choi, 2014; Kim, Kim, Kim, & Lee, 2008). We also looked for this genetic pattern in several samples from Russian and Japanese populations (Figures 6b, p, q, r, s, and t). Thus, we suggest that several individuals might have been introduced into each region via vessels arriving at seaports.

We can suggest that *L. dispar* in Far East Asia are divided into two types (the inland type and the Hokkaido type), although the analyzed samples did not cover the full distributional region of the species in Far East Asia. Taxonomically, 15 nomino‐subspecies have been assigned to *L. dispar*:* L. dispar dispar* Linnaeus, 1758 (type locality [TL]: Europe); *L. dispar erebus* Thierry Mieg, 1886 (TL: England, Us proviennent de Darlington); *L. dispar asiatica* Vnukovskij, 1926 (TL: Russia, Siberia meridionales, Altaij et Sajan occidentalies, Prov. Semipalatinsk); *L. dispar praeterea* Kardakoff, 1928 (TL: Russia, Ussuri‐Gebiet, “Russ. Insel und in Narwa”); *L. dispar hokkaidoensis* Goldschmidt, 1940 (TL: Japan, Hokkaido); *L. dispar koreibia* Bryk, 1948 (TL: Korea, Motojondo); *L. dispar kolthoffi* Bryk, 1948 (TL: China, Kiangsu [=Jiangxu]); *L. dispar andalusica* Reinig, 1938 (TL: Spain, Sierra de Alfacar); *L. dispar mediterraneae* Goldschmidt, 1940 (TL: Southern Europe); *L. dispar bocharae* Goldschmidt, 1940 (TL: Turkestan); *L. dispar chosensis* Goldschmidt, 1940 (TL: Korea); *L. dispar japonica* Motschulsky, [1861] (TL: Japan); *L. dispar umbrosa* Butler, 1881 (TL: Japan, Tokei, Yokohama, Hakodate); *L. dispar hadina* Butler, 1881 (TL: Honshu, Yokohama); *L. dispar obscura* Goldschmidt, 1940 (TL: Japan, Honshu); and *L. dispar nesiobia* Bryk, 1942 (TL: Japan, Kuril Island). Most of these nomino‐subspecies were synonymized and merged into two subspecies, *L. dispar dispar* and *L. dispar japonica*, by Schintlmeister (2004) from morphological analysis and consideration of the type locality of each subspecies. Recently, Pogue and Schaefer (2007) reinstated *L. dispar asiatica* and suggested a three subspecies system (*L. dispar dispar*,* L. dispar asiatica*, and *L. dispar japonica*). We partly agree with Schintlmeister's (2004) view that *L. dispar asiatica* is a synonym of *L. dispar dispar* because the type locality of *L. dispar asiatica* is close to Europe, which is the type locality of *L. dispar dispar*. However, we consider that thorough genetic analyses on regional populations have to be undertaken in other regions of Eurasia to characterize the lineages of gypsy moth across its native range. A taxonomic system of the subspecies of *L. dispar* could therefore be re‐established if each regional lineage revealed by genetic analysis is analyzed and compared with the topotypes collected from the type locality of each subspecies.

## DATA ACCESSIBILITY

DNA sequences of 10 selected microsatellite loci are available in GenBank (KT633401–KT633410); DNA sequences of COI gene are available in GenBank (KT245170–KT246075; KX945391–KX945521); DNA sequences of ATP6/ATP8 gene are available in GenBank (KX945522–KX946001).

## AUTHOR CONTRIBUTIONS

Tae Hwa Kang participated in the correction of the main idea of the study, coordinated the experiment, collected samples, participated in the analysis on the genetic diversity, and drafted the manuscript; Sang Hoon Han participated in the correction of the main idea of the study, coordinated the experiment, and participated in the analysis on the genetic diversity; Heung Sik Lee participated in the design of the main idea the study, collected samples, and managed funding.

## References

[ece33467-bib-0001] Arimoto, M. , & Iwaizumi, R. (2014). Identification of Japanese *Lymantria* species (Lepidoptera: Lymantriidae) based on morphological characteristics of adults. Research Bulletin of the Plant Protection Service Japan, 50, 89–110.

[ece33467-bib-0002] Balloux, F. , & Lugon‐Moulin, N. (2002). The estimation of population differentiation with microsatellite markers. Molecular Ecology, 11, 155–165. 11856418 10.1046/j.0962-1083.2001.01436.x

[ece33467-bib-0003] Bogdanowicz, S. M. , Mastro, V. C. , Prasher, D. C. , & Harrison, R. G. (1997). Microsatellite DNA variation among Asian and North American gypsy moths (Lepidoptera: Lymantridae). Systematics, 90, 768–775.

[ece33467-bib-0004] Bogdanowicz, S. M. , Schaefer, P. W. , & Harrison, R. G. (2000). Mitochondrial DNA variation among worldwide populations of gypsy moths, *Lymantria dispar* . Molecular Phylogenetics and Evolution, 15, 487–495. 10860656 10.1006/mpev.1999.0744

[ece33467-bib-0005] Brower, A. V. Z. (1994). Rapid morphological radiation and convergence among races of the butterfly *Heliconius erato* inferred from patterns of mitochondrial DNA evolution. Proceedings of the National Academy of Sciences of the United States of America, 91, 6491–6495. 8022810 10.1073/pnas.91.14.6491 PMC44228

[ece33467-bib-0006] Bryk, F. (1942). Zur Kenntnis der Großschmetterlinge der Kurilen. Deutsche Entomologische Zeitschrift Iris, 56, 3–90.

[ece33467-bib-0007] Bryk, F. (1948 [1949]). Zur Kenntnis der Großschmetterlinge von Korea. Arkiv för Zoologi, 41, 1–225.

[ece33467-bib-0008] Butler, A. G. (1881). Descriptions of new genera and species of heterocerous Lepidoptera from Japan. Transactions of the Entomological Society of London, 1881, 1–23.

[ece33467-bib-0009] Cameron, S. L. , & Whiting, M. F. (2008). The complete mitochondrial genome of the tobacco hornworm, *Manduca sexta*, (Insecta: Lepidoptera: Sphingidae), and an examination of mitochondrial gene variability within butterflies and moths. Gene, 408, 112–123. 18065166 10.1016/j.gene.2007.10.023

[ece33467-bib-0010] Choi, Y. (2014). Formation of ports and maritime networks in East Sea rim from the standpoint of port institution and strategy. Journal of the Institute for East Asian Studies, Sogang University, 66, 47–90. [in Korean].

[ece33467-bib-0011] Chung, C. H. , Lee, H. J. , Lim, H. S. , & Kim, C. B. (2005). Palynological study of the Late Quaternary sediments at Piseo‐ri, Muan, Korea. Journal of the Korean Earth Science Society, 26, 597–602. [in Korean].

[ece33467-bib-0012] Dakin, E. E. , & Avise, J. C. (2004). Microsatellite null alleles in parentage analysis. Heredity, 93, 504–509. 15292911 10.1038/sj.hdy.6800545

[ece33467-bib-0013] Damm, D. L. , Armstrong, J. B. , Arjo, W. M. , & Piaggio, A. J. (2015). Assessment of population structure of coyotes in east‐central Alabama using microsatellite DNA. Southeastern Naturalist, 14, 106–122.

[ece33467-bib-0014] deWaard, J. R. , Mitchell, A. , Keena, M. A. , Gopurenko, D. , Boykin, L. M. , Armstrong, K. F. , … Humble, L. M. (2010). Towards a global barcode library for *Lymantria* (Lepidoptera: Lymantriinae) tussock moths of biosecurity concern. PLoS One, 5, e14280. 21151562 10.1371/journal.pone.0014280 PMC3000334

[ece33467-bib-0015] Earl, D. A. , & von Holdt, B. M. (2012). STRUCTURE HARVESTER: A website and program for visualizing STRUCTURE output and implementing the Evanno method. Conservation Genetic Resources, 4, 359–361.

[ece33467-bib-0016] Evanno, G. , Regnaut, S. , & Goudet, J. (2005). Detecting the number of clusters of individuals using the software STRUCTURE: A simulation study. Molecular Ecology, 14, 2611–2620. 15969739 10.1111/j.1365-294X.2005.02553.x

[ece33467-bib-0017] Excoffier, L. , Laval, G. , & Schneider, S. (2005). Arlequin ver. 3.0: An integrated software package for population genetics data analysis. Evolutionary Bioinformatics Online, 1, 47–50. PMC2658868 19325852

[ece33467-bib-0018] Falush, D. , Stephens, M. , & Pritchard, J. K. (2003). Inference of population structure using multilocus genotype data: Linked loci and correlated allele frequencies. Genetics, 164, 1567–1587. 12930761 10.1093/genetics/164.4.1567 PMC1462648

[ece33467-bib-0019] Folmer, O. , Black, M. , Hoeh, W. , Lutz, R. , & Vrijenhock, R. (1994). DNA primers for amplification of mitochondrial cytochrome c oxidase subunit I from diverse metazoan invertebrates. Molecular Marine Biology and Biotechnology, 3, 294–299. 7881515

[ece33467-bib-0020] Fu, Y. X. (1997). Statistical test of neutrality of mutations against population growth, hitchhiking and background selection. Genetics, 147, 915–925. 9335623 10.1093/genetics/147.2.915 PMC1208208

[ece33467-bib-0021] Gao, M. , Hou, G. , & Guo, F. (2016). Conceptual model of underground brine formation in the silty coast of Laizhou Bay, Bohai Sea, China. Journal of Coastal Research, 74, 157–165.

[ece33467-bib-0022] George, C. (1984). Allozyme variation in natural populations of *Lymantria dispar* (Lepidoptera). Genetics Selection Evolution, 16, 1–14. 10.1186/1297-9686-16-1-1 PMC2714342 22879146

[ece33467-bib-0023] Goldschmidt, R. (1934). Lymantria. Bibliographia Genetica, 11, 1–185.

[ece33467-bib-0024] Goldschmidt, R. (1940). The material basis of evolution. New Haven, CT: Yale University Press.

[ece33467-bib-0025] Hajibabaei, M. , Singer, G. A. C. , Hebert, P. D. N. , & Hickey, D. A. (2007). DNA barcoding: How it complements taxonomy, molecular phylogenetics and population genetics. TRENDS in Genetics, 23, 167–172. https://doi.org/10.1916/j.tig.2007.02.001 17316886 10.1016/j.tig.2007.02.001

[ece33467-bib-0026] Han, Y. S. , & Meng, G. L. (1996). Quaternary underground brine in the coastal areas of Northern China. Beijing: Science Press.

[ece33467-bib-0027] Harpending, H. (1994). Signature of ancient population growth in a low resolution mitochondrial DNA mismatch distribution. Human Biology, 66, 591–600. 8088750

[ece33467-bib-0028] Hebert, P. D. N. , Cywinska, A. , Ball, S. L. , & deWaard, J. R. (2003). Biological identification through DNA barcodes. Proceedings of the Royal Society B: Biological Sciences, 270, 313–321. 12614582 10.1098/rspb.2002.2218 PMC1691236

[ece33467-bib-0029] Hess, J. E. , Swalla, B. J. , & Moran, P. (2008). New molecular markers to genetically differentiate populations of *Didemnum vexilluman* (Kott, 2002)—An invasive ascidian species. Aquatic Invasions, 4, 299–310.

[ece33467-bib-0030] Higashiura, Y. , Yamaguchi, H. , Ishihara, M. , Ono, N. , Tsukagoshi, H. , Yokobori, S. , … Fukatsu, T. (2011). Male death resulting from hybridization between subspecies of the gypsy moth, *Lymantria dispar* . Heredity, 106, 603–613. 20628417 10.1038/hdy.2010.92 PMC3183894

[ece33467-bib-0031] Hunter, M. E. , & Hart, K. M. (2013). Rapid microsatellite marker development using next generation pyrosequencing to inform invasive Burmese Python—*Python molurus bivittatus—*management. International Journal of Molecular Sciences, 14, 4793–4804. 23449030 10.3390/ijms14034793 PMC3634432

[ece33467-bib-0032] Huson, D. H. , & Bryant, D. (2006). Application of phylogenetic networks in evolutionary studies. Molecular Biology and Evolution, 23, 254–267. 16221896 10.1093/molbev/msj030

[ece33467-bib-0033] Inoue, H. (1982). Lymantriidae In Inoue H., Sugi S., Kuroko H., Moriuti S. & Kawabe A. (Eds.), Moths of Japan, Vol. 1, Text (pp. 628–638). Tokyo: Kodansha.

[ece33467-bib-0034] Jensen, J. L. , Bohonak, A. J. , & Kelley, S. T. (2005). Isolation by distance, web service. BMC Genetics, 6, 13 IBD ver. 3.23 http://ibdws.sdsu.edu/ 15760479 10.1186/1471-2156-6-13 PMC1079815

[ece33467-bib-0035] Kang, T. H. , Han, S. H. , & Park, S. J. (2015). Development of seven microsatellite markers using next generation sequencing for the conservation on the Korean population of *Dorcus hopei* (E. Saunders, 1854) (Coleoptera, Lucanidae). International Journal of Molecular Sciences, 16, 21330–21341. 26370965 10.3390/ijms160921330 PMC4613255

[ece33467-bib-0036] Kang, T. H. , Han, S. H. , & Park, S. J. (2016). Development of 12 microsatellite markers in *Dorcus titanus castanicolor* (Motschulsky, 1861) (Lucanidae, Coleoptera) from Korea using next‐generation sequencing. International Journal of Molecular Sciences, 17, 1621. 10.3390/ijms17101621 PMC5085654 27669231

[ece33467-bib-0037] Kang, T. H. , Lee, K. S. , & Lee, H. S. (2015). DNA Barcoding of the Korean *Lymantria* Hübner, 1819 (Lepidoptera: Erebidae: Lymantriinae) for quarantine inspection. Journal of Economic Entomology, 108, 1596–1611. 26470300 10.1093/jee/tov111

[ece33467-bib-0038] Kardakoff, N. (1928). Zur Kenntnis der Lepidopteren des Ussuri‐Gebietcs. Entomologische Mitteilungen, 17, 414–425.

[ece33467-bib-0039] Keena, M. A. , Cȏté, M. J. , Grinberg, P. S. , & Wallner, W. E. (2008). World distribution of female flight and genetic variation in *Lymantria dispar* (Lepidoptera: Lymantriidae). Environmental Entomology, 37, 636–649. 18559169 10.1603/0046-225x(2008)37[636:wdoffa]2.0.co;2

[ece33467-bib-0040] Kim, J. B. (2011). Natural environment of East Asia, History of East Asia I (Natural Environment–International Relationship) (pp. 11–91). Seoul: Northeast Asian History Foundation [in Korean].

[ece33467-bib-0041] Kim, S. H. , Kim, N. C. , Kim, H. C. , & Lee, S. H. (2008). A study on development of coastal transportation system between Mokpo and Jeju based on marine logistics analysis results. Journal of the Korean Society of Marine Environment and Safety, 14, 235–240. [in Korean].

[ece33467-bib-0042] Kim, H. , Kim, M. , Kwon, D. H. , Park, S. , Lee, Y. , Jang, H. , … Jang, Y. (2011). Development and characterization of 15 microsatellite loci from *Lycorma delicatula* (Hemiptera: Fulgoridae). Animal Cells and Systems, 15, 295–300.

[ece33467-bib-0043] King, T. L. , Eackles, M. S. , & Chapman, D. C. (2011). Tools for assessing kinship, population structure, phylogeography, and interspecific hybridization in Asian carps invasive to the Mississippi River, USA: Isolation and characterization of novel tetranucleotide microsatellite DNA loci in silver carp *Hypophthalmichthys molitrix* . Conservation Genetic Resources, 3, 397–401.

[ece33467-bib-0044] Koressaar, T. , & Remm, M. (2007). Enhancements and modifications of primer design program Primer3. Bioinformatics, 23, 1289–1291. 17379693 10.1093/bioinformatics/btm091

[ece33467-bib-0045] Koshio, C. , Tomishima, M. , Shimizu, K. , Kim, H. , & Takenaka, O. (2002). Microsatellites in the gypsy moth, *Lymantria dispar* L. (Lepidoptera: Lymantriidae). Applied Entomology and Zoology, 37, 309–312.

[ece33467-bib-0046] Lee, K. S. , Kang, T. H. , Jeong, J. W. , Ryu, D. P. , & Lee, H. S. (2015). Taxonomic review of the genus *Lymantria* (Lepidoptera: Erebidae: Lymantriinae) in Korea. Entomological Research, 45, 225–234.

[ece33467-bib-0047] Lee, J. H. , Lee, H. P. , Schaefer, P. W. , Fuester, R. W. , Park, J. D. , Lee, B. Y. , & Shin, C. H. (2002). Gypsy moth parasitoid complex at Mt. Halla National Park, Cheju Island, Korea. Entomological News, 113, 103–112.

[ece33467-bib-0048] Leese, F. , Mayer, C. , & Held, C. (2008). Isolation of microsatellites from unknown genomes using known genomes as enrichment templates. Limnology and Oceanography: Methods, 7, 412–426.

[ece33467-bib-0049] Librado, P. , & Rozas, J. (2009). DnaSP v5: A software for comprehensive analysis of DNA polymorphism data. Bioinformatics, 25, 1451–1452. 19346325 10.1093/bioinformatics/btp187

[ece33467-bib-0050] Liebhold, A. , Mastro, V. , & Schaefer, P. W. (1989). Learning from the legacy of Léopold Trouvelot. Bulletin of the Entomological Society of America, 35, 20–22.

[ece33467-bib-0051] Linnaeus, C. (1758). Systema Naturae per Regna Tria Naturae, Secundum Classes, Ordines, Genrea, Species, cum Characteribus, Differentiis, Synonymis, Locis. Tomis I (10th edn). Homiae: Laurentii Slavii.

[ece33467-bib-0052] Liu, K. , & Muse, S. V. (2005). PowerMarker: Integrated analysis environment for genetic marker data. Bioinformatics, 21, 2128–2129. 15705655 10.1093/bioinformatics/bti282

[ece33467-bib-0053] López‐Uribe, M. M. , Santiago, C. K. , Bogdanowicz, S. M. , & Danforth, B. N. (2012). Discovery and characterization of microsatellites for the solitary bee *Colletes inaequalis* using Sanger and 454 pyrosequencing. Apidologie, 44, 163–172.

[ece33467-bib-0054] Lowe, S. , Browne, M. , Boudjelas, S. , & De Poorter, M. (2000). 100 of the world's worst invasive alien species: a selection from the Global Invasive Species Database. Invasive Species Specialist Group (ISSG), Species Survival Commission (SSC), World Conservation Union (IUCN), Gland, Switzerland.

[ece33467-bib-0055] Ma, L. , Yu, H. J. , Wang, S. K. , & Yao, J. (2006). Late quaternary environmental evolution in the Bohai Sea and formation of quaternary subsurface brine. Coastal Engineering, 25, 1–6. [in Chinese].

[ece33467-bib-0056] Mayer, C. , Leese, F. , & Tollrian, R. (2010). Genome‐wide analysis of tandem repeats in *Daphnia pulex* – a comparative approach. BMC Genomics, 11, 277. 20433735 10.1186/1471-2164-11-277 PMC3152781

[ece33467-bib-0057] Mieg, T. H. (1886). Lépidopéres Européens. Le Naturaliste, Paris, 8, 236–237.

[ece33467-bib-0058] Oosterhout, C. V. , Hutchinson, W. F. , Wills, D. P. M. , & Shipley, P. (2004). MICRO‐CHECKER: Software for identifying and correcting genotyping errors in microsatellite data. Molecular Ecology Notes, 4, 535–538.

[ece33467-bib-0059] Park, Y. A. , & Cho, S. K. (1998). Marine geology In The Geological Society of Korea (Ed.), Geology of Korea (pp. 621–696). Seoul: Sigma Press [in Korean].

[ece33467-bib-0060] Park, S. J. , & Son, I. (2008). Discussions on distribution and genesis of mountain ranges in the Korean Peninsula (III): Proposing a new mountain range map. Journal of the Geological Society of Korea, 43, 276–295. [in Korean].

[ece33467-bib-0061] Park, S. C. , Yoo, D. G. , Lee, C. W. , & Lee, E. I. (2000). Last glacial sea‐level changes and paleogeography of the Korea (Tsushima) Strait. Geo‐Marine Letters, 20, 64–71.

[ece33467-bib-0062] Perry, J. C. , & Rowe, L. (2011). Rapid microsatellite development for water striders by next‐generation sequencing. Journal of Heredity, 102, 125–129. 20810468 10.1093/jhered/esq099

[ece33467-bib-0063] Pimentel, D. , Zuniga, R. , & Morrison, D. (2005). Update of the environmental and economic costs associated with alien‐invasive species in the United States. Ecological Economics, 52, 273–288.

[ece33467-bib-0064] Pogue, M. G. , & Schaefer, P. W. (2007). A review of selected species of Lymantria Hübner [1819] including three new species (Lepidoptera: Noctuidae: Lymantriinae). Washington, DC: US Department of Agriculture Forest Health Technology Enterprise Team.

[ece33467-bib-0065] Pritchard, J. K. , Stephens, M. , & Donnelly, P. (2000). Inference of population structure using multilocus genotype data. Genetics, 155, 945–959. 10835412 10.1093/genetics/155.2.945 PMC1461096

[ece33467-bib-0066] Qian, L. , An, Y. , Song, J. , Xu, J. , Ye, J. , Wu, C. , … Hao, D. (2014). COI gene geographic variation of gypsy moth (Lepidoptera: Lymantriidae) and a TaqMan PCR diagnostic assay. DNA Barcodes, 2, 10–16.

[ece33467-bib-0067] Reinig, W. F. (1938). Elimination und selektion. Jena: Gustav Fischer.

[ece33467-bib-0068] Rice, W. R. (1989). Analyzing tables of statistical tests. Evolution, 43, 223–225. 28568501 10.1111/j.1558-5646.1989.tb04220.x

[ece33467-bib-0069] Richardson, M. F. , Stanley, A. M. , & Sherman, C. D. H. (2012). Development of novel microsatellite markers for the invasive Northern Pacific seastar, *Asterias amurensis* . Conservation Genetic Resources, 4, 327–330.

[ece33467-bib-0070] Roger, A. R. , & Harpending, H. (1992). Population growth makes waves in the distribution of pairwise genetic differences. Molecular Biology and Evolution, 9, 552–569. 1316531 10.1093/oxfordjournals.molbev.a040727

[ece33467-bib-0071] Sakai, A. K. , Allendorf, F. W. , Holt, J. S. , Lodge, D. M. , Molofsky, J. , With, K. A. , … Weller, S. G. (2001). The population biology of invasive species. Annual Review of Ecology and Systematics, 32, 305–332.

[ece33467-bib-0072] Santana, Q. C. , Coetzee, M. P. A. , Steenkamp, E. T. , Mlonyeni, O. X. , Hammond, G. N. A. , Wingfield, M. J. , & Wingfield, B. D. (2009). Microsatellite discovery by deep sequencing of enriched genomic libraries. BioTechniques, 46, 217–223. 19317665 10.2144/000113085

[ece33467-bib-0073] Schintlmeister, A. (2004). The taxonomy of the genus *Lymantria* Hübner, [1819] (Lepidoptera: Lymantriidae). Quadrifina, 7, 1–248.

[ece33467-bib-0074] Schuelke, M. (2000). An economic method for the fluorescent labeling of PCR fragments. Nature Biotechnology, 18, 233–234. 10.1038/72708 10657137

[ece33467-bib-0075] Selkoe, K. A. , & Toonen, R. J. (2006). Microsatellites for ecologists: A practical guide to using and evaluating microsatellite markers. Ecology Letters, 9, 615–629. 16643306 10.1111/j.1461-0248.2006.00889.x

[ece33467-bib-0076] Sunnucks, P. (2000). Efficient genetic markers for population biology. Trends Ecology and Evolution, 15, 199–203. 10782134 10.1016/s0169-5347(00)01825-5

[ece33467-bib-0077] Tajima, F. (1989). Statistical methods for testing the neutral mutation hypothesis by DNA polymorphism. Genetics, 123, 585–595. 2513255 10.1093/genetics/123.3.585 PMC1203831

[ece33467-bib-0078] Tamura, K. , Stecher, G. , Peterson, D. , Filipski, A. , & Kumar, S. (2013). MEGA6: Molecular evolutionary genetics analysis version 6.0. Molecular Biology and Evolution, 30, 2725–2729. 24132122 10.1093/molbev/mst197 PMC3840312

[ece33467-bib-0079] Tóth, G. , Gáspári, Z. , & Jurka, J. (2000). Microsatellites in different eukaryotic genomes: Survey and analysis. Genome Research, 10, 967–981. 10899146 10.1101/gr.10.7.967 PMC310925

[ece33467-bib-0080] Trojan, P. (1997). The floristic and faunistic Korean refugium during the last glacial period and its significance in postglacial biota formation. Fragmenta Faunistica, 40, 215–221.

[ece33467-bib-0081] Untergrasser, A. , Cutcutache, I. , Koressaar, T. , Ye, J. , Faircloth, B. C. , Remm, M. , & Rozen, S. G. (2012). Primer3−new capabilities and interfaces. Nucleic Acids Research, 40, e115. 22730293 10.1093/nar/gks596 PMC3424584

[ece33467-bib-0082] Vnukovskij, V. (1926). Nouvelles forms de *Lymantria dispar* L. de la Sibérie et de semiretshje. Revue Russe d'Entomologie, 20, 78–81.

[ece33467-bib-0083] Wu, Y. , Molongoski, J. J. , Winograd, D. F. , Bogdanowicz, S. M. , Louyakis, A. S. , Lance, D. R. , … Harrison, R. G. (2015). Genetic structure, admixture and invasion success in a Holarctic defoliator, the gypsy moth (*Lymantria dispar*, Lepidoptera: Erebidae). Molecular Ecology, 24, 1275–1291. 25655667 10.1111/mec.13103

[ece33467-bib-0084] Yoon, S. O. , & Hwang, S. (2009). The reconstruction of natural environment for the last glacial maximum around Korea and adjacent areas. Journal of the Korean Geomorphological Association, 16, 101–112. [in Korean].

[ece33467-bib-0085] Yu, J. N. , Won, C. , Jun, J. , Lim, Y. W. , & Kwak, M. (2011). Fast and cost‐effective mining of microsatellite markers using NGS Technology: An example of a Korean water deer *Hydropotes inermis argyropus* . PLoS One, 6, e26933. 22069476 10.1371/journal.pone.0026933 PMC3206051

[ece33467-bib-0086] Zane, L. , Bargelloni, L. , & Patarnello, T. (2002). Strategies for microsatellite isolation: A review. Molecular Ecology, 11, 1–16. 11903900 10.1046/j.0962-1083.2001.01418.x

