# Supplementary material for: Genetic structure and demographic history of Lymantria dispar (Linnaeus, 1758) (Lepidoptera: Erebidae) in its area of origin and adjacent areas
Source: Ecol Evol. 2017 Sep 30;7(21):9162–78. doi: 10.1002/ece3.3467 (PMC5677484; doi:10.1002/ece3.3467)
Supplement: Supplementary file 1 [file ECE3-7-9162-s001.docx]

**Supplementary Table 1**

| **Sequence Type** | **No. of Obtained Sequences** | **Average Length of Obtained Sequences** | **Total Base-pairs** |
| --- | --- | --- | --- |
| **Reads** | 15,988,036 | 248.58 | 3,974,358,483 |
| **Contigs** | 718,940 | 511 | 367,397,618 |
