# Supplementary material for: Genetic structure and demographic history of Lymantria dispar (Linnaeus, 1758) (Lepidoptera: Erebidae) in its area of origin and adjacent areas
Source: Ecol Evol. 2017 Sep 30;7(21):9162–78. doi: 10.1002/ece3.3467 (PMC5677484; doi:10.1002/ece3.3467)
Supplement: Supplementary file 2 [file ECE3-7-9162-s002.docx]

**Supplementary Table 2.**

| **Marker** | **Allele** | **Site 1** | **Site 6** | **Site 10** | **Site 12** | **Site 16** | **Site 18** | **Site 22** | **Site 26** | **Site 27** | **Site 28** | **Site 30** | **Site 31** | **Site 33** | **Site 34** | **Site 35** | **Site 36** | **Site 37** | **Site 38** | **Site 39** | **Site 41** |
| --- | --- | --- | --- | --- | --- | --- | --- | --- | --- | --- | --- | --- | --- | --- | --- | --- | --- | --- | --- | --- | --- |
| **39767** | 129 | 0.0000 | 0.0000 | 0.0000 | 0.0000 | 0.0000 | 0.0000 | 0.0263 | 0.0000 | 0.0000 | 0.0000 | 0.0000 | 0.0000 | 0.0000 | 0.0000 | 0.0000 | 0.0000 | 0.0000 | 0.0000 | 0.0000 | 0.0000 |
| **39767** | 139 | 0.0000 | 0.0000 | 0.0000 | 0.0000 | 0.0000 | 0.0000 | 0.0000 | 0.0000 | 0.0000 | 0.0000 | 0.0000 | 0.0000 | 0.0000 | 0.0000 | 0.0000 | 0.0000 | 0.0167 | 0.0000 | 0.0000 | 0.0000 |
| **39767** | 141 | 0.0000 | 0.0000 | 0.0000 | 0.0000 | 0.0000 | 0.0000 | 0.0000 | 0.0000 | 0.0000 | 0.0000 | 0.0000 | 0.0000 | 0.0000 | 0.0000 | 0.0000 | 0.0000 | 0.0000 | 0.0167 | 0.0000 | 0.0000 |
| **39767** | 143 | 0.0000 | 0.0000 | 0.0000 | 0.0000 | 0.0000 | 0.0000 | 0.0000 | 0.0000 | 0.0000 | 0.0000 | 0.0250 | 0.0000 | 0.0167 | 0.0000 | 0.0000 | 0.0167 | 0.0167 | 0.0167 | 0.0000 | 0.0000 |
| **39767** | 145 | 0.0750 | 0.1000 | 0.0750 | 0.0500 | 0.0000 | 0.0250 | 0.0526 | 0.0250 | 0.0000 | 0.0750 | 0.0000 | 0.1000 | 0.0333 | 0.2647 | 0.0000 | 0.0500 | 0.0000 | 0.0167 | 0.0556 | 0.0714 |
| **39767** | 147 | 0.1000 | 0.0500 | 0.0500 | 0.0750 | 0.1000 | 0.0250 | 0.1316 | 0.0500 | 0.1500 | 0.1000 | 0.1500 | 0.1000 | 0.0833 | 0.1176 | 0.0333 | 0.1167 | 0.1000 | 0.0000 | 0.1111 | 0.0714 |
| **39767** | 149 | 0.1500 | 0.2000 | 0.2250 | 0.0500 | 0.1750 | 0.3250 | 0.2895 | 0.1250 | 0.2250 | 0.0750 | 0.1250 | 0.2750 | 0.1333 | 0.2941 | 0.1000 | 0.0333 | 0.0500 | 0.1000 | 0.1667 | 0.2143 |
| **39767** | 151 | 0.2000 | 0.4500 | 0.2500 | 0.4750 | 0.3750 | 0.3750 | 0.2105 | 0.3250 | 0.2000 | 0.4000 | 0.4500 | 0.3500 | 0.4500 | 0.2647 | 0.5167 | 0.4833 | 0.5000 | 0.5333 | 0.2778 | 0.2143 |
| **39767** | 153 | 0.3000 | 0.1250 | 0.2250 | 0.1750 | 0.2250 | 0.1500 | 0.2368 | 0.3500 | 0.2500 | 0.2750 | 0.1500 | 0.1000 | 0.1167 | 0.0588 | 0.1500 | 0.0833 | 0.1667 | 0.2167 | 0.2222 | 0.3571 |
| **39767** | 155 | 0.0750 | 0.0500 | 0.0750 | 0.1250 | 0.0750 | 0.0250 | 0.0263 | 0.0250 | 0.1250 | 0.0500 | 0.0750 | 0.0500 | 0.0833 | 0.0000 | 0.1833 | 0.1833 | 0.1333 | 0.1000 | 0.0278 | 0.0714 |
| **39767** | 157 | 0.0000 | 0.0000 | 0.0000 | 0.0250 | 0.0250 | 0.0000 | 0.0000 | 0.0250 | 0.0500 | 0.0250 | 0.0000 | 0.0000 | 0.0167 | 0.0000 | 0.0000 | 0.0167 | 0.0000 | 0.0000 | 0.0000 | 0.0000 |
| **39767** | 159 | 0.0250 | 0.0250 | 0.0750 | 0.0000 | 0.0000 | 0.0500 | 0.0263 | 0.0500 | 0.0000 | 0.0000 | 0.0250 | 0.0000 | 0.0000 | 0.0000 | 0.0000 | 0.0000 | 0.0000 | 0.0000 | 0.0833 | 0.0000 |
| **39767** | 161 | 0.0000 | 0.0000 | 0.0000 | 0.0000 | 0.0000 | 0.0000 | 0.0000 | 0.0000 | 0.0000 | 0.0000 | 0.0000 | 0.0250 | 0.0333 | 0.0000 | 0.0000 | 0.0000 | 0.0000 | 0.0000 | 0.0278 | 0.0000 |
| **39767** | 163 | 0.0250 | 0.0000 | 0.0000 | 0.0000 | 0.0000 | 0.0000 | 0.0000 | 0.0000 | 0.0000 | 0.0000 | 0.0000 | 0.0000 | 0.0000 | 0.0000 | 0.0000 | 0.0000 | 0.0000 | 0.0000 | 0.0278 | 0.0000 |
| **39767** | 165 | 0.0250 | 0.0000 | 0.0000 | 0.0000 | 0.0250 | 0.0250 | 0.0000 | 0.0000 | 0.0000 | 0.0000 | 0.0000 | 0.0000 | 0.0167 | 0.0000 | 0.0000 | 0.0000 | 0.0000 | 0.0000 | 0.0000 | 0.0000 |
| **39767** | 167 | 0.0000 | 0.0000 | 0.0000 | 0.0250 | 0.0000 | 0.0000 | 0.0000 | 0.0000 | 0.0000 | 0.0000 | 0.0000 | 0.0000 | 0.0167 | 0.0000 | 0.0167 | 0.0167 | 0.0167 | 0.0000 | 0.0000 | 0.0000 |
| **39767** | 175 | 0.0250 | 0.0000 | 0.0000 | 0.0000 | 0.0000 | 0.0000 | 0.0000 | 0.0250 | 0.0000 | 0.0000 | 0.0000 | 0.0000 | 0.0000 | 0.0000 | 0.0000 | 0.0000 | 0.0000 | 0.0000 | 0.0000 | 0.0000 |
| **39767** | 179 | 0.0000 | 0.0000 | 0.0250 | 0.0000 | 0.0000 | 0.0000 | 0.0000 | 0.0000 | 0.0000 | 0.0000 | 0.0000 | 0.0000 | 0.0000 | 0.0000 | 0.0000 | 0.0000 | 0.0000 | 0.0000 | 0.0000 | 0.0000 |
| **58587** | 214 | 0.0000 | 0.0000 | 0.0000 | 0.0000 | 0.0250 | 0.0250 | 0.0000 | 0.0000 | 0.0000 | 0.0000 | 0.0000 | 0.0250 | 0.0000 | 0.0294 | 0.0000 | 0.0000 | 0.0000 | 0.0000 | 0.0000 | 0.0000 |
| **58587** | 217 | 0.0000 | 0.0250 | 0.1750 | 0.0750 | 0.1000 | 0.0000 | 0.0000 | 0.0000 | 0.0000 | 0.0250 | 0.0000 | 0.0000 | 0.0167 | 0.0000 | 0.0167 | 0.0500 | 0.0000 | 0.0333 | 0.0000 | 0.0000 |
| **58587** | 220 | 0.1000 | 0.0250 | 0.0750 | 0.0750 | 0.1000 | 0.0500 | 0.0000 | 0.0000 | 0.0250 | 0.0000 | 0.0833 | 0.2000 | 0.1000 | 0.0294 | 0.0333 | 0.0167 | 0.0167 | 0.0000 | 0.0278 | 0.0000 |
| **58587** | 223 | 0.0000 | 0.1250 | 0.1000 | 0.1750 | 0.0750 | 0.1750 | 0.0000 | 0.0250 | 0.0250 | 0.0000 | 0.0556 | 0.0000 | 0.0167 | 0.0294 | 0.0000 | 0.0167 | 0.0167 | 0.0167 | 0.0000 | 0.2143 |
| **58587** | 225 | 0.0000 | 0.0000 | 0.0000 | 0.0000 | 0.0000 | 0.0000 | 0.0000 | 0.0000 | 0.0000 | 0.0000 | 0.0000 | 0.0000 | 0.0333 | 0.0000 | 0.0000 | 0.0000 | 0.0000 | 0.0000 | 0.0000 | 0.0000 |
| **58587** | 226 | 0.6500 | 0.5500 | 0.6000 | 0.4250 | 0.4250 | 0.5750 | 0.7750 | 0.7750 | 0.8250 | 0.7000 | 0.5556 | 0.5750 | 0.5500 | 0.6765 | 0.7000 | 0.7500 | 0.7333 | 0.7333 | 0.8611 | 0.3571 |
| **58587** | 229 | 0.2000 | 0.2750 | 0.0500 | 0.2500 | 0.2500 | 0.1250 | 0.0750 | 0.1750 | 0.0750 | 0.2750 | 0.3056 | 0.2000 | 0.2000 | 0.2353 | 0.1833 | 0.1500 | 0.2000 | 0.2000 | 0.0833 | 0.3571 |
| **58587** | 232 | 0.0250 | 0.0000 | 0.0000 | 0.0000 | 0.0250 | 0.0500 | 0.1250 | 0.0000 | 0.0250 | 0.0000 | 0.0000 | 0.0000 | 0.0333 | 0.0000 | 0.0500 | 0.0167 | 0.0333 | 0.0167 | 0.0278 | 0.0714 |

**Supplementary Table 2.** Continued.

| **Marker** | **Allele** | **Site 1** | **Site 6** | **Site 10** | **Site 12** | **Site 16** | **Site 18** | **Site 22** | **Site 26** | **Site 27** | **Site 28** | **Site 30** | **Site 31** | **Site 33** | **Site 34** | **Site 35** | **Site 36** | **Site 37** | **Site 38** | **Site 39** | **Site 41** |
| --- | --- | --- | --- | --- | --- | --- | --- | --- | --- | --- | --- | --- | --- | --- | --- | --- | --- | --- | --- | --- | --- |
| **58587** | 235 | 0.0250 | 0.0000 | 0.0000 | 0.0000 | 0.0000 | 0.0000 | 0.0000 | 0.0250 | 0.0250 | 0.0000 | 0.0000 | 0.0000 | 0.0000 | 0.0000 | 0.0000 | 0.0000 | 0.0000 | 0.0000 | 0.0000 | 0.0000 |
| **58587** | 238 | 0.0000 | 0.0000 | 0.0000 | 0.0000 | 0.0000 | 0.0000 | 0.0250 | 0.0000 | 0.0000 | 0.0000 | 0.0000 | 0.0000 | 0.0167 | 0.0000 | 0.0167 | 0.0000 | 0.0000 | 0.0000 | 0.0000 | 0.0000 |
| **58587** | 299 | 0.0000 | 0.0000 | 0.0000 | 0.0000 | 0.0000 | 0.0000 | 0.0000 | 0.0000 | 0.0000 | 0.0000 | 0.0000 | 0.0000 | 0.0333 | 0.0000 | 0.0000 | 0.0000 | 0.0000 | 0.0000 | 0.0000 | 0.0000 |
| **124259** | 184 | 0.0000 | 0.0500 | 0.0000 | 0.0000 | 0.0250 | 0.0000 | 0.0250 | 0.0278 | 0.0000 | 0.0000 | 0.0000 | 0.0000 | 0.0000 | 0.0000 | 0.0000 | 0.0000 | 0.0000 | 0.0000 | 0.0000 | 0.0000 |
| **124259** | 186 | 0.0000 | 0.0250 | 0.0500 | 0.0000 | 0.0000 | 0.0000 | 0.0250 | 0.0000 | 0.0000 | 0.0250 | 0.0250 | 0.0000 | 0.0000 | 0.0000 | 0.0167 | 0.0000 | 0.0345 | 0.0500 | 0.0278 | 0.0000 |
| **124259** | 188 | 0.0000 | 0.0000 | 0.0000 | 0.0250 | 0.0000 | 0.0000 | 0.0000 | 0.0000 | 0.0000 | 0.0000 | 0.0000 | 0.0000 | 0.0000 | 0.0000 | 0.0000 | 0.0000 | 0.0000 | 0.0000 | 0.0000 | 0.0000 |
| **124259** | 190 | 0.0263 | 0.0500 | 0.0500 | 0.0250 | 0.0250 | 0.1500 | 0.0750 | 0.0556 | 0.0500 | 0.1000 | 0.1500 | 0.0500 | 0.0333 | 0.0000 | 0.0667 | 0.0667 | 0.0172 | 0.0500 | 0.0556 | 0.0000 |
| **124259** | 192 | 0.0263 | 0.0000 | 0.0500 | 0.0250 | 0.0250 | 0.0000 | 0.0250 | 0.0000 | 0.0250 | 0.0250 | 0.0000 | 0.0000 | 0.0167 | 0.1563 | 0.0167 | 0.0333 | 0.0000 | 0.0000 | 0.1111 | 0.1667 |
| **124259** | 194 | 0.0526 | 0.0250 | 0.0500 | 0.1250 | 0.0750 | 0.0250 | 0.0000 | 0.0556 | 0.0250 | 0.0250 | 0.0500 | 0.0000 | 0.0333 | 0.0625 | 0.2333 | 0.0667 | 0.1897 | 0.1333 | 0.0278 | 0.1667 |
| **124259** | 196 | 0.1842 | 0.1750 | 0.2250 | 0.2250 | 0.3250 | 0.3500 | 0.3750 | 0.2778 | 0.2250 | 0.1250 | 0.3500 | 0.5000 | 0.1667 | 0.2500 | 0.3000 | 0.3500 | 0.2414 | 0.2833 | 0.1944 | 0.2500 |
| **124259** | 198 | 0.3947 | 0.3250 | 0.4000 | 0.4000 | 0.1000 | 0.3000 | 0.2750 | 0.3889 | 0.3500 | 0.3500 | 0.1250 | 0.3250 | 0.4000 | 0.3125 | 0.2167 | 0.2167 | 0.2586 | 0.1833 | 0.3056 | 0.2500 |
| **124259** | 200 | 0.1842 | 0.3000 | 0.1250 | 0.1250 | 0.3250 | 0.1500 | 0.1500 | 0.1944 | 0.2250 | 0.2500 | 0.1750 | 0.0750 | 0.2167 | 0.1250 | 0.1500 | 0.2167 | 0.2586 | 0.2833 | 0.1667 | 0.0833 |
| **124259** | 202 | 0.0263 | 0.0250 | 0.0000 | 0.0250 | 0.0000 | 0.0000 | 0.0000 | 0.0000 | 0.0250 | 0.0250 | 0.0250 | 0.0000 | 0.0333 | 0.0000 | 0.0000 | 0.0167 | 0.0000 | 0.0167 | 0.0000 | 0.0000 |
| **124259** | 204 | 0.0526 | 0.0000 | 0.0250 | 0.0000 | 0.0250 | 0.0000 | 0.0000 | 0.0000 | 0.0250 | 0.0000 | 0.0500 | 0.0000 | 0.0667 | 0.0000 | 0.0000 | 0.0000 | 0.0000 | 0.0000 | 0.0000 | 0.0833 |
| **124259** | 206 | 0.0000 | 0.0000 | 0.0000 | 0.0000 | 0.0500 | 0.0250 | 0.0000 | 0.0000 | 0.0000 | 0.0750 | 0.0000 | 0.0250 | 0.0333 | 0.0000 | 0.0000 | 0.0000 | 0.0000 | 0.0000 | 0.0278 | 0.0000 |
| **124259** | 208 | 0.0000 | 0.0000 | 0.0250 | 0.0250 | 0.0250 | 0.0000 | 0.0250 | 0.0000 | 0.0000 | 0.0000 | 0.0250 | 0.0000 | 0.0000 | 0.0625 | 0.0000 | 0.0000 | 0.0000 | 0.0000 | 0.0556 | 0.0000 |
| **124259** | 210 | 0.0000 | 0.0250 | 0.0000 | 0.0000 | 0.0000 | 0.0000 | 0.0000 | 0.0000 | 0.0500 | 0.0000 | 0.0250 | 0.0000 | 0.0000 | 0.0000 | 0.0000 | 0.0000 | 0.0000 | 0.0000 | 0.0000 | 0.0000 |
| **124259** | 212 | 0.0263 | 0.0000 | 0.0000 | 0.0000 | 0.0000 | 0.0000 | 0.0250 | 0.0000 | 0.0000 | 0.0000 | 0.0000 | 0.0000 | 0.0000 | 0.0313 | 0.0000 | 0.0167 | 0.0000 | 0.0000 | 0.0278 | 0.0000 |
| **124259** | 216 | 0.0000 | 0.0000 | 0.0000 | 0.0000 | 0.0000 | 0.0000 | 0.0000 | 0.0000 | 0.0000 | 0.0000 | 0.0000 | 0.0250 | 0.0000 | 0.0000 | 0.0000 | 0.0000 | 0.0000 | 0.0000 | 0.0000 | 0.0000 |
| **124259** | 218 | 0.0263 | 0.0000 | 0.0000 | 0.0000 | 0.0000 | 0.0000 | 0.0000 | 0.0000 | 0.0000 | 0.0000 | 0.0000 | 0.0000 | 0.0000 | 0.0000 | 0.0000 | 0.0000 | 0.0000 | 0.0000 | 0.0000 | 0.0000 |
| **124259** | 246 | 0.0000 | 0.0000 | 0.0000 | 0.0000 | 0.0000 | 0.0000 | 0.0000 | 0.0000 | 0.0000 | 0.0000 | 0.0000 | 0.0000 | 0.0000 | 0.0000 | 0.0000 | 0.0167 | 0.0000 | 0.0000 | 0.0000 | 0.0000 |
| **134079** | 159 | 0.0000 | 0.0000 | 0.0000 | 0.0000 | 0.0000 | 0.0000 | 0.1579 | 0.0588 | 0.0000 | 0.0000 | 0.0000 | 0.0000 | 0.0000 | 0.0000 | 0.0000 | 0.0000 | 0.0000 | 0.0000 | 0.0000 | 0.0000 |
| **134079** | 189 | 0.0000 | 0.0000 | 0.0000 | 0.0000 | 0.0000 | 0.0000 | 0.0000 | 0.0588 | 0.0000 | 0.0250 | 0.0000 | 0.0263 | 0.0167 | 0.0000 | 0.0000 | 0.0000 | 0.0333 | 0.0333 | 0.0000 | 0.0000 |
| **134079** | 192 | 0.0789 | 0.0000 | 0.0000 | 0.0500 | 0.0250 | 0.0750 | 0.0000 | 0.0000 | 0.0250 | 0.0000 | 0.0000 | 0.0000 | 0.0000 | 0.0000 | 0.0167 | 0.0500 | 0.0667 | 0.0167 | 0.0000 | 0.0000 |
| **134079** | 195 | 0.2632 | 0.1000 | 0.0789 | 0.1750 | 0.2750 | 0.2500 | 0.1053 | 0.1471 | 0.1750 | 0.2250 | 0.1500 | 0.3684 | 0.0667 | 0.0714 | 0.6167 | 0.6000 | 0.5500 | 0.6000 | 0.1944 | 0.1429 |
| **134079** | 198 | 0.3421 | 0.3750 | 0.3947 | 0.3000 | 0.2500 | 0.2250 | 0.3421 | 0.4118 | 0.3500 | 0.3000 | 0.2750 | 0.2368 | 0.4333 | 0.3214 | 0.0333 | 0.0333 | 0.0167 | 0.0500 | 0.3333 | 0.1429 |

**Supplementary Table 2.** Continued.

| **Marker** | **Allele** | **Site 1** | **Site 6** | **Site 10** | **Site 12** | **Site 16** | **Site 18** | **Site 22** | **Site 26** | **Site 27** | **Site 28** | **Site 30** | **Site 31** | **Site 33** | **Site 34** | **Site 35** | **Site 36** | **Site 37** | **Site 38** | **Site 39** | **Site 41** |
| --- | --- | --- | --- | --- | --- | --- | --- | --- | --- | --- | --- | --- | --- | --- | --- | --- | --- | --- | --- | --- | --- |
| **134079** | 201 | 0.0789 | 0.2750 | 0.2105 | 0.2000 | 0.1000 | 0.1500 | 0.2105 | 0.0294 | 0.2750 | 0.2500 | 0.1750 | 0.0000 | 0.2000 | 0.4643 | 0.1000 | 0.0667 | 0.0667 | 0.1167 | 0.4167 | 0.1429 |
| **134079** | 204 | 0.0263 | 0.0250 | 0.1316 | 0.1000 | 0.1500 | 0.1500 | 0.1053 | 0.2059 | 0.1250 | 0.0250 | 0.0750 | 0.1579 | 0.0667 | 0.0357 | 0.1167 | 0.1000 | 0.1167 | 0.0333 | 0.0000 | 0.0714 |
| **134079** | 207 | 0.0526 | 0.1250 | 0.0526 | 0.0250 | 0.1000 | 0.0000 | 0.0526 | 0.0588 | 0.0500 | 0.1000 | 0.1250 | 0.0526 | 0.0667 | 0.0000 | 0.0000 | 0.0000 | 0.0000 | 0.0000 | 0.0278 | 0.2143 |
| **134079** | 210 | 0.0000 | 0.0000 | 0.0263 | 0.0000 | 0.0000 | 0.0500 | 0.0000 | 0.0000 | 0.0000 | 0.0250 | 0.0250 | 0.0000 | 0.0000 | 0.0000 | 0.0167 | 0.0000 | 0.0000 | 0.0000 | 0.0000 | 0.1429 |
| **134079** | 213 | 0.0263 | 0.0250 | 0.0263 | 0.0250 | 0.0500 | 0.0250 | 0.0263 | 0.0000 | 0.0000 | 0.0250 | 0.0500 | 0.0789 | 0.0167 | 0.0000 | 0.0000 | 0.0167 | 0.0000 | 0.0000 | 0.0000 | 0.1429 |
| **134079** | 216 | 0.0789 | 0.0250 | 0.0263 | 0.0250 | 0.0250 | 0.0250 | 0.0000 | 0.0000 | 0.0000 | 0.0000 | 0.0250 | 0.0526 | 0.0833 | 0.0357 | 0.0500 | 0.0667 | 0.1000 | 0.1000 | 0.0000 | 0.0000 |
| **134079** | 219 | 0.0000 | 0.0250 | 0.0263 | 0.0500 | 0.0000 | 0.0500 | 0.0000 | 0.0294 | 0.0000 | 0.0250 | 0.0500 | 0.0000 | 0.0000 | 0.0000 | 0.0333 | 0.0667 | 0.0500 | 0.0500 | 0.0278 | 0.0000 |
| **134079** | 222 | 0.0000 | 0.0250 | 0.0000 | 0.0250 | 0.0250 | 0.0000 | 0.0000 | 0.0000 | 0.0000 | 0.0000 | 0.0250 | 0.0000 | 0.0167 | 0.0714 | 0.0000 | 0.0000 | 0.0000 | 0.0000 | 0.0000 | 0.0000 |
| **134079** | 228 | 0.0263 | 0.0000 | 0.0000 | 0.0000 | 0.0000 | 0.0000 | 0.0000 | 0.0000 | 0.0000 | 0.0000 | 0.0000 | 0.0000 | 0.0000 | 0.0000 | 0.0000 | 0.0000 | 0.0000 | 0.0000 | 0.0000 | 0.0000 |
| **134079** | 234 | 0.0000 | 0.0000 | 0.0000 | 0.0000 | 0.0000 | 0.0000 | 0.0000 | 0.0000 | 0.0000 | 0.0000 | 0.0000 | 0.0000 | 0.0167 | 0.0000 | 0.0000 | 0.0000 | 0.0000 | 0.0000 | 0.0000 | 0.0000 |
| **134079** | 240 | 0.0000 | 0.0000 | 0.0000 | 0.0250 | 0.0000 | 0.0000 | 0.0000 | 0.0000 | 0.0000 | 0.0000 | 0.0250 | 0.0263 | 0.0167 | 0.0000 | 0.0167 | 0.0000 | 0.0000 | 0.0000 | 0.0000 | 0.0000 |
| **134079** | 243 | 0.0000 | 0.0000 | 0.0263 | 0.0000 | 0.0000 | 0.0000 | 0.0000 | 0.0000 | 0.0000 | 0.0000 | 0.0000 | 0.0000 | 0.0000 | 0.0000 | 0.0000 | 0.0000 | 0.0000 | 0.0000 | 0.0000 | 0.0000 |
| **134079** | 270 | 0.0263 | 0.0000 | 0.0000 | 0.0000 | 0.0000 | 0.0000 | 0.0000 | 0.0000 | 0.0000 | 0.0000 | 0.0000 | 0.0000 | 0.0000 | 0.0000 | 0.0000 | 0.0000 | 0.0000 | 0.0000 | 0.0000 | 0.0000 |
| **230995** | 148 | 0.1750 | 0.2895 | 0.1000 | 0.3000 | 0.4500 | 0.1250 | 0.1000 | 0.1500 | 0.2000 | 0.2000 | 0.2500 | 0.2000 | 0.1500 | 0.0000 | 0.0667 | 0.0667 | 0.0833 | 0.1000 | 0.1944 | 0.1667 |
| **230995** | 151 | 0.0250 | 0.0263 | 0.0250 | 0.0000 | 0.0000 | 0.0000 | 0.0000 | 0.0000 | 0.0250 | 0.0250 | 0.0000 | 0.0250 | 0.0333 | 0.0000 | 0.0167 | 0.0167 | 0.0333 | 0.0333 | 0.0556 | 0.0833 |
| **230995** | 154 | 0.2000 | 0.2105 | 0.2500 | 0.2250 | 0.1000 | 0.2750 | 0.2250 | 0.2500 | 0.2000 | 0.3250 | 0.2250 | 0.2000 | 0.3167 | 0.4231 | 0.0167 | 0.0167 | 0.0000 | 0.0000 | 0.1944 | 0.0833 |
| **230995** | 157 | 0.2500 | 0.1316 | 0.2500 | 0.1250 | 0.2000 | 0.1250 | 0.2750 | 0.2000 | 0.1750 | 0.1250 | 0.2000 | 0.0500 | 0.2500 | 0.5769 | 0.3000 | 0.3000 | 0.3000 | 0.3500 | 0.1944 | 0.1667 |
| **230995** | 160 | 0.1250 | 0.0789 | 0.0750 | 0.0250 | 0.0500 | 0.0750 | 0.0250 | 0.0750 | 0.0500 | 0.1000 | 0.0250 | 0.0250 | 0.1000 | 0.0000 | 0.1500 | 0.2167 | 0.2167 | 0.2000 | 0.1389 | 0.0000 |
| **230995** | 162 | 0.0000 | 0.0000 | 0.0000 | 0.0000 | 0.0000 | 0.0000 | 0.0000 | 0.0000 | 0.0000 | 0.0000 | 0.0000 | 0.0000 | 0.0167 | 0.0000 | 0.0000 | 0.0000 | 0.0000 | 0.0000 | 0.0000 | 0.0000 |
| **230995** | 163 | 0.1250 | 0.1579 | 0.1750 | 0.1750 | 0.1250 | 0.1750 | 0.2500 | 0.2500 | 0.2750 | 0.1500 | 0.1750 | 0.1000 | 0.0833 | 0.0000 | 0.3000 | 0.2000 | 0.3000 | 0.1833 | 0.1389 | 0.2500 |
| **230995** | 166 | 0.0000 | 0.0000 | 0.0000 | 0.0250 | 0.0000 | 0.0750 | 0.0500 | 0.0500 | 0.0000 | 0.0000 | 0.0250 | 0.0250 | 0.0000 | 0.0000 | 0.0333 | 0.0000 | 0.0000 | 0.0000 | 0.0278 | 0.0000 |
| **230995** | 169 | 0.0500 | 0.0000 | 0.0250 | 0.0250 | 0.0000 | 0.0000 | 0.0000 | 0.0000 | 0.0000 | 0.0000 | 0.0000 | 0.0000 | 0.0167 | 0.0000 | 0.0833 | 0.0500 | 0.0167 | 0.0167 | 0.0278 | 0.0833 |
| **230995** | 172 | 0.0000 | 0.0000 | 0.0000 | 0.0000 | 0.0000 | 0.0000 | 0.0000 | 0.0000 | 0.0000 | 0.0000 | 0.0000 | 0.0000 | 0.0000 | 0.0000 | 0.0000 | 0.0000 | 0.0167 | 0.0000 | 0.0000 | 0.0833 |
| **230995** | 175 | 0.0250 | 0.0526 | 0.0250 | 0.0000 | 0.0000 | 0.0000 | 0.0250 | 0.0000 | 0.0250 | 0.0250 | 0.0250 | 0.1000 | 0.0000 | 0.0000 | 0.0000 | 0.0500 | 0.0000 | 0.0167 | 0.0000 | 0.0000 |
| **230995** | 178 | 0.0000 | 0.0263 | 0.0000 | 0.0000 | 0.0000 | 0.0000 | 0.0000 | 0.0000 | 0.0000 | 0.0000 | 0.0000 | 0.0000 | 0.0167 | 0.0000 | 0.0000 | 0.0000 | 0.0000 | 0.0000 | 0.0000 | 0.0000 |
| **230995** | 181 | 0.0000 | 0.0000 | 0.0250 | 0.0250 | 0.0500 | 0.0000 | 0.0000 | 0.0000 | 0.0250 | 0.0000 | 0.0250 | 0.0000 | 0.0000 | 0.0000 | 0.0000 | 0.0000 | 0.0167 | 0.0667 | 0.0000 | 0.0000 |

**Supplementary Table 2.** Continued.

| **Marker** | **Allele** | **Site 1** | **Site 6** | **Site 10** | **Site 12** | **Site 16** | **Site 18** | **Site 22** | **Site 26** | **Site 27** | **Site 28** | **Site 30** | **Site 31** | **Site 33** | **Site 34** | **Site 35** | **Site 36** | **Site 37** | **Site 38** | **Site 39** | **Site 41** |
| --- | --- | --- | --- | --- | --- | --- | --- | --- | --- | --- | --- | --- | --- | --- | --- | --- | --- | --- | --- | --- | --- |
| **230995** | 184 | 0.0000 | 0.0263 | 0.0000 | 0.0000 | 0.0250 | 0.0000 | 0.0250 | 0.0000 | 0.0250 | 0.0000 | 0.0250 | 0.0000 | 0.0000 | 0.0000 | 0.0333 | 0.0333 | 0.0167 | 0.0167 | 0.0278 | 0.0000 |
| **230995** | 190 | 0.0250 | 0.0000 | 0.0500 | 0.0500 | 0.0000 | 0.0750 | 0.0000 | 0.0250 | 0.0000 | 0.0250 | 0.0250 | 0.1750 | 0.0167 | 0.0000 | 0.0000 | 0.0500 | 0.0000 | 0.0167 | 0.0000 | 0.0000 |
| **230995** | 193 | 0.0000 | 0.0000 | 0.0000 | 0.0250 | 0.0000 | 0.0500 | 0.0250 | 0.0000 | 0.0000 | 0.0250 | 0.0000 | 0.0000 | 0.0000 | 0.0000 | 0.0000 | 0.0000 | 0.0000 | 0.0000 | 0.0000 | 0.0833 |
| **230995** | 196 | 0.0000 | 0.0000 | 0.0000 | 0.0000 | 0.0000 | 0.0250 | 0.0000 | 0.0000 | 0.0000 | 0.0000 | 0.0000 | 0.1000 | 0.0000 | 0.0000 | 0.0000 | 0.0000 | 0.0000 | 0.0000 | 0.0000 | 0.0000 |
| **297455** | 168 | 0.0000 | 0.0000 | 0.0000 | 0.0000 | 0.0000 | 0.0000 | 0.0000 | 0.0000 | 0.0000 | 0.0000 | 0.0000 | 0.0000 | 0.0000 | 0.0000 | 0.0167 | 0.0167 | 0.0333 | 0.0000 | 0.0000 | 0.0000 |
| **297455** | 170 | 0.1250 | 0.0000 | 0.0000 | 0.0000 | 0.0000 | 0.0000 | 0.0000 | 0.0000 | 0.0000 | 0.0000 | 0.0000 | 0.0250 | 0.0000 | 0.0000 | 0.1500 | 0.0833 | 0.0500 | 0.1167 | 0.1944 | 0.0000 |
| **297455** | 172 | 0.0000 | 0.0000 | 0.0000 | 0.0000 | 0.0000 | 0.0000 | 0.0000 | 0.0000 | 0.0000 | 0.0000 | 0.0000 | 0.0000 | 0.0000 | 0.0000 | 0.0000 | 0.0833 | 0.0500 | 0.0000 | 0.0000 | 0.0714 |
| **297455** | 174 | 0.1250 | 0.0000 | 0.0000 | 0.0250 | 0.0000 | 0.0000 | 0.0250 | 0.0000 | 0.0000 | 0.0000 | 0.0000 | 0.0250 | 0.0333 | 0.0000 | 0.0167 | 0.0333 | 0.0833 | 0.0333 | 0.0000 | 0.0000 |
| **297455** | 180 | 0.0000 | 0.0000 | 0.0000 | 0.0000 | 0.0000 | 0.0000 | 0.0000 | 0.0000 | 0.0000 | 0.0000 | 0.0000 | 0.0000 | 0.0167 | 0.0000 | 0.0000 | 0.0000 | 0.0000 | 0.0000 | 0.0000 | 0.0000 |
| **297455** | 182 | 0.0000 | 0.0000 | 0.0000 | 0.0000 | 0.0000 | 0.0000 | 0.0000 | 0.0000 | 0.0000 | 0.0000 | 0.0000 | 0.0000 | 0.0000 | 0.0000 | 0.0000 | 0.0167 | 0.0000 | 0.0000 | 0.0000 | 0.0000 |
| **297455** | 186 | 0.2500 | 0.1750 | 0.2750 | 0.2250 | 0.2500 | 0.1750 | 0.1250 | 0.1316 | 0.1000 | 0.1500 | 0.2750 | 0.1000 | 0.2667 | 0.0000 | 0.1000 | 0.0667 | 0.0500 | 0.0167 | 0.1944 | 0.0000 |
| **297455** | 188 | 0.1750 | 0.1750 | 0.2500 | 0.2000 | 0.2750 | 0.1500 | 0.2750 | 0.2368 | 0.2500 | 0.1250 | 0.1250 | 0.0500 | 0.1833 | 0.4118 | 0.5000 | 0.4000 | 0.5000 | 0.4833 | 0.1667 | 0.5714 |
| **297455** | 190 | 0.0250 | 0.0000 | 0.0250 | 0.0000 | 0.0500 | 0.0000 | 0.0000 | 0.0000 | 0.0250 | 0.0000 | 0.0000 | 0.0000 | 0.0000 | 0.0000 | 0.0000 | 0.0000 | 0.0000 | 0.0000 | 0.0000 | 0.0000 |
| **297455** | 192 | 0.0750 | 0.1000 | 0.1250 | 0.0750 | 0.1750 | 0.1250 | 0.0750 | 0.0789 | 0.1000 | 0.1500 | 0.2000 | 0.0250 | 0.2833 | 0.2059 | 0.0167 | 0.1000 | 0.0167 | 0.0500 | 0.1944 | 0.0714 |
| **297455** | 194 | 0.0000 | 0.1000 | 0.1000 | 0.0750 | 0.0250 | 0.1750 | 0.1500 | 0.1842 | 0.1500 | 0.1250 | 0.0750 | 0.0250 | 0.0000 | 0.0000 | 0.0000 | 0.0000 | 0.0000 | 0.0000 | 0.0556 | 0.0714 |
| **297455** | 196 | 0.0000 | 0.0250 | 0.0500 | 0.0250 | 0.0500 | 0.0000 | 0.0000 | 0.1053 | 0.0500 | 0.0000 | 0.1000 | 0.0500 | 0.0000 | 0.0000 | 0.0000 | 0.0167 | 0.0167 | 0.0000 | 0.0000 | 0.0000 |
| **297455** | 198 | 0.0000 | 0.1000 | 0.0250 | 0.0750 | 0.0250 | 0.1000 | 0.0250 | 0.0263 | 0.1000 | 0.0250 | 0.0000 | 0.0250 | 0.0500 | 0.0000 | 0.0667 | 0.1333 | 0.0833 | 0.1167 | 0.0278 | 0.0000 |
| **297455** | 200 | 0.0500 | 0.0500 | 0.0250 | 0.1000 | 0.1250 | 0.0750 | 0.0500 | 0.1316 | 0.0750 | 0.1750 | 0.0500 | 0.1000 | 0.0500 | 0.0000 | 0.0333 | 0.0333 | 0.0167 | 0.0167 | 0.0833 | 0.0714 |
| **297455** | 202 | 0.0250 | 0.0000 | 0.0000 | 0.0000 | 0.0000 | 0.0000 | 0.1000 | 0.0000 | 0.0750 | 0.0000 | 0.0500 | 0.0000 | 0.0333 | 0.2353 | 0.0833 | 0.0167 | 0.0500 | 0.0833 | 0.0000 | 0.0000 |
| **297455** | 204 | 0.0250 | 0.0500 | 0.0000 | 0.0250 | 0.0000 | 0.0000 | 0.0000 | 0.0263 | 0.0000 | 0.0500 | 0.0000 | 0.2250 | 0.0167 | 0.0000 | 0.0000 | 0.0000 | 0.0000 | 0.0167 | 0.0556 | 0.0000 |
| **297455** | 206 | 0.0000 | 0.0500 | 0.0000 | 0.0500 | 0.0000 | 0.0250 | 0.0250 | 0.0000 | 0.0250 | 0.0000 | 0.0250 | 0.1250 | 0.0000 | 0.0000 | 0.0167 | 0.0000 | 0.0000 | 0.0333 | 0.0000 | 0.0000 |
| **297455** | 208 | 0.0750 | 0.0250 | 0.0250 | 0.0000 | 0.0000 | 0.0000 | 0.0000 | 0.0263 | 0.0000 | 0.0500 | 0.0000 | 0.0000 | 0.0333 | 0.1471 | 0.0000 | 0.0000 | 0.0167 | 0.0167 | 0.0278 | 0.0000 |
| **297455** | 210 | 0.0000 | 0.0000 | 0.0250 | 0.0000 | 0.0000 | 0.0000 | 0.0250 | 0.0000 | 0.0250 | 0.0000 | 0.0000 | 0.0750 | 0.0000 | 0.0000 | 0.0000 | 0.0000 | 0.0000 | 0.0000 | 0.0000 | 0.0000 |
| **297455** | 212 | 0.0000 | 0.0000 | 0.0000 | 0.0000 | 0.0000 | 0.0000 | 0.0000 | 0.0000 | 0.0000 | 0.0500 | 0.0000 | 0.0000 | 0.0000 | 0.0000 | 0.0000 | 0.0000 | 0.0000 | 0.0167 | 0.0000 | 0.0000 |
| **297455** | 214 | 0.0000 | 0.0000 | 0.0000 | 0.0000 | 0.0000 | 0.0000 | 0.0000 | 0.0000 | 0.0000 | 0.0000 | 0.0000 | 0.0000 | 0.0167 | 0.0000 | 0.0000 | 0.0000 | 0.0000 | 0.0000 | 0.0000 | 0.0000 |
| **297455** | 216 | 0.0000 | 0.0250 | 0.0000 | 0.0000 | 0.0000 | 0.0250 | 0.0000 | 0.0000 | 0.0000 | 0.0000 | 0.0000 | 0.0000 | 0.0000 | 0.0000 | 0.0000 | 0.0000 | 0.0000 | 0.0000 | 0.0000 | 0.0000 |

**Supplementary Table 2.** Continued.

| **Marker** | **Allele** | **Site 1** | **Site 6** | **Site 10** | **Site 12** | **Site 16** | **Site 18** | **Site 22** | **Site 26** | **Site 27** | **Site 28** | **Site 30** | **Site 31** | **Site 33** | **Site 34** | **Site 35** | **Site 36** | **Site 37** | **Site 38** | **Site 39** | **Site 41** |
| --- | --- | --- | --- | --- | --- | --- | --- | --- | --- | --- | --- | --- | --- | --- | --- | --- | --- | --- | --- | --- | --- |
| **297455** | 218 | 0.0000 | 0.0250 | 0.0000 | 0.0000 | 0.0000 | 0.0250 | 0.0000 | 0.0000 | 0.0000 | 0.0000 | 0.0000 | 0.0500 | 0.0167 | 0.0000 | 0.0000 | 0.0000 | 0.0167 | 0.0000 | 0.0000 | 0.0714 |
| **297455** | 220 | 0.0000 | 0.0250 | 0.0250 | 0.0000 | 0.0000 | 0.0000 | 0.0000 | 0.0000 | 0.0000 | 0.0000 | 0.0000 | 0.0000 | 0.0000 | 0.0000 | 0.0000 | 0.0000 | 0.0167 | 0.0000 | 0.0000 | 0.0000 |
| **297455** | 224 | 0.0000 | 0.0250 | 0.0000 | 0.0750 | 0.0000 | 0.0000 | 0.0000 | 0.0000 | 0.0000 | 0.0000 | 0.0000 | 0.0000 | 0.0000 | 0.0000 | 0.0000 | 0.0000 | 0.0000 | 0.0000 | 0.0000 | 0.0000 |
| **297455** | 226 | 0.0000 | 0.0000 | 0.0000 | 0.0000 | 0.0000 | 0.0000 | 0.0000 | 0.0000 | 0.0000 | 0.0000 | 0.0000 | 0.0250 | 0.0000 | 0.0000 | 0.0000 | 0.0000 | 0.0000 | 0.0000 | 0.0000 | 0.0000 |
| **297455** | 228 | 0.0000 | 0.0000 | 0.0000 | 0.0000 | 0.0250 | 0.0250 | 0.0250 | 0.0000 | 0.0000 | 0.0000 | 0.0000 | 0.0500 | 0.0000 | 0.0000 | 0.0000 | 0.0000 | 0.0000 | 0.0000 | 0.0000 | 0.0000 |
| **297455** | 230 | 0.0500 | 0.0000 | 0.0000 | 0.0000 | 0.0000 | 0.0750 | 0.0000 | 0.0000 | 0.0000 | 0.0250 | 0.0000 | 0.0000 | 0.0000 | 0.0000 | 0.0000 | 0.0000 | 0.0000 | 0.0000 | 0.0000 | 0.0000 |
| **297455** | 232 | 0.0000 | 0.0000 | 0.0000 | 0.0000 | 0.0000 | 0.0000 | 0.0000 | 0.0000 | 0.0250 | 0.0000 | 0.0000 | 0.0000 | 0.0000 | 0.0000 | 0.0000 | 0.0000 | 0.0000 | 0.0000 | 0.0000 | 0.0000 |
| **297455** | 234 | 0.0000 | 0.0000 | 0.0000 | 0.0000 | 0.0000 | 0.0000 | 0.0000 | 0.0000 | 0.0000 | 0.0000 | 0.0250 | 0.0000 | 0.0000 | 0.0000 | 0.0000 | 0.0000 | 0.0000 | 0.0000 | 0.0000 | 0.0000 |
| **297455** | 236 | 0.0000 | 0.0000 | 0.0000 | 0.0250 | 0.0000 | 0.0000 | 0.0250 | 0.0000 | 0.0000 | 0.0000 | 0.0250 | 0.0250 | 0.0000 | 0.0000 | 0.0000 | 0.0000 | 0.0000 | 0.0000 | 0.0000 | 0.0000 |
| **297455** | 240 | 0.0000 | 0.0000 | 0.0000 | 0.0250 | 0.0000 | 0.0000 | 0.0500 | 0.0000 | 0.0000 | 0.0250 | 0.0000 | 0.0000 | 0.0000 | 0.0000 | 0.0000 | 0.0000 | 0.0000 | 0.0000 | 0.0000 | 0.0000 |
| **297455** | 242 | 0.0000 | 0.0250 | 0.0250 | 0.0000 | 0.0000 | 0.0250 | 0.0000 | 0.0000 | 0.0000 | 0.0000 | 0.0000 | 0.0000 | 0.0000 | 0.0000 | 0.0000 | 0.0000 | 0.0000 | 0.0000 | 0.0000 | 0.0714 |
| **297455** | 244 | 0.0000 | 0.0000 | 0.0000 | 0.0000 | 0.0000 | 0.0000 | 0.0000 | 0.0263 | 0.0000 | 0.0250 | 0.0500 | 0.0000 | 0.0000 | 0.0000 | 0.0000 | 0.0000 | 0.0000 | 0.0000 | 0.0000 | 0.0000 |
| **297455** | 246 | 0.0000 | 0.0250 | 0.0000 | 0.0000 | 0.0000 | 0.0000 | 0.0000 | 0.0263 | 0.0000 | 0.0000 | 0.0000 | 0.0000 | 0.0000 | 0.0000 | 0.0000 | 0.0000 | 0.0000 | 0.0000 | 0.0000 | 0.0000 |
| **297455** | 250 | 0.0000 | 0.0000 | 0.0000 | 0.0000 | 0.0000 | 0.0000 | 0.0000 | 0.0000 | 0.0000 | 0.0250 | 0.0000 | 0.0000 | 0.0000 | 0.0000 | 0.0000 | 0.0000 | 0.0000 | 0.0000 | 0.0000 | 0.0000 |
| **297455** | 254 | 0.0000 | 0.0000 | 0.0250 | 0.0000 | 0.0000 | 0.0000 | 0.0250 | 0.0000 | 0.0000 | 0.0000 | 0.0000 | 0.0000 | 0.0000 | 0.0000 | 0.0000 | 0.0000 | 0.0000 | 0.0000 | 0.0000 | 0.0000 |
| **344041** | 131 | 0.0263 | 0.0000 | 0.0000 | 0.0000 | 0.0000 | 0.0000 | 0.0000 | 0.0000 | 0.0000 | 0.0000 | 0.0000 | 0.0000 | 0.0000 | 0.0000 | 0.0000 | 0.0000 | 0.0000 | 0.0000 | 0.0000 | 0.0000 |
| **344041** | 151 | 0.0263 | 0.0000 | 0.0000 | 0.0000 | 0.0000 | 0.0000 | 0.0278 | 0.0000 | 0.0000 | 0.0000 | 0.0000 | 0.0250 | 0.0000 | 0.0000 | 0.0000 | 0.0000 | 0.0000 | 0.0000 | 0.0000 | 0.0000 |
| **344041** | 158 | 0.1053 | 0.0000 | 0.0000 | 0.0000 | 0.0000 | 0.0000 | 0.0000 | 0.0000 | 0.0000 | 0.0000 | 0.0000 | 0.0000 | 0.0000 | 0.0000 | 0.0000 | 0.0000 | 0.0000 | 0.0000 | 0.0000 | 0.0000 |
| **344041** | 182 | 0.0000 | 0.0000 | 0.0000 | 0.0000 | 0.0000 | 0.0000 | 0.0000 | 0.0000 | 0.0000 | 0.0000 | 0.0000 | 0.0000 | 0.0000 | 0.0294 | 0.0000 | 0.0000 | 0.0000 | 0.0000 | 0.0000 | 0.0000 |
| **344041** | 215 | 0.0000 | 0.0000 | 0.0000 | 0.0000 | 0.0000 | 0.0000 | 0.0000 | 0.0000 | 0.0000 | 0.0000 | 0.0000 | 0.0000 | 0.0000 | 0.0000 | 0.0167 | 0.0000 | 0.0000 | 0.0000 | 0.0000 | 0.0000 |
| **344041** | 221 | 0.0000 | 0.0000 | 0.0000 | 0.0000 | 0.0000 | 0.0000 | 0.0000 | 0.0000 | 0.0000 | 0.0000 | 0.0000 | 0.0000 | 0.0000 | 0.0000 | 0.0000 | 0.0167 | 0.0500 | 0.0000 | 0.0556 | 0.0714 |
| **344041** | 245 | 0.0000 | 0.0000 | 0.0000 | 0.0000 | 0.0000 | 0.0000 | 0.0000 | 0.0000 | 0.0000 | 0.0000 | 0.0250 | 0.0000 | 0.0000 | 0.0000 | 0.0000 | 0.0000 | 0.0000 | 0.0000 | 0.0000 | 0.0000 |
| **344041** | 251 | 0.3684 | 0.3000 | 0.3750 | 0.3250 | 0.3500 | 0.2500 | 0.0833 | 0.3000 | 0.1500 | 0.3750 | 0.2000 | 0.1250 | 0.4000 | 0.4412 | 0.2500 | 0.2000 | 0.2667 | 0.2000 | 0.3611 | 0.0714 |
| **344041** | 254 | 0.0000 | 0.0000 | 0.0000 | 0.0000 | 0.0000 | 0.0000 | 0.0278 | 0.0000 | 0.0000 | 0.0000 | 0.0000 | 0.0000 | 0.0167 | 0.0000 | 0.0000 | 0.0000 | 0.0000 | 0.0000 | 0.0000 | 0.0000 |
| **344041** | 257 | 0.0526 | 0.0750 | 0.1000 | 0.1500 | 0.1250 | 0.0000 | 0.0556 | 0.0250 | 0.0750 | 0.0500 | 0.0500 | 0.1250 | 0.1500 | 0.0000 | 0.0333 | 0.0167 | 0.0500 | 0.0000 | 0.1667 | 0.0714 |
| **344041** | 260 | 0.0526 | 0.1250 | 0.1000 | 0.0500 | 0.0000 | 0.0500 | 0.0556 | 0.0000 | 0.0000 | 0.0000 | 0.0500 | 0.1000 | 0.0500 | 0.0000 | 0.0333 | 0.1000 | 0.0833 | 0.0667 | 0.0000 | 0.0714 |

**Supplementary Table 2.** Continued.

| **Marker** | **Allele** | **Site 1** | **Site 6** | **Site 10** | **Site 12** | **Site 16** | **Site 18** | **Site 22** | **Site 26** | **Site 27** | **Site 28** | **Site 30** | **Site 31** | **Site 33** | **Site 34** | **Site 35** | **Site 36** | **Site 37** | **Site 38** | **Site 39** | **Site 41** |
| --- | --- | --- | --- | --- | --- | --- | --- | --- | --- | --- | --- | --- | --- | --- | --- | --- | --- | --- | --- | --- | --- |
| **344041** | 263 | 0.0000 | 0.0000 | 0.0000 | 0.0000 | 0.0000 | 0.0000 | 0.0000 | 0.0500 | 0.0250 | 0.0000 | 0.0000 | 0.0250 | 0.0000 | 0.0000 | 0.0167 | 0.0000 | 0.0000 | 0.0000 | 0.0000 | 0.0000 |
| **344041** | 266 | 0.1842 | 0.3000 | 0.3750 | 0.3250 | 0.4000 | 0.4250 | 0.5000 | 0.4000 | 0.5250 | 0.3500 | 0.4000 | 0.4000 | 0.1500 | 0.1176 | 0.4167 | 0.4833 | 0.4167 | 0.5000 | 0.2222 | 0.5000 |
| **344041** | 269 | 0.0263 | 0.0000 | 0.0000 | 0.0000 | 0.0000 | 0.0000 | 0.0556 | 0.0000 | 0.0250 | 0.0500 | 0.0250 | 0.0500 | 0.0333 | 0.0000 | 0.0000 | 0.0000 | 0.0167 | 0.0000 | 0.0278 | 0.0000 |
| **344041** | 272 | 0.0000 | 0.0000 | 0.0000 | 0.0000 | 0.0000 | 0.0000 | 0.0000 | 0.0000 | 0.0000 | 0.0250 | 0.0000 | 0.0000 | 0.0000 | 0.0000 | 0.0000 | 0.0167 | 0.0000 | 0.0000 | 0.0000 | 0.0000 |
| **344041** | 275 | 0.0000 | 0.0000 | 0.0000 | 0.0000 | 0.0000 | 0.0000 | 0.0000 | 0.0000 | 0.0000 | 0.0000 | 0.0000 | 0.0000 | 0.0167 | 0.0000 | 0.0000 | 0.0000 | 0.0000 | 0.0000 | 0.0000 | 0.0000 |
| **344041** | 278 | 0.0000 | 0.0000 | 0.0000 | 0.0500 | 0.0000 | 0.0500 | 0.0278 | 0.0250 | 0.0000 | 0.0500 | 0.0000 | 0.0000 | 0.0000 | 0.0000 | 0.0000 | 0.0000 | 0.0000 | 0.0000 | 0.0000 | 0.0714 |
| **344041** | 279 | 0.0000 | 0.0000 | 0.0000 | 0.0000 | 0.0000 | 0.0000 | 0.0000 | 0.0000 | 0.0000 | 0.0000 | 0.0000 | 0.0000 | 0.0000 | 0.0000 | 0.0000 | 0.0000 | 0.0167 | 0.0000 | 0.0000 | 0.0000 |
| **344041** | 284 | 0.0000 | 0.0000 | 0.0250 | 0.0000 | 0.0000 | 0.0000 | 0.0000 | 0.0000 | 0.0000 | 0.0000 | 0.0250 | 0.0000 | 0.0000 | 0.0000 | 0.0000 | 0.0000 | 0.0000 | 0.0000 | 0.0000 | 0.0000 |
| **344041** | 285 | 0.0000 | 0.0000 | 0.0000 | 0.0000 | 0.0000 | 0.0000 | 0.0000 | 0.0000 | 0.0000 | 0.0000 | 0.0000 | 0.0000 | 0.0000 | 0.0000 | 0.0000 | 0.0000 | 0.0000 | 0.0000 | 0.0278 | 0.0000 |
| **344041** | 287 | 0.0000 | 0.0250 | 0.0000 | 0.0000 | 0.0000 | 0.0000 | 0.0000 | 0.0000 | 0.0000 | 0.0000 | 0.0000 | 0.0000 | 0.0000 | 0.0000 | 0.0000 | 0.0000 | 0.0000 | 0.0000 | 0.0000 | 0.0000 |
| **344041** | 290 | 0.0000 | 0.0000 | 0.0000 | 0.0000 | 0.0000 | 0.0000 | 0.0000 | 0.0000 | 0.0000 | 0.0000 | 0.0000 | 0.0000 | 0.0000 | 0.0294 | 0.0000 | 0.0000 | 0.0000 | 0.0000 | 0.0000 | 0.0000 |
| **344041** | 293 | 0.0000 | 0.0000 | 0.0000 | 0.0000 | 0.0000 | 0.0000 | 0.0000 | 0.0000 | 0.0000 | 0.0000 | 0.0000 | 0.0000 | 0.0000 | 0.0588 | 0.0000 | 0.0000 | 0.0000 | 0.0000 | 0.0000 | 0.0000 |
| **344041** | 312 | 0.0000 | 0.0000 | 0.0000 | 0.0000 | 0.0000 | 0.0000 | 0.0000 | 0.0000 | 0.0000 | 0.0000 | 0.0000 | 0.0000 | 0.0000 | 0.0294 | 0.0000 | 0.0000 | 0.0000 | 0.0000 | 0.0000 | 0.0000 |
| **344041** | 327 | 0.0000 | 0.0000 | 0.0000 | 0.0000 | 0.0000 | 0.0000 | 0.0000 | 0.0000 | 0.0000 | 0.0000 | 0.0000 | 0.0000 | 0.0000 | 0.0000 | 0.2333 | 0.1667 | 0.1000 | 0.2333 | 0.1389 | 0.1429 |
| **344041** | 328 | 0.0000 | 0.0000 | 0.0000 | 0.0000 | 0.0000 | 0.0000 | 0.0000 | 0.0000 | 0.0000 | 0.0000 | 0.0000 | 0.0000 | 0.0167 | 0.0000 | 0.0000 | 0.0000 | 0.0000 | 0.0000 | 0.0000 | 0.0000 |
| **344041** | 329 | 0.1579 | 0.1750 | 0.0250 | 0.1000 | 0.1250 | 0.2250 | 0.1667 | 0.2000 | 0.2000 | 0.1000 | 0.2250 | 0.1500 | 0.1667 | 0.2941 | 0.0000 | 0.0000 | 0.0000 | 0.0000 | 0.0000 | 0.0000 |
| **346977** | 165 | 0.0526 | 0.0263 | 0.0000 | 0.0250 | 0.0000 | 0.0250 | 0.0000 | 0.0000 | 0.0250 | 0.0000 | 0.0278 | 0.0000 | 0.0000 | 0.0000 | 0.0000 | 0.0000 | 0.0000 | 0.0000 | 0.0556 | 0.0000 |
| **346977** | 169 | 0.1316 | 0.1316 | 0.1750 | 0.1000 | 0.0500 | 0.0500 | 0.1750 | 0.0250 | 0.0250 | 0.1250 | 0.0833 | 0.1750 | 0.0833 | 0.0000 | 0.0167 | 0.0167 | 0.0000 | 0.0000 | 0.0556 | 0.0000 |
| **346977** | 173 | 0.3421 | 0.3947 | 0.3250 | 0.4500 | 0.5000 | 0.3500 | 0.2500 | 0.2750 | 0.3250 | 0.4250 | 0.3889 | 0.0250 | 0.4000 | 0.2500 | 0.2000 | 0.1833 | 0.1833 | 0.1500 | 0.5556 | 0.0000 |
| **346977** | 177 | 0.4474 | 0.3947 | 0.4000 | 0.3500 | 0.3750 | 0.4000 | 0.5500 | 0.6000 | 0.6000 | 0.3750 | 0.3889 | 0.7250 | 0.4333 | 0.7500 | 0.5167 | 0.4167 | 0.4333 | 0.5833 | 0.2500 | 0.6429 |
| **346977** | 181 | 0.0000 | 0.0000 | 0.0500 | 0.0000 | 0.0250 | 0.0250 | 0.0000 | 0.0000 | 0.0000 | 0.0000 | 0.0278 | 0.0000 | 0.0167 | 0.0000 | 0.0500 | 0.0833 | 0.0500 | 0.0667 | 0.0000 | 0.0000 |
| **346977** | 185 | 0.0000 | 0.0263 | 0.0000 | 0.0000 | 0.0000 | 0.0000 | 0.0000 | 0.0000 | 0.0000 | 0.0000 | 0.0000 | 0.0000 | 0.0000 | 0.0000 | 0.0500 | 0.0500 | 0.0667 | 0.0333 | 0.0000 | 0.0000 |
| **346977** | 189 | 0.0000 | 0.0263 | 0.0250 | 0.0250 | 0.0250 | 0.0250 | 0.0000 | 0.0250 | 0.0000 | 0.0250 | 0.0278 | 0.0250 | 0.0167 | 0.0000 | 0.1000 | 0.1667 | 0.1500 | 0.1167 | 0.0000 | 0.2857 |
| **346977** | 193 | 0.0263 | 0.0000 | 0.0000 | 0.0500 | 0.0250 | 0.1000 | 0.0250 | 0.0750 | 0.0000 | 0.0500 | 0.0278 | 0.0250 | 0.0333 | 0.0000 | 0.0500 | 0.0667 | 0.0833 | 0.0333 | 0.0833 | 0.0714 |
| **346977** | 197 | 0.0000 | 0.0000 | 0.0250 | 0.0000 | 0.0000 | 0.0250 | 0.0000 | 0.0000 | 0.0250 | 0.0000 | 0.0278 | 0.0250 | 0.0000 | 0.0000 | 0.0167 | 0.0167 | 0.0333 | 0.0167 | 0.0000 | 0.0000 |
| **346977** | 201 | 0.0000 | 0.0000 | 0.0000 | 0.0000 | 0.0000 | 0.0000 | 0.0000 | 0.0000 | 0.0000 | 0.0000 | 0.0000 | 0.0000 | 0.0167 | 0.0000 | 0.0000 | 0.0000 | 0.0000 | 0.0000 | 0.0000 | 0.0000 |
