# Supplementary material for: Genetic structure and demographic history of Lymantria dispar (Linnaeus, 1758) (Lepidoptera: Erebidae) in its area of origin and adjacent areas
Source: Ecol Evol. 2017 Sep 30;7(21):9162–78. doi: 10.1002/ece3.3467 (PMC5677484; doi:10.1002/ece3.3467)
Supplement: Supplementary file 3 [file ECE3-7-9162-s003.docx]

**Supplementary Table 3**

| **MSL** | **RP** | **MAF** | **Gn** | **SS** | **An** | **GD** | **Ho** | **PIC** | **HWE** |
| --- | --- | --- | --- | --- | --- | --- | --- | --- | --- |
| **39767** | **Site 1*** | 0.3000 | 16 | 20 | 10 | 0.8238 | 0.8000 | 0.8031 | 0.8380 |
|  | **Site 6*** | 0.4500 | 11 | 20 | 7 | 0.7263 | 0.7000 | 0.6943 | 0.4000 |
|  | **Site 10** | 0.2500 | 11 | 20 | 8 | 0.8163 | 1.0000 | 0.7916 | 0.1920 |
|  | **Site 12*** | 0.4750 | 12 | 20 | 8 | 0.7163 | 0.6000 | 0.6879 | 0.2160 |
|  | **Site 16*** | 0.3750 | 11 | 20 | 7 | 0.7613 | 0.6500 | 0.7277 | 0.2060 |
|  | **Site 18*** | 0.3750 | 11 | 20 | 8 | 0.7263 | 0.7000 | 0.6828 | 0.8260 |
|  | **Site 22*** | 0.2895 | 13 | 20 | 8 | 0.7936 | 0.5789 | 0.7635 | 0.0550 |
|  | **Site 26*** | 0.3500 | 12 | 20 | 9 | 0.7488 | 0.7000 | 0.7120 | 0.3290 |
|  | **Site 27*** | 0.2500 | 12 | 20 | 6 | 0.8063 | 0.8000 | 0.7775 | 0.2490 |
|  | **Site 28** | 0.4000 | 11 | 20 | 7 | 0.7400 | 0.9000 | 0.7039 | 0.8580 |
|  | **Site 30*** | 0.4500 | 11 | 20 | 7 | 0.7300 | 0.7000 | 0.6994 | 0.3740 |
|  | **Site 31*** | 0.3500 | 13 | 20 | 7 | 0.7688 | 0.6000 | 0.7363 | 0.1850 |
|  | **Site 33*** | 0.4500 | 18 | 30 | 11 | 0.7489 | 0.6667 | 0.7274 | 0.2400 |
|  | **Site 34** | 0.2941 | 5 | 17 | 5 | 0.7561 | 1.0000 | 0.7141 | 0.0050 |
|  | **Site 35** | 0.5167 | 10 | 30 | 6 | 0.6656 | 0.7333 | 0.6267 | 0.4410 |
|  | **Site 36** | 0.4833 | 14 | 30 | 9 | 0.7078 | 0.7333 | 0.6783 | 0.9820 |
|  | **Site 37*** | 0.5000 | 13 | 30 | 8 | 0.6911 | 0.6333 | 0.6594 | 0.3870 |
|  | **Site 38*** | 0.5333 | 12 | 30 | 7 | 0.6478 | 0.6333 | 0.6070 | 0.9340 |
|  | **Site 39*** | 0.2778 | 16 | 18 | 9 | 0.8210 | 0.7778 | 0.7983 | 0.9400 |
|  | **Site 41*** | 0.3571 | 6 | 7 | 6 | 0.7653 | 0.7143 | 0.7308 | 0.3010 |
| **58587** | **Site 1** | 0.6500 | 6 | 20 | 5 | 0.5263 | 0.6000 | 0.4820 | 0.3760 |
|  | **Site 6** | 0.5500 | 5 | 20 | 5 | 0.6050 | 0.9000 | 0.5464 | 0.0330 |
|  | **Site 10** | 0.6000 | 5 | 20 | 5 | 0.5913 | 0.8000 | 0.5548 | 0.5480 |
|  | **Site 12*** | 0.4250 | 9 | 20 | 5 | 0.7150 | 0.6500 | 0.6713 | 0.2770 |
|  | **Site 16** | 0.4250 | 8 | 20 | 7 | 0.7300 | 1.0000 | 0.6939 | 0.0030 |
|  | **Site 18** | 0.5750 | 6 | 20 | 6 | 0.6175 | 0.8500 | 0.5817 | 0.5640 |
|  | **Site 22*** | 0.7750 | 5 | 20 | 4 | 0.3775 | 0.3500 | 0.3510 | 0.5020 |
|  | **Site 26*** | 0.7750 | 5 | 20 | 4 | 0.3675 | 0.3500 | 0.3291 | 0.6910 |
|  | **Site 27*** | 0.8250 | 5 | 20 | 6 | 0.3113 | 0.3000 | 0.3002 | 0.1940 |
|  | **Site 28*** | 0.7000 | 4 | 20 | 3 | 0.4338 | 0.3000 | 0.3589 | 0.2440 |
|  | **Site 30*** | 0.5556 | 7 | 20 | 4 | 0.5880 | 0.4444 | 0.5222 | 0.2000 |
|  | **Site 31*** | 0.5750 | 7 | 20 | 4 | 0.5888 | 0.5500 | 0.5321 | 0.9480 |
|  | **Site 33*** | 0.5500 | 11 | 30 | 9 | 0.6433 | 0.6333 | 0.6093 | 0.0280 |
|  | **Site 34** | 0.6765 | 6 | 17 | 5 | 0.4844 | 0.5294 | 0.4311 | 1.0000 |
|  | **Site 35*** | 0.7000 | 8 | 30 | 6 | 0.4722 | 0.4333 | 0.4349 | 0.7440 |
|  | **Site 36*** | 0.7500 | 8 | 30 | 6 | 0.4117 | 0.3333 | 0.3824 | 0.3900 |
|  | **Site 37** | 0.7333 | 6 | 30 | 5 | 0.4206 | 0.4333 | 0.3756 | 0.4950 |
|  | **Site 38** | 0.7333 | 7 | 30 | 5 | 0.4206 | 0.4333 | 0.3756 | 0.7470 |
|  | **Site 39** | 0.8611 | 4 | 18 | 4 | 0.2500 | 0.2778 | 0.2374 | 1.0000 |
|  | **Site 41** | 0.3571 | 6 | 7 | 4 | 0.6939 | 0.7143 | 0.6348 | 1.0000 |

**Supplementary Table 3.** Continued.

| **MSL** | **RP** | **MAF** | **Gn** | **SS** | **An** | **GD** | **Ho** | **PIC** | **HWE** |
| --- | --- | --- | --- | --- | --- | --- | --- | --- | --- |
| **124259** | **Site 1** | 0.3947 | 11 | 20 | 10 | 0.7673 | 0.7895 | 0.7398 | 0.0420 |
|  | **Site 6*** | 0.3250 | 10 | 20 | 9 | 0.7663 | 0.6500 | 0.7318 | 0.0150 |
|  | **Site 10** | 0.4000 | 13 | 20 | 9 | 0.7625 | 0.8000 | 0.7345 | 0.5190 |
|  | **Site 12*** | 0.4000 | 13 | 20 | 9 | 0.7550 | 0.6500 | 0.7236 | 0.5530 |
|  | **Site 16*** | 0.3250 | 13 | 20 | 10 | 0.7675 | 0.7500 | 0.7359 | 0.5770 |
|  | **Site 18** | 0.3500 | 11 | 20 | 6 | 0.7413 | 0.7500 | 0.6984 | 0.5570 |
|  | **Site 22** | 0.3750 | 12 | 20 | 9 | 0.7525 | 0.8000 | 0.7173 | 0.3870 |
|  | **Site 26*** | 0.3889 | 9 | 20 | 6 | 0.7269 | 0.5556 | 0.6825 | 0.0440 |
|  | **Site 27** | 0.3500 | 12 | 20 | 9 | 0.7688 | 0.8500 | 0.7354 | 0.6350 |
|  | **Site 28*** | 0.3500 | 13 | 20 | 9 | 0.7813 | 0.7500 | 0.7527 | 0.8060 |
|  | **Site 30** | 0.3500 | 14 | 20 | 10 | 0.8013 | 0.9000 | 0.7785 | 0.9590 |
|  | **Site 31** | 0.5000 | 9 | 20 | 6 | 0.6350 | 0.7500 | 0.5755 | 0.9280 |
|  | **Site 33*** | 0.4000 | 16 | 30 | 9 | 0.7561 | 0.6333 | 0.7252 | 0.2980 |
|  | **Site 34*** | 0.3125 | 10 | 17 | 7 | 0.7910 | 0.6250 | 0.7617 | 0.0070 |
|  | **Site 35*** | 0.3000 | 15 | 30 | 7 | 0.7811 | 0.7667 | 0.7470 | 0.9540 |
|  | **Site 36** | 0.3500 | 14 | 30 | 9 | 0.7728 | 0.9333 | 0.7406 | 0.3170 |
|  | **Site 37*** | 0.2586 | 13 | 30 | 6 | 0.7705 | 0.7241 | 0.7315 | 0.6090 |
|  | **Site 38*** | 0.2833 | 14 | 30 | 7 | 0.7828 | 0.7667 | 0.7499 | 0.1860 |
|  | **Site 39** | 0.3056 | 13 | 18 | 10 | 0.8194 | 0.8333 | 0.7979 | 0.4700 |
|  | **Site 41** | 0.2500 | 5 | 7 | 6 | 0.8056 | 1.0000 | 0.7772 | 0.5310 |
| **134079** | **Site 1** | 0.3421 | 12 | 20 | 10 | 0.7895 | 0.8421 | 0.7638 | 0.3520 |
|  | **Site 6** | 0.3750 | 11 | 20 | 9 | 0.7550 | 0.9500 | 0.7208 | 0.1680 |
|  | **Site 10** | 0.3947 | 10 | 20 | 10 | 0.7701 | 0.8421 | 0.7438 | 0.0480 |
|  | **Site 12*** | 0.3000 | 14 | 20 | 11 | 0.8213 | 0.7500 | 0.8001 | 0.2040 |
|  | **Site 16** | 0.2750 | 14 | 20 | 9 | 0.8150 | 0.9000 | 0.7911 | 0.9540 |
|  | **Site 18*** | 0.2500 | 15 | 20 | 9 | 0.8300 | 0.8000 | 0.8086 | 0.5280 |
|  | **Site 22*** | 0.3421 | 11 | 20 | 7 | 0.7881 | 0.6842 | 0.7597 | 0.0510 |
|  | **Site 26*** | 0.4118 | 11 | 20 | 8 | 0.7543 | 0.5294 | 0.7250 | 0.0160 |
|  | **Site 27** | 0.3500 | 11 | 20 | 6 | 0.7525 | 0.9000 | 0.7132 | 0.9670 |
|  | **Site 28** | 0.3000 | 13 | 20 | 9 | 0.7838 | 0.8000 | 0.7517 | 0.5480 |
|  | **Site 30** | 0.2750 | 16 | 20 | 11 | 0.8425 | 0.9500 | 0.8251 | 0.8410 |
|  | **Site 31** | 0.3684 | 12 | 20 | 8 | 0.7701 | 0.7895 | 0.7395 | 0.6670 |
|  | **Site 33** | 0.4333 | 14 | 30 | 11 | 0.7506 | 0.8000 | 0.7253 | 0.2750 |
|  | **Site 34** | 0.4643 | 6 | 17 | 6 | 0.6684 | 0.8571 | 0.6156 | 0.0890 |
|  | **Site 35** | 0.6167 | 11 | 30 | 9 | 0.5906 | 0.6000 | 0.5678 | 0.3340 |
|  | **Site 36*** | 0.6000 | 11 | 30 | 8 | 0.6128 | 0.5667 | 0.5926 | 0.0460 |
|  | **Site 37*** | 0.5500 | 12 | 30 | 8 | 0.6611 | 0.5333 | 0.6381 | 0.0120 |
|  | **Site 38*** | 0.6000 | 10 | 30 | 8 | 0.6089 | 0.5667 | 0.5858 | 0.0270 |
|  | **Site 39** | 0.4167 | 8 | 18 | 5 | 0.6759 | 0.7222 | 0.6148 | 0.8710 |
|  | **Site 41*** | 0.2143 | 7 | 7 | 7 | 0.8469 | 0.7143 | 0.8277 | 0.2440 |

**Supplementary Table 3.** Continued.

| **MSL** | **RP** | **MAF** | **Gn** | **SS** | **An** | **GD** | **Ho** | **PIC** | **HWE** |
| --- | --- | --- | --- | --- | --- | --- | --- | --- | --- |
| **230995** | **Site 1** | 0.2500 | 13 | 20 | 9 | 0.8313 | 0.8500 | 0.8097 | 0.0400 |
|  | **Site 6*** | 0.2895 | 14 | 20 | 9 | 0.8186 | 0.7368 | 0.7956 | 0.3440 |
|  | **Site 10** | 0.2500 | 15 | 20 | 10 | 0.8238 | 0.9000 | 0.8016 | 0.9390 |
|  | **Site 12*** | 0.3000 | 15 | 20 | 10 | 0.8075 | 0.8000 | 0.7823 | 0.6910 |
|  | **Site 16*** | 0.4500 | 11 | 20 | 7 | 0.7263 | 0.7000 | 0.6943 | 0.4270 |
|  | **Site 18** | 0.2750 | 17 | 20 | 9 | 0.8425 | 0.8500 | 0.8249 | 0.8150 |
|  | **Site 22** | 0.2750 | 15 | 20 | 9 | 0.7963 | 0.8000 | 0.7670 | 0.8510 |
|  | **Site 26*** | 0.2500 | 12 | 20 | 7 | 0.8038 | 0.6500 | 0.7752 | 0.0880 |
|  | **Site 27** | 0.2750 | 15 | 20 | 9 | 0.8088 | 0.8500 | 0.7820 | 0.9960 |
|  | **Site 28*** | 0.3250 | 12 | 20 | 9 | 0.8038 | 0.5500 | 0.7788 | 0.0010 |
|  | **Site 30*** | 0.2500 | 15 | 20 | 10 | 0.8125 | 0.7500 | 0.7864 | 0.8000 |
|  | **Site 31** | 0.2000 | 16 | 20 | 10 | 0.8550 | 0.9000 | 0.8384 | 0.7100 |
|  | **Site 33*** | 0.3167 | 18 | 30 | 10 | 0.7956 | 0.6333 | 0.7684 | 0.1680 |
|  | **Site 34** | 0.5769 | 2 | 17 | 2 | 0.4882 | 0.8462 | 0.3690 | 0.0260 |
|  | **Site 35** | 0.3000 | 16 | 30 | 9 | 0.7833 | 0.8333 | 0.7532 | 0.8950 |
|  | **Site 36** | 0.3000 | 19 | 30 | 10 | 0.8094 | 0.8333 | 0.7851 | 0.9400 |
|  | **Site 37** | 0.3000 | 15 | 30 | 9 | 0.7639 | 0.8000 | 0.7266 | 0.8410 |
|  | **Site 38** | 0.3500 | 17 | 30 | 10 | 0.7872 | 0.8000 | 0.7598 | 0.6010 |
|  | **Site 39*** | 0.1944 | 12 | 18 | 9 | 0.8426 | 0.8333 | 0.8229 | 0.1170 |
|  | **Site 41*** | 0.2500 | 6 | 7 | 8 | 0.8472 | 0.8333 | 0.8296 | 0.5120 |
| **297455** | **Site 1** | 0.2500 | 13 | 20 | 11 | 0.8575 | 0.9500 | 0.8426 | 0.0260 |
|  | **Site 6** | 0.1750 | 17 | 20 | 16 | 0.8963 | 0.9000 | 0.8877 | 0.2650 |
|  | **Site 10** | 0.2750 | 14 | 20 | 13 | 0.8288 | 0.9500 | 0.8094 | 0.4550 |
|  | **Site 12*** | 0.2250 | 17 | 20 | 13 | 0.8713 | 0.8000 | 0.8591 | 0.1430 |
|  | **Site 16*** | 0.2750 | 11 | 20 | 9 | 0.8088 | 0.8000 | 0.7830 | 0.0480 |
|  | **Site 18** | 0.1750 | 16 | 20 | 12 | 0.8763 | 0.9000 | 0.8637 | 0.2990 |
|  | **Site 22*** | 0.2750 | 16 | 20 | 14 | 0.8613 | 0.8000 | 0.8486 | 0.1430 |
|  | **Site 26*** | 0.2368 | 16 | 20 | 11 | 0.8546 | 0.7368 | 0.8385 | 0.1590 |
|  | **Site 27*** | 0.2500 | 17 | 20 | 12 | 0.8688 | 0.8500 | 0.8563 | 0.4910 |
|  | **Site 28*** | 0.1750 | 18 | 20 | 13 | 0.8825 | 0.8500 | 0.8712 | 0.5270 |
|  | **Site 30*** | 0.2750 | 15 | 20 | 11 | 0.8438 | 0.8000 | 0.8271 | 0.2280 |
|  | **Site 31** | 0.2250 | 19 | 20 | 16 | 0.8938 | 0.9000 | 0.8855 | 0.7760 |
|  | **Site 33*** | 0.2833 | 17 | 30 | 12 | 0.8056 | 0.7667 | 0.7804 | 0.1270 |
|  | **Site 34** | 0.4118 | 7 | 17 | 4 | 0.7111 | 0.7647 | 0.6617 | 0.0120 |
|  | **Site 35** | 0.5000 | 12 | 30 | 10 | 0.7039 | 0.9000 | 0.6794 | 0.7640 |
|  | **Site 36** | 0.4000 | 18 | 30 | 12 | 0.7906 | 0.8667 | 0.7728 | 0.8750 |
|  | **Site 37** | 0.5000 | 14 | 30 | 14 | 0.7233 | 0.9000 | 0.7094 | 0.8310 |
|  | **Site 38** | 0.4833 | 13 | 30 | 12 | 0.7261 | 0.8667 | 0.7061 | 0.4080 |
|  | **Site 39** | 0.1944 | 14 | 18 | 9 | 0.8441 | 1.0000 | 0.8250 | 0.9500 |
|  | **Site 41** | 0.5714 | 6 | 7 | 7 | 0.6429 | 0.7143 | 0.6221 | 0.8470 |

**Supplementary Table 3.** Continued.

| **MSL** | **RP** | **MAF** | **Gn** | **SS** | **An** | **GD** | **Ho** | **PIC** | **HWE** |
| --- | --- | --- | --- | --- | --- | --- | --- | --- | --- |
| **344041** | **Site 1** | 0.3684 | 9 | 20 | 9 | 0.7867 | 0.8947 | 0.7615 | 0.0070 |
|  | **Site 6** | 0.3000 | 9 | 20 | 6 | 0.7675 | 0.9000 | 0.7309 | 0.1790 |
|  | **Site 10** | 0.3750 | 10 | 20 | 6 | 0.6975 | 0.8500 | 0.6457 | 0.7690 |
|  | **Site 12** | 0.3250 | 10 | 20 | 6 | 0.7513 | 0.9500 | 0.7123 | 0.1210 |
|  | **Site 16** | 0.4000 | 6 | 20 | 4 | 0.6863 | 0.9000 | 0.6289 | 0.0470 |
|  | **Site 18** | 0.4250 | 8 | 20 | 5 | 0.7013 | 0.9000 | 0.6511 | 0.6770 |
|  | **Site 22** | 0.5000 | 11 | 20 | 9 | 0.7037 | 0.8333 | 0.6793 | 0.9540 |
|  | **Site 26** | 0.4000 | 8 | 20 | 6 | 0.7063 | 0.9500 | 0.6553 | 0.1050 |
|  | **Site 27** | 0.5250 | 6 | 20 | 6 | 0.6550 | 0.9500 | 0.6141 | 0.0690 |
|  | **Site 28** | 0.3750 | 8 | 20 | 7 | 0.7188 | 1.0000 | 0.6745 | 0.0080 |
|  | **Site 30** | 0.4000 | 8 | 20 | 8 | 0.7425 | 0.9500 | 0.7060 | 0.0180 |
|  | **Site 31** | 0.4000 | 11 | 20 | 8 | 0.7725 | 1.0000 | 0.7474 | 0.8680 |
|  | **Site 33** | 0.4000 | 14 | 30 | 9 | 0.7628 | 0.8667 | 0.7339 | 0.2230 |
|  | **Site 34** | 0.4412 | 8 | 17 | 7 | 0.6990 | 0.8824 | 0.6539 | 0.2070 |
|  | **Site 35** | 0.4167 | 8 | 30 | 7 | 0.7067 | 0.9667 | 0.6576 | 0.0090 |
|  | **Site 36** | 0.4833 | 8 | 30 | 7 | 0.6878 | 0.9667 | 0.6473 | 0.0050 |
|  | **Site 37** | 0.4167 | 13 | 30 | 8 | 0.7328 | 0.9000 | 0.6967 | 0.8520 |
|  | **Site 38** | 0.5000 | 5 | 30 | 4 | 0.6511 | 0.9000 | 0.5965 | 0.0540 |
|  | **Site 39** | 0.3611 | 8 | 18 | 7 | 0.7685 | 1.0000 | 0.7355 | 0.4670 |
|  | **Site 41** | 0.5000 | 6 | 7 | 7 | 0.7041 | 1.0000 | 0.6796 | 1.0000 |
| **346977** | **Site 1** | 0.4474 | 7 | 20 | 5 | 0.6620 | 0.6842 | 0.6019 | 0.1380 |
|  | **Site 6*** | 0.3947 | 9 | 20 | 6 | 0.6690 | 0.5789 | 0.6083 | 0.6470 |
|  | **Site 10** | 0.4000 | 9 | 20 | 6 | 0.7000 | 0.7500 | 0.6477 | 0.4700 |
|  | **Site 12*** | 0.4500 | 9 | 20 | 6 | 0.6613 | 0.6500 | 0.6026 | 0.2060 |
|  | **Site 16** | 0.5000 | 8 | 20 | 6 | 0.6050 | 0.6500 | 0.5313 | 0.8400 |
|  | **Site 18*** | 0.4000 | 10 | 20 | 8 | 0.7025 | 0.6000 | 0.6547 | 0.1990 |
|  | **Site 22*** | 0.5500 | 7 | 20 | 4 | 0.6038 | 0.3500 | 0.5431 | 0.0300 |
|  | **Site 26*** | 0.6000 | 6 | 20 | 5 | 0.5575 | 0.4000 | 0.4970 | 0.0080 |
|  | **Site 27*** | 0.6000 | 6 | 20 | 5 | 0.5325 | 0.5000 | 0.4547 | 0.4110 |
|  | **Site 28*** | 0.4250 | 8 | 20 | 5 | 0.6600 | 0.5500 | 0.5971 | 0.1960 |
|  | **Site 30** | 0.3889 | 9 | 20 | 8 | 0.6867 | 0.7222 | 0.6344 | 0.0810 |
|  | **Site 31*** | 0.7250 | 7 | 20 | 6 | 0.4413 | 0.3000 | 0.4063 | 0.1170 |
|  | **Site 33** | 0.4333 | 10 | 30 | 7 | 0.6433 | 0.6667 | 0.5770 | 1.0000 |
|  | **Site 34** | 0.7500 | 3 | 17 | 2 | 0.3750 | 0.3750 | 0.3047 | 1.0000 |
|  | **Site 35*** | 0.5167 | 13 | 30 | 8 | 0.6750 | 0.5333 | 0.6424 | 0.2520 |
|  | **Site 36*** | 0.4167 | 16 | 30 | 8 | 0.7506 | 0.6333 | 0.7204 | 0.3150 |
|  | **Site 37*** | 0.4333 | 15 | 30 | 7 | 0.7411 | 0.7333 | 0.7111 | 0.8460 |
|  | **Site 38** | 0.5833 | 8 | 30 | 7 | 0.6167 | 0.7667 | 0.5862 | 0.9640 |
|  | **Site 39** | 0.5556 | 8 | 18 | 5 | 0.6157 | 0.6667 | 0.5673 | 0.6530 |
|  | **Site 41** | 0.6429 | 3 | 7 | 3 | 0.5000 | 0.7143 | 0.4275 | 1.0000 |

**Supplementary Table 3.** Continued.

| **MSL** | **RP** | **MAF** | **Gn** | **SS** | **An** | **GD** | **Ho** | **PIC** | **HWE** |
| --- | --- | --- | --- | --- | --- | --- | --- | --- | --- |
| **Mean** | **Site 1** | 0.3753 | 10.8750 | 20 | 8.6250 | 0.7555 | 0.8013 | 0.7255 | - |
|  | **Site 6** | 0.3574 | 10.7500 | 20 | 8.3750 | 0.7505 | 0.7895 | 0.7145 | - |
|  | **Site 10** | 0.3681 | 10.8750 | 20 | 8.3750 | 0.7488 | 0.8615 | 0.7162 | - |
|  | **Site 12** | 0.3625 | 12.3750 | 20 | 8.5000 | 0.7623 | 0.7313 | 0.7299 | - |
|  | **Site 16** | 0.3781 | 10.2500 | 20 | 7.3750 | 0.7375 | 0.7938 | 0.6982 | - |
|  | **Site 18** | 0.3531 | 11.7500 | 20 | 7.8750 | 0.7547 | 0.7938 | 0.7207 | - |
|  | **Site 22*** | 0.4227 | 11.2500 | 20 | 8.0000 | 0.7096 | 0.6496 | 0.6787 | - |
|  | **Site 26*** | 0.4266 | 9.8750 | 20 | 7.0000 | 0.6899 | 0.6090 | 0.6518 | - |
|  | **Site 27** | 0.4281 | 10.5000 | 20 | 7.3750 | 0.6880 | 0.7500 | 0.6542 | - |
|  | **Site 28*** | 0.3813 | 10.8750 | 20 | 7.7500 | 0.7255 | 0.7125 | 0.6861 | - |
|  | **Site 30** | 0.3681 | 11.8750 | 20 | 8.6250 | 0.7559 | 0.7771 | 0.7224 | - |
|  | **Site 31** | 0.4179 | 11.7500 | 20 | 8.1250 | 0.7156 | 0.7237 | 0.6826 | - |
|  | **Site 33*** | 0.4083 | 14.7500 | 30 | 9.7500 | 0.7383 | 0.7083 | 0.7059 | - |
|  | **Site 34** | 0.4909 | 5.8750 | 17 | 4.7500 | 0.6216 | 0.7350 | 0.5640 | - |
|  | **Site 35** | 0.4833 | 11.6250 | 30 | 7.7500 | 0.6723 | 0.7208 | 0.6386 | - |
|  | **Site 36** | 0.4729 | 13.5000 | 30 | 8.6250 | 0.6929 | 0.7333 | 0.6650 | - |
|  | **Site 37** | 0.4615 | 12.6250 | 30 | 8.1250 | 0.6881 | 0.7072 | 0.6560 | - |
|  | **Site 38** | 0.5083 | 10.7500 | 30 | 7.5000 | 0.6551 | 0.7167 | 0.6209 | - |
|  | **Site 39** | 0.3958 | 10.3750 | 18 | 7.2500 | 0.7047 | 0.7639 | 0.6749 | - |
|  | **Site 41** | 0.3929 | 5.6250 | 7 | 6.0000 | 0.7257 | 0.8006 | 0.6912 | - |

MSL, Microsatellite loci; RP, Regional population; MAF, Major allele frequency; Gn, Genotype no.; SS, Sample size; An, Allele no.; GD, Gene diversity; Ho, Observed heterozygosity; PIC, Polymorphism information content; HWE, Hardy-Weinberg equilibrium; *, gene diversity>observed heterozygosity; Bonferroni correction adjusted *P*-value=0.0003.
