# Supplementary material for: Genetic structure and demographic history of Lymantria dispar (Linnaeus, 1758) (Lepidoptera: Erebidae) in its area of origin and adjacent areas
Source: Ecol Evol. 2017 Sep 30;7(21):9162–78. doi: 10.1002/ece3.3467 (PMC5677484; doi:10.1002/ece3.3467)
Supplement: Supplementary file 4 [file ECE3-7-9162-s004.docx]

**Supplementary Table 4.**

| **NL** | **13** | **23** | **38** | **79** | **86** | **90** | **99** | **145** | **181** | **196** | **211** | **214** | **217** | **220** | **268** | **271** | **317** | **318** | **343** | **370** | **389** | **454** | **484** | **500** |
| --- | --- | --- | --- | --- | --- | --- | --- | --- | --- | --- | --- | --- | --- | --- | --- | --- | --- | --- | --- | --- | --- | --- | --- | --- |
| **H 01** | T | A | G | G | G | C | G | C | A | G | A | A | A | C | A | C | G | C | T | A | G | C | G | T |
| **H 02** | · | · | · | · | · | · | · | · | · | · | · | · | · | · | · | · | · | · | · | · | · | · | · | · |
| **H 03** | · | · | · | · | · | · | · | · | · | A | · | · | · | · | · | · | · | · | · | · | · | · | · | · |
| **H 04** | · | · | · | · | · | · | · | · | · | A | · | · | · | · | · | · | · | · | · | · | · | · | · | · |
| **H 05** | · | · | · | · | · | · | · | · | · | · | · | · | · | · | · | · | · | · | · | · | · | · | · | · |
| **H 06** | · | · | · | · | · | · | · | · | · | · | · | · | · | · | · | · | · | · | · | · | · | · | · | · |
| **H 07** | · | · | · | · | · | · | · | · | G | · | · | · | · | · | · | · | · | · | · | · | · | · | · | · |
| **H 08** | · | · | · | · | · | · | · | · | · | · | · | · | · | · | · | · | · | · | · | · | · | · | · | · |
| **H 09** | · | · | · | · | · | · | · | · | · | · | · | · | · | · | · | · | · | · | · | · | · | · | · | · |
| **H 10** | · | · | · | A | · | · | · | · | · | A | · | · | · | · | · | · | · | · | · | · | · | · | · | · |
| **H 11** | · | · | · | · | · | · | · | · | · | A | · | · | · | · | G | · | · | · | · | · | · | · | · | · |
| **H 12** | · | · | · | · | · | · | · | · | · | · | · | · | · | · | · | · | · | · | · | · | · | · | · | · |
| **H 13** | · | · | · | · | · | · | · | · | · | · | · | · | · | · | · | · | · | · | · | · | · | · | · | · |
| **H 14** | · | · | · | · | A | · | · | · | · | · | · | · | · | · | · | · | · | · | · | · | · | · | · | · |
| **H 15** | · | · | · | · | · | · | · | · | · | · | · | · | · | · | · | · | · | · | · | · | · | · | · | · |
| **H 16** | · | · | · | · | · | · | · | · | · | · | · | · | G | · | · | · | · | · | · | · | · | · | · | · |
| **H 17** | · | · | · | · | · | · | · | · | · | · | · | · | · | · | · | · | · | · | · | · | · | · | · | · |
| **H 18** | · | · | · | · | · | · | · | · | · | · | · | · | · | · | · | · | · | · | · | · | · | · | · | · |
| **H 19** | · | · | · | · | · | · | · | · | · | · | · | · | · | · | · | · | · | · | · | · | · | · | · | · |
| **H 20** | · | · | · | · | · | · | · | · | · | · | · | · | · | · | · | · | · | · | · | · | · | · | · | · |
| **H 21** | · | · | · | · | · | · | · | · | · | · | · | · | · | T | · | · | · | · | · | · | · | · | · | · |
| **H 22** | · | · | · | · | · | · | · | · | · | · | · | · | · | · | · | · | · | · | · | · | · | · | · | · |
| **H 23** | · | · | · | · | · | · | · | · | · | · | · | · | · | · | · | · | A | · | · | · | · | · | · | · |
| **H 24** | · | · | · | · | · | · | · | · | · | A | · | · | · | · | · | · | · | · | · | · | · | · | · | · |
| **H 25** | · | · | · | A | · | · | · | · | · | · | · | · | · | · | · | · | · | · | · | · | · | · | · | · |

**Supplementary Table 4.** Continued.

| **NL** | **13** | **23** | **38** | **79** | **86** | **90** | **99** | **145** | **181** | **196** | **211** | **214** | **217** | **220** | **268** | **271** | **317** | **318** | **343** | **370** | **389** | **454** | **484** | **500** |
| --- | --- | --- | --- | --- | --- | --- | --- | --- | --- | --- | --- | --- | --- | --- | --- | --- | --- | --- | --- | --- | --- | --- | --- | --- |
| **H 26** | · | · | · | · | · | · | · | · | · | · | · | · | · | · | · | · | · | · | · | · | · | · | · | · |
| **H 27** | · | · | · | · | · | · | · | · | · | · | · | · | · | · | · | · | · | · | C | · | · | · | · | · |
| **H 28** | · | · | · | · | · | · | · | · | · | · | · | · | · | · | · | · | · | · | · | · | · | · | · | · |
| **H 29** | · | · | · | · | · | · | · | · | · | · | · | · | · | · | · | · | · | · | · | · | · | · | · | · |
| **H 30** | · | · | · | · | · | · | · | · | · | A | · | · | · | · | · | · | · | · | · | · | · | · | · | C |
| **H 31** | · | · | · | · | · | · | · | · | · | A | · | · | · | · | · | · | · | · | · | · | · | · | · | · |
| **H 32** | · | · | · | · | · | · | · | · | · | · | · | · | · | · | · | · | · | · | · | · | · | · | · | · |
| **H 33** | · | · | · | · | · | · | · | T | · | · | · | · | · | · | · | · | · | · | · | · | · | · | · | · |
| **H 34** | · | · | · | · | · | · | · | · | · | · | · | · | · | · | · | · | · | · | · | · | · | · | · | · |
| **H 35** | · | · | · | · | · | · | · | · | · | · | · | · | · | · | · | · | · | · | · | · | A | · | · | · |
| **H 36** | · | · | · | · | · | · | · | · | · | A | · | · | · | · | · | · | · | · | C | · | · | · | · | · |
| **H 37** | · | · | · | · | · | · | · | · | · | · | · | · | · | · | · | · | · | · | · | · | A | · | · | · |
| **H 38** | · | · | · | · | · | · | · | · | · | · | · | · | · | · | · | · | · | · | · | · | · | · | · | · |
| **H 39** | · | · | · | · | · | · | · | · | · | · | · | · | · | · | G | · | · | · | · | · | · | · | · | · |
| **H 40** | · | · | · | · | · | · | · | · | · | · | · | · | · | · | G | · | · | · | · | · | · | · | · | · |
| **H 41** | · | · | · | · | · | · | · | · | · | · | · | · | · | · | · | · | · | · | · | · | · | · | · | · |
| **H 42** | · | · | · | · | · | · | · | · | · | · | · | · | · | · | · | · | · | · | · | · | · | · | · | · |
| **H 43** | · | · | · | · | · | · | · | · | · | · | · | · | · | · | · | · | · | · | · | · | · | · | · | · |
| **H 44** | · | · | · | · | · | · | · | · | · | · | G | · | · | · | · | · | · | · | · | · | · | · | · | · |
| **H 45** | · | · | · | · | · | · | · | · | · | · | · | · | · | · | · | · | · | · | · | · | · | · | · | · |
| **H 46** | · | · | · | · | · | · | · | · | · | · | · | · | · | · | · | · | · | · | · | · | A | · | · | · |
| **H 47** | · | · | · | · | · | · | · | · | · | · | · | · | · | · | · | · | · | · | · | · | A | · | · | · |
| **H 48** | · | · | · | · | · | · | · | · | · | · | · | · | · | · | · | · | · | · | · | · | A | · | · | · |
| **H 49** | · | · | · | · | · | · | A | · | · | · | · | · | · | · | · | · | · | · | · | · | · | · | · | · |
| **H 50** | · | · | · | · | · | · | · | · | · | · | · | · | · | · | · | · | · | · | · | · | A | · | · | · |

**Supplementary Table 4.** Continued.

| **NL** | **13** | **23** | **38** | **79** | **86** | **90** | **99** | **145** | **181** | **196** | **211** | **214** | **217** | **220** | **268** | **271** | **317** | **318** | **343** | **370** | **389** | **454** | **484** | **500** |
| --- | --- | --- | --- | --- | --- | --- | --- | --- | --- | --- | --- | --- | --- | --- | --- | --- | --- | --- | --- | --- | --- | --- | --- | --- |
| **H 51** | · | · | · | · | · | · | · | · | · | · | · | · | · | · | · | · | · | · | · | · | · | · | · | · |
| **H 52** | · | · | · | · | · | · | · | · | · | · | · | · | · | · | · | · | · | · | · | · | A | T | · | · |
| **H 53** | · | · | · | · | · | · | · | · | · | · | · | · | · | · | · | · | · | · | · | · | · | · | · | · |
| **H 54** | · | · | · | · | · | · | · | · | · | A | · | · | · | · | · | · | · | · | · | · | A | · | · | · |
| **H 55** | · | · | · | · | · | T | · | · | · | · | · | · | · | · | · | · | · | · | · | · | · | · | · | · |
| **H 56** | · | · | · | · | · | · | · | · | · | · | · | · | · | · | · | · | · | · | · | · | A | · | · | · |
| **H 57** | · | · | · | · | · | · | · | · | · | · | · | · | · | · | · | · | · | · | · | · | A | · | · | · |
| **H 58** | · | · | · | · | · | · | · | · | · | · | · | · | · | · | · | · | · | · | · | · | A | · | · | · |
| **H 59** | · | · | · | · | · | · | · | · | · | · | · | · | · | · | · | · | · | · | · | · | · | · | · | · |
| **H 60** | · | · | · | · | · | · | · | · | · | · | · | · | · | · | · | · | · | · | · | · | A | · | · | · |
| **H 61** | · | · | · | · | · | · | · | · | · | · | · | · | · | · | · | · | · | · | · | · | A | · | · | · |
| **H 62** | · | · | · | · | · | · | · | · | · | · | · | · | · | · | · | · | · | · | · | · | · | · | · | · |
| **H 63** | · | · | · | · | · | · | · | · | · | · | · | · | · | · | · | · | · | · | · | · | A | · | A | · |
| **H 64** | · | · | · | · | · | · | · | · | · | · | · | · | · | · | · | · | · | · | · | · | A | · | · | · |
| **H 65** | · | · | · | · | · | · | · | · | · | · | · | · | · | · | · | · | · | · | · | · | · | · | · | · |
| **H 66** | · | · | · | · | · | · | · | · | · | · | · | · | · | · | · | · | · | · | · | · | · | · | · | · |
| **H 67** | · | · | · | · | · | · | · | · | · | · | · | G | · | · | · | · | · | · | · | · | · | · | · | · |
| **H 68** | · | · | · | · | · | · | · | · | · | · | · | · | · | · | · | · | · | T | · | · | · | · | · | · |
| **H 69** | · | · | · | · | · | · | · | · | · | · | · | · | · | · | · | · | · | · | · | · | · | · | · | · |
| **H 70** | · | · | · | · | · | · | · | · | · | · | · | · | · | · | · | · | · | · | · | · | · | · | · | · |
| **H 71** | C | · | · | · | · | · | · | · | · | · | · | · | · | · | · | · | · | · | · | · | · | · | · | · |
| **H 72** | · | · | · | · | · | · | · | · | · | · | · | · | · | · | · | · | · | · | · | · | A | · | · | · |
| **H 73** | C | · | · | · | · | · | · | · | · | · | · | · | · | · | · | · | · | · | · | · | A | · | · | · |
| **H 74** | · | · | · | · | · | · | · | · | · | · | · | · | · | · | · | · | · | · | · | · | · | · | · | · |
| **H 75** | · | · | · | · | · | · | · | · | · | · | · | · | · | · | · | · | · | · | · | · | · | · | · | · |

**Supplementary Table 4.** Continued.

| **NL** | **13** | **23** | **38** | **79** | **86** | **90** | **99** | **145** | **181** | **196** | **211** | **214** | **217** | **220** | **268** | **271** | **317** | **318** | **343** | **370** | **389** | **454** | **484** | **500** |
| --- | --- | --- | --- | --- | --- | --- | --- | --- | --- | --- | --- | --- | --- | --- | --- | --- | --- | --- | --- | --- | --- | --- | --- | --- |
| **H 76** | · | · | · | · | · | · | · | · | · | · | · | · | · | · | · | · | · | · | · | · | · | · | · | · |
| **H 77** | · | · | · | · | · | · | · | · | · | · | · | · | · | · | · | · | · | · | · | · | · | · | · | · |
| **H 78** | · | · | · | · | · | · | · | · | · | · | · | · | · | · | G | · | · | · | · | · | A | · | · | · |
| **H 79** | · | · | · | · | · | · | · | · | · | · | · | · | · | · | · | · | · | · | · | · | · | · | · | · |
| **H 80** | · | · | · | · | · | · | · | · | · | · | · | · | · | · | · | · | · | · | · | · | A | · | · | · |
| **H 81** | · | · | · | · | · | · | · | · | · | · | · | · | · | · | · | · | · | · | · | G | · | · | · | · |
| **H 82** | · | · | · | · | · | · | · | · | · | · | · | · | · | · | · | T | · | · | · | · | · | · | · | · |
| **H 83** | · | G | · | · | · | · | · | · | · | · | · | · | · | · | · | · | · | · | · | · | · | · | · | · |
| **H 84** | · | · | · | · | · | · | · | · | · | · | · | · | · | · | · | · | · | · | · | · | · | · | · | · |
| **H 85** | · | · | · | · | · | · | · | · | · | · | · | · | · | · | · | · | · | · | · | · | · | · | · | · |
| **H 86** | · | · | · | · | · | · | · | · | · | A | · | · | · | · | · | · | · | · | · | · | · | · | · | · |
| **H 87** | · | · | · | · | · | · | · | · | · | · | · | · | · | · | · | · | · | · | · | · | · | · | · | · |
| **H 88** | · | · | · | · | · | · | · | · | · | · | · | · | · | · | · | · | · | · | · | · | · | · | · | · |
| **H 89** | · | · | · | · | · | · | · | · | · | · | · | · | · | · | · | · | · | · | · | · | · | · | · | · |
| **H 90** | · | · | · | · | · | · | · | · | · | · | · | · | · | · | · | · | · | · | · | · | · | · | · | · |
| **H 91** | · | · | · | · | · | · | · | · | · | · | · | · | · | · | · | · | · | · | · | · | · | · | · | · |
| **H 92** | · | · | · | · | A | · | · | · | · | · | · | · | · | · | · | · | · | · | · | · | · | · | · | · |
| **H 93** | · | · | A | · | · | · | · | · | · | · | · | · | · | · | · | · | · | · | · | · | · | · | · | · |
| **H 94** | · | · | · | · | · | · | · | · | · | · | · | · | · | · | · | · | A | · | · | · | · | · | · | · |
| **H 95** | · | · | · | · | · | · | · | · | · | · | · | · | · | · | · | · | · | · | · | · | · | · | · | · |
| **H 96** | · | · | · | · | · | · | · | · | · | · | · | · | · | · | · | · | · | · | · | · | · | · | · | · |
| **H 97** | · | · | · | · | · | · | · | · | · | · | · | · | · | · | · | · | · | · | · | · | · | · | · | · |
| **H 98** | · | · | · | · | · | · | · | · | · | · | · | · | · | · | · | · | · | · | · | · | · | · | · | · |

**Supplementary Table 4.** Continued.

| **NL** | **506** | **520** | **539** | **541** | **601** | **607** | **616** | **622** | **625** | **628** | **631** | **646** | **723** | **729** | **731** | **744** | **747** | **860** | **909** | **957** | **958** | **959** | **971** | **987** |
| --- | --- | --- | --- | --- | --- | --- | --- | --- | --- | --- | --- | --- | --- | --- | --- | --- | --- | --- | --- | --- | --- | --- | --- | --- |
| **H 01** | G | A | C | T | T | C | C | A | A | A | G | C | C | T | T | C | A | C | T | T | A | A | T | A |
| **H 02** | · | · | · | · | · | · | · | · | · | · | · | · | · | · | · | · | · | · | · | · | · | · | · | · |
| **H 03** | · | · | · | · | · | · | · | · | · | · | · | · | · | · | · | · | · | · | · | · | · | · | · | · |
| **H 04** | · | · | · | · | · | · | · | · | · | · | · | · | · | · | · | · | · | · | · | · | · | · | · | · |
| **H 05** | · | · | · | · | · | · | · | · | · | · | · | · | · | · | · | · | · | · | · | · | · | · | · | · |
| **H 06** | · | · | · | · | · | · | · | · | · | · | · | · | · | · | · | · | · | · | · | · | · | · | · | · |
| **H 07** | · | · | · | · | · | · | · | · | · | · | · | · | · | · | · | · | · | · | · | · | · | · | · | · |
| **H 08** | · | · | · | · | · | · | · | · | · | · | · | · | · | C | · | · | · | · | · | · | · | · | · | · |
| **H 09** | · | · | · | · | · | · | · | · | · | · | · | · | · | · | · | · | · | · | · | · | · | · | · | · |
| **H 10** | · | · | · | · | · | · | · | · | · | · | · | · | · | · | · | · | · | · | · | · | · | · | · | · |
| **H 11** | · | · | · | · | · | · | · | · | · | · | · | · | · | · | · | · | · | · | · | · | · | · | · | · |
| **H 12** | · | G | · | · | · | · | · | G | · | · | · | · | · | · | · | · | · | · | · | · | · | · | · | · |
| **H 13** | · | · | · | · | · | · | · | · | · | · | · | · | T | · | · | · | · | · | · | · | · | · | · | · |
| **H 14** | · | · | · | · | · | · | · | · | · | · | · | · | · | · | · | · | · | · | · | · | · | · | · | · |
| **H 15** | · | · | · | · | · | · | · | · | · | G | · | · | · | · | · | · | · | · | · | · | · | · | · | · |
| **H 16** | · | · | · | · | · | · | · | · | · | · | · | · | · | · | · | · | · | · | · | · | · | · | · | · |
| **H 17** | · | · | · | · | · | · | · | · | · | G | · | · | · | · | · | · | · | · | · | · | · | · | · | · |
| **H 18** | · | · | · | C | · | · | · | · | · | · | A | · | · | · | · | · | · | · | · | · | · | · | · | · |
| **H 19** | · | · | · | · | · | · | · | · | · | · | · | · | · | · | · | · | · | · | · | · | · | · | · | · |
| **H 20** | · | · | · | · | · | · | · | · | · | · | · | · | · | · | · | · | G | · | · | · | · | · | · | · |
| **H 21** | · | · | · | · | · | · | · | · | · | · | · | · | · | · | · | · | · | · | · | · | · | · | · | · |
| **H 22** | · | · | · | · | · | · | · | · | · | · | · | · | · | · | · | · | · | · | · | A | T | T | · | · |
| **H 23** | · | · | · | · | · | · | · | · | · | · | · | · | · | · | · | · | · | · | · | · | · | · | · | · |
| **H 24** | · | · | · | · | · | · | · | · | · | · | · | · | · | · | · | · | · | · | · | · | · | · | · | · |
| **H 25** | · | · | · | · | · | · | · | · | · | · | · | · | · | · | · | · | · | · | · | · | · | · | · | · |

**Supplementary Table 4.** Continued.

| **NL** | **506** | **520** | **539** | **541** | **601** | **607** | **616** | **622** | **625** | **628** | **631** | **646** | **723** | **729** | **731** | **744** | **747** | **860** | **909** | **957** | **958** | **959** | **971** | **987** |
| --- | --- | --- | --- | --- | --- | --- | --- | --- | --- | --- | --- | --- | --- | --- | --- | --- | --- | --- | --- | --- | --- | --- | --- | --- |
| **H 26** | · | · | · | · | · | · | · | · | · | · | · | · | · | · | · | · | · | · | · | · | · | · | · | · |
| **H 27** | · | · | · | · | · | · | · | · | · | · | · | · | · | · | · | · | · | · | · | · | · | · | · | · |
| **H 28** | · | · | · | · | · | · | · | · | · | · | · | · | · | · | · | · | · | T | · | · | · | · | · | · |
| **H 29** | · | · | · | · | · | · | · | · | · | · | · | · | · | · | · | · | · | · | · | · | · | · | · | · |
| **H 30** | · | · | · | · | · | · | · | · | · | · | · | · | · | · | · | · | · | · | · | · | · | · | · | · |
| **H 31** | · | · | · | · | · | · | · | · | · | · | · | · | · | · | · | · | · | · | · | · | · | · | · | · |
| **H 32** | · | · | · | · | · | · | · | · | · | · | A | · | · | · | · | · | · | · | · | · | · | · | · | · |
| **H 33** | · | · | · | · | · | · | · | · | · | · | · | · | · | · | · | · | · | · | · | · | · | · | · | · |
| **H 34** | · | · | · | · | · | · | · | · | · | · | · | · | · | · | · | · | · | · | · | · | · | · | · | · |
| **H 35** | · | · | · | · | · | · | · | · | · | · | A | · | · | · | · | · | · | · | · | · | · | · | · | · |
| **H 36** | · | · | · | · | · | · | · | · | · | · | · | · | · | · | · | · | · | · | · | · | · | · | · | · |
| **H 37** | · | · | · | · | · | · | · | · | · | · | · | · | · | · | · | · | · | · | · | · | · | · | · | · |
| **H 38** | · | · | · | · | · | · | · | · | · | G | A | · | · | · | · | · | · | · | · | · | · | · | · | · |
| **H 39** | · | · | · | · | · | T | · | · | · | · | · | · | · | · | · | · | · | · | · | · | · | · | · | · |
| **H 40** | · | · | · | · | · | · | · | · | · | · | · | · | · | · | · | · | · | · | · | · | · | · | · | · |
| **H 41** | · | · | · | · | · | · | · | · | · | · | · | · | · | · | · | · | · | · | · | · | · | · | · | · |
| **H 42** | · | · | · | · | · | · | · | · | · | · | · | · | · | · | · | · | · | · | · | · | · | · | · | · |
| **H 43** | · | · | · | · | · | · | · | · | · | · | · | · | · | · | · | · | · | · | · | · | · | · | · | · |
| **H 44** | · | · | · | · | · | · | · | · | · | · | · | · | · | · | · | · | · | · | · | · | · | · | · | · |
| **H 45** | · | · | · | · | · | · | · | · | · | · | · | · | · | · | · | · | · | · | · | · | · | · | · | · |
| **H 46** | · | · | · | · | · | · | · | · | · | · | · | · | · | · | · | · | · | · | · | · | · | · | · | · |
| **H 47** | · | · | · | · | · | · | · | · | · | · | · | · | · | · | · | · | · | · | C | · | · | · | · | · |
| **H 48** | · | · | · | · | · | · | · | · | · | G | · | · | · | · | · | · | · | · | · | · | · | · | · | · |
| **H 49** | · | · | · | · | · | · | · | · | · | · | · | · | · | · | · | · | · | · | · | · | · | · | · | · |
| **H 50** | · | · | · | · | · | · | · | · | · | · | · | · | · | · | · | · | · | · | · | · | · | · | · | · |

**Supplementary Table 4.** Continued.

| **NL** | **506** | **520** | **539** | **541** | **601** | **607** | **616** | **622** | **625** | **628** | **631** | **646** | **723** | **729** | **731** | **744** | **747** | **860** | **909** | **957** | **958** | **959** | **971** | **987** |
| --- | --- | --- | --- | --- | --- | --- | --- | --- | --- | --- | --- | --- | --- | --- | --- | --- | --- | --- | --- | --- | --- | --- | --- | --- |
| **H 51** | · | · | · | · | · | · | · | · | · | · | A | · | · | · | · | · | · | · | · | · | · | · | · | · |
| **H 52** | · | · | · | · | · | · | · | · | · | · | · | · | · | · | · | · | · | · | · | · | · | · | · | · |
| **H 53** | · | · | · | · | · | · | · | · | · | G | · | · | · | · | · | · | · | · | · | · | · | · | · | G |
| **H 54** | · | · | · | · | · | · | · | · | · | · | · | · | · | · | · | · | · | · | · | · | · | · | · | · |
| **H 55** | · | · | · | · | · | · | · | · | · | · | · | · | · | · | · | · | · | · | · | · | · | · | · | · |
| **H 56** | · | · | · | · | · | · | · | · | · | · | · | · | · | · | · | · | · | · | · | · | · | · | · | · |
| **H 57** | · | · | · | · | C | · | · | · | · | · | · | · | · | · | · | · | · | · | · | · | · | · | · | · |
| **H 58** | · | · | · | · | · | · | · | · | · | · | · | · | · | · | · | · | · | · | · | · | · | · | · | · |
| **H 59** | · | · | · | · | · | · | · | · | · | · | · | T | · | · | · | · | · | · | · | · | · | · | · | · |
| **H 60** | · | · | · | · | · | · | · | G | · | · | · | · | · | · | · | · | · | · | · | · | · | · | · | · |
| **H 61** | · | · | · | · | · | · | · | · | G | · | · | · | · | · | · | · | · | · | · | · | · | · | · | · |
| **H 62** | · | · | A | · | · | · | · | · | · | · | · | · | · | · | · | · | · | · | · | · | · | · | · | · |
| **H 63** | · | · | · | · | · | · | · | · | · | · | · | · | · | · | · | · | · | · | · | · | · | · | · | · |
| **H 64** | · | · | · | · | · | · | · | · | · | · | · | · | · | · | · | · | · | · | · | · | · | · | · | · |
| **H 65** | · | · | · | · | · | · | · | · | · | · | · | · | · | · | · | · | · | · | · | · | · | · | · | · |
| **H 66** | · | · | · | · | · | · | · | · | · | · | · | · | · | · | · | T | · | · | · | · | · | · | · | · |
| **H 67** | · | · | · | · | · | · | · | · | · | · | · | · | · | · | · | · | · | · | · | · | · | · | · | · |
| **H 68** | · | · | · | · | · | · | · | · | · | · | · | · | · | · | · | · | · | · | · | · | · | · | · | · |
| **H 69** | A | · | · | · | · | · | · | · | · | · | · | · | · | · | · | · | · | · | · | · | · | · | · | · |
| **H 70** | · | · | · | · | · | · | · | · | · | · | · | · | · | · | · | · | · | · | · | · | · | · | · | · |
| **H 71** | · | · | · | · | · | · | · | · | · | · | · | · | · | · | · | · | G | · | · | · | · | · | · | · |
| **H 72** | · | · | · | · | · | · | T | · | · | · | · | · | · | · | · | · | · | · | · | · | · | · | · | · |
| **H 73** | · | · | · | · | · | · | · | · | · | · | · | · | · | · | · | · | · | · | · | · | · | · | · | · |
| **H 74** | · | · | · | · | · | · | · | · | · | · | · | · | · | · | G | · | · | · | · | · | · | · | · | · |
| **H 75** | · | · | · | · | · | · | · | · | · | · | · | · | · | · | · | · | · | · | · | · | · | · | · | · |

**Supplementary Table 4.** Continued.

| **NL** | **506** | **520** | **539** | **541** | **601** | **607** | **616** | **622** | **625** | **628** | **631** | **646** | **723** | **729** | **731** | **744** | **747** | **860** | **909** | **957** | **958** | **959** | **971** | **987** |
| --- | --- | --- | --- | --- | --- | --- | --- | --- | --- | --- | --- | --- | --- | --- | --- | --- | --- | --- | --- | --- | --- | --- | --- | --- |
| **H 76** | · | · | · | · | · | · | · | · | · | · | · | · | · | · | · | · | G | · | · | · | · | · | · | · |
| **H 77** | · | · | · | · | · | · | · | · | · | · | · | · | · | · | · | · | · | · | · | · | · | · | · | · |
| **H 78** | · | · | · | · | · | · | · | · | · | · | · | · | · | · | · | · | · | · | · | · | · | · | · | · |
| **H 79** | · | · | · | · | · | · | · | · | · | · | · | · | · | · | · | · | · | · | · | · | · | · | · | · |
| **H 80** | · | · | · | · | · | · | · | · | · | · | A | · | · | · | · | · | · | · | · | · | · | · | · | · |
| **H 81** | · | · | · | · | · | · | · | · | · | · | · | · | · | · | · | · | · | · | · | · | · | · | · | · |
| **H 82** | · | · | · | · | · | · | · | · | · | · | · | · | · | · | · | · | · | · | · | · | · | · | · | · |
| **H 83** | · | · | · | · | · | · | · | · | · | · | · | · | · | · | · | · | · | · | · | · | · | · | · | · |
| **H 84** | · | · | · | · | · | · | · | G | · | · | · | · | · | · | · | · | · | · | · | · | · | · | · | · |
| **H 85** | · | · | · | · | · | · | · | · | · | · | · | · | · | · | · | · | · | · | · | · | · | · | · | · |
| **H 86** | · | · | · | · | · | · | · | · | · | · | · | · | · | · | · | · | · | · | · | · | · | · | · | · |
| **H 87** | · | · | · | · | · | · | T | · | · | · | A | · | · | · | · | · | · | · | · | · | · | · | · | · |
| **H 88** | · | · | · | · | · | · | · | · | · | · | · | · | · | · | · | · | · | · | · | · | · | · | · | · |
| **H 89** | · | · | · | · | · | · | · | · | · | · | · | · | · | · | · | · | · | A | · | · | · | · | C | · |
| **H 90** | · | · | · | · | · | · | · | · | · | · | · | · | · | · | · | · | · | · | · | · | · | · | · | · |
| **H 91** | · | · | · | · | · | · | · | · | · | · | · | · | · | · | · | · | · | · | · | · | · | · | · | · |
| **H 92** | · | · | · | · | · | · | · | · | · | · | · | · | · | · | · | · | · | · | · | · | · | · | · | · |
| **H 93** | · | · | · | · | · | · | · | · | · | · | · | · | · | · | · | · | · | · | · | · | · | · | · | · |
| **H 94** | · | · | · | · | · | · | · | · | · | · | · | · | · | · | · | · | · | · | · | · | · | · | · | · |
| **H 95** | · | · | · | · | · | · | · | · | · | · | · | · | · | · | · | · | · | · | · | · | · | · | · | · |
| **H 96** | · | · | · | · | · | · | · | · | · | · | · | · | · | · | · | · | · | · | · | · | · | · | · | · |
| **H 97** | · | · | · | · | · | · | · | · | · | · | · | · | · | · | · | · | · | · | · | · | · | · | · | · |
| **H 98** | · | · | · | · | · | · | · | · | · | · | · | · | · | · | · | · | · | · | · | · | · | · | · | · |

**Supplementary Table 4.** Continued.

| **NL** | **1028** | **1043** | **1077** | **1085** | **1113** | **1131** | **1134** | **1202** | **1263** | **1271** | **1294** | **1311** | **1313** | **1328** | **1335** | **1358** | **1364** | **1403** | **1406** | **1413** | **1439** | **1444** | **1455** | **1466** |
| --- | --- | --- | --- | --- | --- | --- | --- | --- | --- | --- | --- | --- | --- | --- | --- | --- | --- | --- | --- | --- | --- | --- | --- | --- |
| **H 01** | C | C | A | A | C | T | A | C | G | C | G | T | G | T | T | A | T | A | T | G | T | C | A | G |
| **H 02** | · | T | · | · | · | · | · | · | · | · | · | · | · | · | · | · | · | · | · | · | · | · | · | · |
| **H 03** | · | · | · | · | · | · | · | · | · | · | · | · | · | · | · | · | · | · | · | · | · | · | · | · |
| **H 04** | · | · | · | · | · | · | · | · | · | · | · | · | · | · | · | · | · | G | · | · | · | · | · | · |
| **H 05** | · | · | · | · | · | · | · | · | · | · | · | · | · | · | · | · | C | · | · | · | · | · | · | · |
| **H 06** | · | · | · | G | · | · | · | · | · | · | · | · | · | · | · | · | · | · | · | · | · | · | · | · |
| **H 07** | · | · | · | · | · | · | · | · | · | · | · | · | · | · | · | · | · | · | · | · | · | · | · | A |
| **H 08** | · | · | · | · | · | · | · | A | · | · | · | · | · | · | · | · | · | · | · | · | · | · | · | · |
| **H 09** | · | · | · | · | · | · | · | · | · | · | · | · | · | · | · | · | · | · | · | · | · | · | · | A |
| **H 10** | · | · | · | · | · | · | · | · | · | · | · | · | · | · | · | · | · | · | · | · | · | · | · | · |
| **H 11** | · | · | · | · | · | · | · | · | · | · | · | · | · | · | · | · | · | · | · | · | · | · | · | · |
| **H 12** | · | · | · | · | · | · | · | · | · | · | · | · | · | · | · | · | · | · | · | · | · | · | · | · |
| **H 13** | · | · | · | · | · | · | · | · | · | · | · | · | · | · | · | · | · | · | · | · | · | · | · | · |
| **H 14** | · | · | · | · | · | · | · | · | · | · | · | · | · | · | · | · | · | · | · | · | · | · | · | · |
| **H 15** | · | T | · | · | · | · | · | · | · | · | · | · | · | · | · | · | · | · | · | · | · | · | · | · |
| **H 16** | · | · | · | · | · | · | · | · | · | · | · | · | · | · | · | · | · | · | · | · | · | · | · | · |
| **H 17** | · | T | · | · | · | · | · | · | · | · | A | · | · | · | · | · | · | · | · | · | · | · | · | · |
| **H 18** | · | · | · | · | · | · | · | · | · | · | · | · | · | · | · | · | · | · | · | · | · | · | · | · |
| **H 19** | · | · | · | · | · | · | · | · | · | · | · | · | · | · | · | · | · | · | · | · | · | · | · | · |
| **H 20** | · | · | · | · | · | · | · | · | · | · | · | · | · | · | · | · | · | · | · | · | · | · | · | · |
| **H 21** | · | · | · | · | · | · | · | · | · | · | · | · | · | · | · | · | · | · | · | · | · | · | · | · |
| **H 22** | · | · | · | · | · | · | · | · | · | · | · | C | · | · | · | · | · | · | · | · | · | · | · | · |
| **H 23** | · | · | · | · | · | · | · | · | · | · | · | · | · | · | · | · | · | · | · | · | · | · | · | · |
| **H 24** | · | · | · | · | · | · | · | · | · | · | · | · | · | · | · | · | · | · | · | · | · | · | · | · |
| **H 25** | · | · | · | · | · | · | · | · | · | · | · | · | · | · | · | · | · | · | · | · | · | · | · | · |

**Supplementary Table 4.** Continued.

| **NL** | **1028** | **1043** | **1077** | **1085** | **1113** | **1131** | **1134** | **1202** | **1263** | **1271** | **1294** | **1311** | **1313** | **1328** | **1335** | **1358** | **1364** | **1403** | **1406** | **1413** | **1439** | **1444** | **1455** | **1466** |
| --- | --- | --- | --- | --- | --- | --- | --- | --- | --- | --- | --- | --- | --- | --- | --- | --- | --- | --- | --- | --- | --- | --- | --- | --- |
| **H 26** | · | · | · | · | · | · | · | · | · | · | · | · | · | · | · | · | · | · | · | · | · | · | · | · |
| **H 27** | · | · | · | · | · | · | · | · | · | · | · | · | · | · | · | · | · | · | · | · | · | · | · | · |
| **H 28** | · | · | · | G | · | · | · | · | · | · | · | · | · | · | · | · | · | · | · | · | · | · | · | · |
| **H 29** | · | · | · | · | · | · | · | · | · | · | · | · | · | · | · | · | · | · | · | · | · | · | · | · |
| **H 30** | · | · | · | · | · | · | · | · | · | · | · | · | · | · | · | · | · | · | · | · | · | · | · | · |
| **H 31** | · | · | · | · | · | · | · | · | · | · | · | · | · | · | · | · | · | · | C | · | · | · | · | · |
| **H 32** | · | · | · | · | · | · | · | · | · | · | · | · | · | · | · | · | · | · | · | · | · | · | G | · |
| **H 33** | · | · | · | · | · | · | · | · | · | · | · | · | · | · | · | · | · | · | · | · | · | · | · | · |
| **H 34** | T | · | · | · | · | · | · | · | · | · | · | · | · | · | · | · | · | · | · | · | · | · | · | · |
| **H 35** | · | · | · | · | · | · | · | · | · | · | · | · | · | · | · | · | · | · | · | · | · | · | · | · |
| **H 36** | · | · | · | · | · | · | · | · | · | · | · | · | · | · | · | · | · | · | · | · | · | · | · | · |
| **H 37** | · | · | · | · | · | · | · | · | · | · | · | · | · | · | · | · | · | · | · | · | · | · | · | · |
| **H 38** | · | T | · | · | · | · | · | · | · | · | · | · | · | · | · | · | · | · | · | · | · | · | · | · |
| **H 39** | · | · | · | · | · | · | · | · | · | · | · | · | · | · | · | · | · | · | · | · | · | · | · | · |
| **H 40** | · | · | · | · | · | · | · | · | · | · | · | · | · | · | · | · | · | · | · | · | · | · | · | · |
| **H 41** | · | · | · | · | T | · | · | · | · | · | · | · | · | · | · | · | · | · | · | · | · | · | · | · |
| **H 42** | · | · | · | · | · | · | · | · | · | · | · | · | · | · | · | · | · | · | · | · | · | · | · | · |
| **H 43** | · | · | · | · | · | · | · | · | · | · | · | · | · | · | A | · | · | · | · | · | · | · | · | · |
| **H 44** | · | · | · | · | · | · | · | · | · | · | · | · | · | · | · | · | · | · | · | · | · | · | · | · |
| **H 45** | · | · | · | · | · | · | · | · | · | · | · | · | · | · | · | · | · | · | · | · | C | · | · | · |
| **H 46** | · | · | · | · | · | · | · | · | · | · | · | · | · | C | · | · | · | · | · | · | · | · | · | · |
| **H 47** | · | · | · | · | · | · | · | · | · | · | · | · | · | · | · | · | · | · | · | · | · | · | · | · |
| **H 48** | · | · | · | · | · | · | · | · | · | · | · | · | · | · | · | · | · | · | · | · | · | · | · | · |
| **H 49** | · | · | · | · | · | · | · | · | · | · | · | · | · | · | · | · | · | · | · | · | · | · | · | · |
| **H 50** | · | · | · | · | · | · | · | · | · | · | · | · | · | · | · | · | · | · | · | · | · | · | · | · |

**Supplementary Table 4.** Continued.

| **NL** | **1028** | **1043** | **1077** | **1085** | **1113** | **1131** | **1134** | **1202** | **1263** | **1271** | **1294** | **1311** | **1313** | **1328** | **1335** | **1358** | **1364** | **1403** | **1406** | **1413** | **1439** | **1444** | **1455** | **1466** |
| --- | --- | --- | --- | --- | --- | --- | --- | --- | --- | --- | --- | --- | --- | --- | --- | --- | --- | --- | --- | --- | --- | --- | --- | --- |
| **H 51** | · | · | · | · | · | · | · | · | · | · | · | · | · | · | · | · | · | · | · | · | · | · | · | · |
| **H 52** | · | · | · | · | · | · | · | · | · | · | · | · | · | · | · | · | · | · | · | A | · | · | · | · |
| **H 53** | · | T | · | · | · | · | · | · | · | · | · | · | · | · | · | · | · | · | · | · | · | · | · | · |
| **H 54** | · | · | · | · | · | · | · | · | · | · | · | · | · | · | · | · | · | · | · | · | · | · | · | · |
| **H 55** | · | · | · | · | · | · | · | · | · | · | · | · | · | · | · | · | · | · | · | · | · | · | · | · |
| **H 56** | · | · | · | · | · | · | · | · | · | · | · | · | · | · | · | · | · | · | · | · | · | · | · | A |
| **H 57** | · | · | · | · | · | · | · | · | · | · | · | · | · | · | · | · | · | · | · | · | · | · | · | · |
| **H 58** | · | · | · | · | · | · | · | · | · | · | · | · | · | · | · | · | · | · | · | · | · | T | · | · |
| **H 59** | · | · | · | · | · | · | · | · | · | · | · | · | · | · | · | · | · | · | · | · | · | · | · | · |
| **H 60** | · | · | · | · | · | · | · | · | · | · | · | · | · | · | · | · | · | · | · | · | · | · | · | · |
| **H 61** | · | · | · | · | · | · | · | · | · | · | · | · | · | · | · | · | · | · | · | · | · | · | · | · |
| **H 62** | · | · | · | · | · | · | · | · | · | · | · | · | · | · | · | · | · | · | · | · | · | · | · | · |
| **H 63** | · | · | · | · | · | · | · | · | · | · | · | · | · | · | · | · | · | · | · | · | · | · | · | · |
| **H 64** | · | · | · | · | · | · | · | · | · | · | · | · | · | · | · | · | · | · | · | · | · | · | · | · |
| **H 65** | · | · | · | · | · | · | · | · | · | · | · | · | · | · | · | · | · | · | · | · | · | · | · | · |
| **H 66** | · | · | · | · | · | · | · | · | · | · | · | · | · | · | · | · | · | · | · | · | · | · | · | · |
| **H 67** | · | · | · | · | · | · | · | · | · | · | · | · | · | · | · | · | · | · | · | · | · | · | · | · |
| **H 68** | · | · | · | · | · | · | · | · | · | · | · | · | · | · | · | · | · | · | · | · | · | · | · | · |
| **H 69** | · | · | · | · | · | · | · | · | · | · | · | · | · | · | · | · | · | · | · | · | · | · | · | A |
| **H 70** | · | · | G | · | · | · | · | · | · | · | · | · | · | · | · | · | · | · | · | · | C | · | · | · |
| **H 71** | · | · | · | · | · | · | · | · | · | · | · | · | · | · | · | · | · | · | · | · | · | · | · | · |
| **H 72** | · | · | · | · | · | · | · | · | · | · | · | · | · | · | · | · | · | · | · | · | · | · | · | · |
| **H 73** | · | · | · | · | · | · | · | · | · | · | · | · | · | · | · | · | · | · | · | · | · | · | · | · |
| **H 74** | · | · | · | · | · | · | · | · | · | · | · | · | · | · | · | · | · | · | · | · | · | · | · | · |
| **H 75** | · | · | · | · | · | · | · | · | · | · | · | · | · | · | · | · | · | · | · | A | · | · | · | · |

**Supplementary Table 4.** Continued.

| **NL** | **1028** | **1043** | **1077** | **1085** | **1113** | **1131** | **1134** | **1202** | **1263** | **1271** | **1294** | **1311** | **1313** | **1328** | **1335** | **1358** | **1364** | **1403** | **1406** | **1413** | **1439** | **1444** | **1455** | **1466** |
| --- | --- | --- | --- | --- | --- | --- | --- | --- | --- | --- | --- | --- | --- | --- | --- | --- | --- | --- | --- | --- | --- | --- | --- | --- |
| **H 76** | · | · | · | · | · | C | · | · | · | · | · | · | · | · | · | · | · | · | · | · | · | · | · | · |
| **H 77** | · | · | · | · | · | · | · | T | · | · | · | · | · | · | · | · | · | · | · | · | · | · | · | · |
| **H 78** | · | · | · | · | · | · | · | · | · | · | · | · | · | · | · | · | · | · | · | · | · | · | · | · |
| **H 79** | · | · | · | · | · | · | · | · | · | · | · | · | · | · | · | · | · | · | · | · | · | · | · | A |
| **H 80** | · | · | · | · | · | · | · | · | · | · | · | · | · | · | · | · | · | · | · | · | · | · | · | · |
| **H 81** | · | · | · | · | · | · | · | · | · | · | · | · | · | · | · | · | · | · | · | · | · | · | · | A |
| **H 82** | · | · | · | · | · | · | · | · | · | · | · | · | · | · | · | · | · | · | · | · | · | · | · | · |
| **H 83** | · | · | · | · | · | · | · | · | · | · | · | · | · | · | · | · | · | · | · | · | · | · | · | · |
| **H 84** | · | · | · | · | · | · | · | · | · | · | · | · | · | · | · | · | · | · | · | · | · | · | · | · |
| **H 85** | · | · | · | · | · | · | · | · | · | · | · | · | · | · | · | · | · | · | · | A | · | · | · | · |
| **H 86** | · | · | · | · | · | · | · | · | · | · | · | · | A | · | · | · | · | · | · | · | · | · | · | · |
| **H 87** | · | · | · | · | · | · | · | · | · | · | · | · | · | · | · | · | · | · | · | · | · | · | · | A |
| **H 88** | · | · | · | · | · | · | · | · | · | · | · | · | · | · | C | · | · | · | · | · | · | · | · | · |
| **H 89** | · | · | · | · | · | · | · | · | A | T | · | · | · | · | · | · | · | · | · | · | · | · | · | · |
| **H 90** | · | · | · | · | · | · | · | · | A | T | · | · | · | · | · | · | · | · | · | · | · | · | · | · |
| **H 91** | · | · | · | · | · | · | · | · | A | · | · | · | · | · | · | G | · | · | · | · | · | · | · | · |
| **H 92** | · | · | · | · | · | · | · | · | A | T | · | · | · | · | · | · | · | · | · | · | · | · | · | · |
| **H 93** | · | · | · | · | · | · | · | · | A | T | · | · | · | · | · | · | · | · | · | · | · | · | · | · |
| **H 94** | · | · | · | · | · | · | · | · | A | T | · | · | · | · | · | · | · | · | · | · | · | · | · | · |
| **H 95** | · | · | · | · | · | · | · | · | A | · | · | · | · | · | · | · | · | · | · | · | · | · | · | · |
| **H 96** | · | · | · | · | · | · | · | · | · | · | · | · | · | · | · | · | · | · | · | · | · | · | · | · |
| **H 97** | · | · | · | · | · | · | · | · | · | · | · | · | · | · | · | · | · | · | · | · | · | · | · | · |
| **H 98** | · | · | · | · | · | · | T | · | · | · | · | · | · | · | · | · | · | · | · | · | · | · | · | · |

**Supplementary Table 4.** Continued.

| **NL** | **1467** | **1472** | **1484** | **1485** | **1490** | **1503** | **1534** | **1535** | **1537** | **1539** | **1541** | **1555** | **1558** |
| --- | --- | --- | --- | --- | --- | --- | --- | --- | --- | --- | --- | --- | --- |
| **H 01** | G | A | A | T | C | G | G | T | C | G | A | A | A |
| **H 02** | · | · | · | · | · | · | · | · | · | · | · | · | · |
| **H 03** | · | · | · | · | · | · | · | · | · | · | · | · | · |
| **H 04** | · | · | · | · | · | · | · | · | · | · | · | · | · |
| **H 05** | · | · | · | · | · | · | · | · | · | · | · | · | · |
| **H 06** | · | · | · | · | · | · | · | · | · | · | · | · | · |
| **H 07** | · | · | · | · | · | · | · | · | · | · | · | · | · |
| **H 08** | · | · | · | · | · | · | · | · | · | · | · | · | · |
| **H 09** | · | · | · | · | · | · | · | · | · | · | · | · | · |
| **H 10** | · | · | · | · | · | · | · | · | · | · | · | · | · |
| **H 11** | · | · | · | · | · | · | · | · | · | · | · | · | · |
| **H 12** | · | · | · | · | · | · | · | · | · | · | · | · | · |
| **H 13** | · | · | · | · | · | · | · | · | · | · | · | · | · |
| **H 14** | · | · | · | · | · | · | · | · | · | · | · | · | · |
| **H 15** | · | · | · | · | · | · | · | · | · | · | · | · | · |
| **H 16** | · | · | · | · | · | · | · | · | · | · | · | · | · |
| **H 17** | · | · | · | · | · | · | · | · | · | · | · | · | · |
| **H 18** | · | · | · | · | · | · | · | · | · | · | · | · | · |
| **H 19** | · | G | · | · | · | · | · | · | · | · | · | · | · |
| **H 20** | · | · | · | · | · | · | · | · | · | · | · | · | · |
| **H 21** | · | · | · | · | · | · | · | · | · | · | · | · | · |
| **H 22** | · | · | · | · | · | · | · | · | · | · | · | · | · |
| **H 23** | · | · | · | · | · | · | · | · | · | · | · | · | · |
| **H 24** | A | · | · | · | · | · | · | · | · | · | · | · | · |
| **H 25** | · | · | · | · | · | · | · | · | · | · | · | · | · |

**Supplementary Table 4.** Continued.

| **NL** | **1467** | **1472** | **1484** | **1485** | **1490** | **1503** | **1534** | **1535** | **1537** | **1539** | **1541** | **1555** | **1558** |
| --- | --- | --- | --- | --- | --- | --- | --- | --- | --- | --- | --- | --- | --- |
| **H 26** | A | · | · | · | · | · | · | · | · | · | · | · | · |
| **H 27** | · | · | · | · | · | · | · | · | · | · | · | · | · |
| **H 28** | · | · | · | · | · | · | · | · | · | · | · | · | · |
| **H 29** | · | · | · | C | · | · | · | · | · | · | · | · | · |
| **H 30** | · | · | · | · | · | · | · | · | · | · | · | · | · |
| **H 31** | · | · | · | · | · | · | · | · | · | · | · | · | · |
| **H 32** | · | · | · | · | · | · | · | C | · | · | · | · | · |
| **H 33** | · | · | · | · | · | · | · | · | · | · | · | · | · |
| **H 34** | · | · | · | · | · | · | · | · | · | · | · | · | · |
| **H 35** | · | · | · | · | · | · | · | · | · | · | · | · | · |
| **H 36** | · | · | · | · | · | · | · | · | · | · | · | · | · |
| **H 37** | · | · | · | · | · | · | · | · | · | · | · | · | · |
| **H 38** | · | · | · | · | · | · | · | · | · | · | · | · | · |
| **H 39** | · | · | · | · | · | · | · | · | · | · | · | · | · |
| **H 40** | · | · | · | · | · | · | · | · | · | · | · | · | · |
| **H 41** | · | · | · | · | · | · | · | · | · | · | · | · | · |
| **H 42** | · | · | · | · | · | A | · | · | · | · | · | · | · |
| **H 43** | · | · | · | · | · | · | · | · | · | · | · | · | · |
| **H 44** | · | · | · | · | · | · | · | · | · | · | · | · | · |
| **H 45** | · | · | · | · | · | · | · | · | · | · | · | · | · |
| **H 46** | · | · | · | · | · | · | · | · | · | · | · | · | · |
| **H 47** | · | · | · | · | · | · | · | · | · | · | · | · | · |
| **H 48** | · | · | · | · | · | · | · | · | · | · | · | · | · |
| **H 49** | · | · | · | · | · | · | A | · | · | · | · | · | · |
| **H 50** | · | · | · | · | · | · | · | · | · | · | · | · | T |

**Supplementary Table 4.** Continued.

| **NL** | **1467** | **1472** | **1484** | **1485** | **1490** | **1503** | **1534** | **1535** | **1537** | **1539** | **1541** | **1555** | **1558** |
| --- | --- | --- | --- | --- | --- | --- | --- | --- | --- | --- | --- | --- | --- |
| **H 51** | · | · | · | · | · | · | · | · | · | · | · | · | · |
| **H 52** | · | · | · | · | · | · | · | · | · | · | · | · | · |
| **H 53** | · | · | · | · | · | · | · | · | · | · | · | · | · |
| **H 54** | · | · | · | · | · | · | · | · | · | · | · | · | · |
| **H 55** | · | · | · | · | · | · | · | · | · | · | · | · | · |
| **H 56** | A | · | · | · | · | · | · | · | · | · | · | · | · |
| **H 57** | · | · | · | · | · | · | · | · | · | · | · | · | · |
| **H 58** | · | · | · | · | · | · | · | · | · | · | · | · | · |
| **H 59** | · | · | · | · | · | · | · | · | · | · | · | · | · |
| **H 60** | · | · | · | · | · | · | · | · | · | · | · | · | · |
| **H 61** | · | · | · | · | · | · | · | · | · | · | · | · | · |
| **H 62** | · | · | · | · | · | · | · | · | · | · | · | · | · |
| **H 63** | · | · | · | · | · | · | · | · | · | · | · | · | · |
| **H 64** | · | · | · | · | T | · | · | · | · | · | · | · | · |
| **H 65** | · | · | G | · | · | · | · | · | · | · | · | · | · |
| **H 66** | · | · | · | · | · | · | · | · | · | · | · | · | · |
| **H 67** | · | · | · | · | · | · | · | · | · | · | · | · | · |
| **H 68** | · | · | · | · | · | · | · | · | · | · | · | · | · |
| **H 69** | · | · | · | · | · | · | · | · | · | · | · | · | · |
| **H 70** | · | · | · | · | · | · | · | · | · | · | · | · | · |
| **H 71** | · | · | · | · | · | · | · | · | · | · | · | · | · |
| **H 72** | · | · | · | · | · | · | · | · | · | · | · | · | · |
| **H 73** | · | · | · | · | · | · | · | · | · | · | · | · | · |
| **H 74** | · | · | · | · | · | · | · | · | · | · | · | · | · |
| **H 75** | · | · | · | · | T | · | · | · | · | · | · | · | · |

**Supplementary Table 4.** Continued.

| **NL** | **1467** | **1472** | **1484** | **1485** | **1490** | **1503** | **1534** | **1535** | **1537** | **1539** | **1541** | **1555** | **1558** |
| --- | --- | --- | --- | --- | --- | --- | --- | --- | --- | --- | --- | --- | --- |
| **H 76** | · | · | · | · | · | · | · | · | · | · | · | · | · |
| **H 77** | · | · | · | · | · | · | · | · | · | · | · | · | · |
| **H 78** | · | · | · | · | · | · | · | · | · | · | · | · | · |
| **H 79** | · | · | · | · | · | · | · | · | A | · | · | · | · |
| **H 80** | · | · | · | · | · | · | · | · | · | A | · | · | · |
| **H 81** | · | · | · | · | · | · | · | · | · | · | · | · | · |
| **H 82** | · | · | · | · | · | · | · | · | · | · | · | · | · |
| **H 83** | · | · | · | · | · | · | · | · | · | · | · | · | · |
| **H 84** | · | · | · | · | · | · | · | · | · | · | · | · | · |
| **H 85** | · | · | · | · | · | · | · | · | · | · | · | · | · |
| **H 86** | · | · | · | · | · | · | · | · | · | · | · | · | · |
| **H 87** | · | · | · | · | · | · | · | · | · | · | · | · | · |
| **H 88** | · | · | · | · | · | · | · | · | · | · | · | · | · |
| **H 89** | · | · | · | · | · | · | · | · | · | · | · | · | · |
| **H 90** | · | · | · | · | · | · | · | · | · | · | · | · | · |
| **H 91** | · | · | · | · | · | · | · | · | · | · | · | · | · |
| **H 92** | · | · | · | · | · | · | · | · | · | · | · | · | · |
| **H 93** | · | · | · | · | · | · | · | · | · | · | · | · | · |
| **H 94** | · | · | · | · | · | · | · | · | · | · | · | · | · |
| **H 95** | · | · | · | · | · | · | · | · | · | · | · | · | · |
| **H 96** | · | · | · | · | · | · | · | · | · | · | · | T | · |
| **H 97** | · | · | · | · | · | · | · | · | · | · | G | · | · |
| **H 98** | · | · | · | · | · | · | · | · | · | · | · | · | · |

NL, Nucleotide loci
