# Supplementary material for: Genetic structure and demographic history of Lymantria dispar (Linnaeus, 1758) (Lepidoptera: Erebidae) in its area of origin and adjacent areas
Source: Ecol Evol. 2017 Sep 30;7(21):9162–78. doi: 10.1002/ece3.3467 (PMC5677484; doi:10.1002/ece3.3467)
Supplement: Supplementary file 5 [file ECE3-7-9162-s005.docx]

**Supplementary Table 5**

| LP | H01 | H02 | H03 | H04 | H05 | H06 | H07 | H08 | H09 | H10 | H11 | H12 | H13 | H14 | H15 | H16 | H17 | H18 | H19 | H20 | H21 | H22 |
| --- | --- | --- | --- | --- | --- | --- | --- | --- | --- | --- | --- | --- | --- | --- | --- | --- | --- | --- | --- | --- | --- | --- |
| Site 01 | 18 | 1 | 1 | 1 | 1 | 2 | 1 | 1 | 2 | 1 | 1 |  |  |  |  |  |  |  |  |  |  |  |
| Site 06 | 14 |  | 2 |  |  | 3 |  |  | 1 |  |  | 1 | 1 | 1 | 1 | 1 | 1 | 1 | 1 |  |  |  |
| Site 10 | 11 |  | 3 |  |  |  |  |  | 1 | 1 |  |  |  |  | 1 |  |  |  |  | 1 | 1 | 2 |
| Site 12 | 6 | 1 | 1 | 1 |  |  |  |  | 1 |  |  |  |  |  |  |  |  |  |  |  |  |  |
| Site 16 | 9 |  | 2 |  |  |  |  |  |  |  |  |  |  |  |  |  |  |  |  | 1 |  |  |
| Site 18 | 8 | 1 | 1 |  |  |  |  |  |  |  |  |  |  |  |  |  |  |  |  | 2 |  |  |
| Site 22 | 6 |  | 1 |  |  |  |  |  | 1 |  |  |  |  |  |  |  |  |  |  | 2 |  |  |
| Site 26 | 7 |  | 1 |  |  |  |  |  |  |  |  |  |  |  |  |  |  |  |  | 1 |  |  |
| Site 27 | 1 |  | 2 |  |  |  |  |  |  |  |  |  |  |  |  |  |  |  |  |  |  |  |
| Site 28 | 8 |  |  |  |  |  |  |  |  |  |  |  |  |  |  |  |  |  |  |  |  |  |
| Site 30 | 5 |  |  |  |  |  |  |  |  |  |  |  |  |  |  |  |  |  |  | 2 |  |  |
| Site 31 | 17 |  |  |  |  |  |  |  | 2 |  |  |  |  |  |  |  |  |  |  |  |  |  |
| Site 33 | 13 | 8 | 1 |  |  |  |  |  | 2 |  |  |  |  |  | 1 |  |  |  |  |  |  |  |
| Site 34 | 12 |  |  |  |  |  |  |  | 1 |  |  |  |  |  |  |  |  |  |  |  |  |  |
| Site 35 |  |  |  |  |  |  |  |  |  |  |  |  |  |  |  |  |  |  |  |  |  |  |
| Site 36 |  |  |  |  |  |  |  |  |  |  |  |  |  |  |  |  |  |  |  |  |  |  |
| Site 37 |  |  |  |  |  |  |  |  |  |  |  |  |  |  |  |  |  |  |  |  |  |  |
| Site 38 | 1 |  |  |  |  |  |  |  |  |  |  |  |  |  |  |  |  |  |  |  |  |  |
| Site 39 | 13 | 1 | 1 |  |  |  |  |  |  |  |  |  |  |  |  |  |  |  |  |  |  |  |
| Site 41 | 2 |  |  |  |  |  |  |  |  |  |  |  |  |  |  |  |  |  |  |  |  |  |
| Total | 151 | 12 | 16 | 2 | 1 | 5 | 1 | 1 | 11 | 2 | 1 | 1 | 1 | 1 | 3 | 1 | 1 | 1 | 1 | 9 | 1 | 2 |

**Supplementary Table 5.** Continued.

| LP | H23 | H24 | H25 | H26 | H27 | H28 | H29 | H30 | H31 | H32 | H33 | H34 | H35 | H36 | H37 | H38 | H39 | H40 | H41 | H42 | H43 | H44 |
| --- | --- | --- | --- | --- | --- | --- | --- | --- | --- | --- | --- | --- | --- | --- | --- | --- | --- | --- | --- | --- | --- | --- |
| Site 01 |  |  |  |  |  |  |  |  |  |  |  |  |  |  |  |  |  |  |  |  |  |  |
| Site 06 |  |  |  |  |  |  |  |  |  |  |  |  |  |  |  |  |  |  |  |  |  |  |
| Site 10 | 1 | 1 | 1 | 1 | 1 |  |  |  |  |  |  |  |  |  |  |  |  |  |  |  |  |  |
| Site 12 |  |  |  |  |  | 1 | 1 | 1 | 1 | 1 | 1 | 1 | 1 | 1 | 1 | 1 | 1 | 1 |  |  |  |  |
| Site 16 |  |  |  |  |  |  |  |  |  |  |  |  |  |  |  |  |  | 1 | 1 | 1 | 1 | 1 |
| Site 18 |  |  |  |  |  |  |  |  |  |  |  |  |  |  | 9 |  |  |  |  |  |  |  |
| Site 22 |  |  |  |  |  |  |  |  |  |  |  |  |  |  | 11 |  |  |  |  |  |  |  |
| Site 26 |  |  |  |  |  |  |  |  |  |  |  |  |  |  | 11 | 1 |  |  |  |  |  |  |
| Site 27 |  |  |  |  |  |  |  |  |  |  |  |  |  |  | 12 |  |  | 1 |  |  |  |  |
| Site 28 |  |  |  |  |  |  |  |  |  |  |  |  | 1 |  | 11 |  |  |  |  |  |  |  |
| Site 30 |  |  |  |  |  |  |  |  |  |  |  |  |  |  | 16 |  |  |  |  |  |  |  |
| Site 31 |  |  | 1 |  |  |  |  |  |  |  |  |  |  |  | 4 |  |  |  |  |  |  |  |
| Site 33 |  |  |  |  |  |  |  |  |  |  |  |  |  |  |  |  |  |  |  |  |  |  |
| Site 34 |  |  |  |  |  |  |  |  |  |  |  |  |  |  |  |  |  |  |  |  |  |  |
| Site 35 |  |  |  |  |  |  |  |  |  |  |  |  |  |  |  |  |  |  |  |  |  |  |
| Site 36 |  |  |  |  |  |  |  |  |  |  |  |  |  |  |  |  |  |  |  |  |  |  |
| Site 37 |  |  |  |  |  |  |  |  |  |  |  |  |  |  |  |  |  |  |  |  |  |  |
| Site 38 |  |  |  |  |  |  |  |  |  |  |  |  |  |  |  |  |  |  |  |  |  |  |
| Site 39 |  |  |  |  |  |  |  |  |  |  |  |  |  |  |  |  |  |  |  |  |  |  |
| Site 41 |  |  |  |  | 1 |  |  |  |  |  |  |  |  |  |  |  |  |  |  |  |  |  |
| Total | 1 | 1 | 2 | 1 | 2 | 1 | 1 | 1 | 1 | 1 | 1 | 1 | 2 | 1 | 75 | 2 | 1 | 3 | 1 | 1 | 1 | 1 |

**Supplementary Table 5.** Continued.

| LP | H45 | H46 | H47 | H48 | H49 | H50 | H51 | H52 | H53 | H54 | H55 | H56 | H57 | H58 | H59 | H60 | H61 | H62 | H63 | H64 | H65 | H66 |
| --- | --- | --- | --- | --- | --- | --- | --- | --- | --- | --- | --- | --- | --- | --- | --- | --- | --- | --- | --- | --- | --- | --- |
| Site 01 |  |  |  |  |  |  |  |  |  |  |  |  |  |  |  |  |  |  |  |  |  |  |
| Site 06 |  |  |  |  |  |  |  |  |  |  |  |  |  |  |  |  |  |  |  |  |  |  |
| Site 10 |  |  |  |  |  |  |  |  |  |  |  |  |  |  |  |  |  |  |  |  |  |  |
| Site 12 |  |  |  |  |  |  |  |  |  |  |  |  |  |  |  |  |  |  |  |  |  |  |
| Site 16 |  |  |  |  |  |  |  |  |  |  |  |  |  |  |  |  |  |  |  |  |  |  |
| Site 18 | 1 | 1 | 1 | 2 | 1 | 1 |  |  |  |  |  |  |  |  |  |  |  |  |  |  |  |  |
| Site 22 |  |  |  |  |  |  | 1 | 1 | 3 | 1 | 1 | 1 |  |  |  |  |  |  |  |  |  |  |
| Site 26 |  |  |  |  |  |  |  |  | 1 |  |  |  | 1 | 1 | 1 | 1 | 1 | 1 | 1 | 1 |  |  |
| Site 27 |  |  |  |  |  |  |  |  |  |  |  |  |  |  |  | 1 |  |  |  |  | 1 | 1 |
| Site 28 |  |  |  |  |  |  |  |  |  |  |  |  |  |  |  |  |  |  |  |  |  |  |
| Site 30 | 1 |  |  |  |  |  |  |  |  |  |  |  |  |  |  |  |  |  |  |  |  |  |
| Site 31 |  |  |  |  |  |  |  |  |  |  |  |  |  |  |  |  |  |  |  |  |  |  |
| Site 33 |  |  |  |  |  |  |  |  |  |  |  |  |  |  |  |  |  |  |  |  |  |  |
| Site 34 |  |  |  |  |  |  |  |  |  |  |  |  |  |  |  |  |  |  |  |  |  |  |
| Site 35 |  |  |  |  |  |  |  |  |  |  |  |  |  |  |  |  |  |  |  |  |  |  |
| Site 36 |  |  |  |  |  |  |  |  |  |  |  |  |  |  |  |  |  |  |  |  |  |  |
| Site 37 |  |  |  |  |  |  |  |  |  |  |  |  |  |  |  |  |  |  |  |  |  |  |
| Site 38 |  |  |  |  |  |  |  |  |  |  |  |  |  |  |  |  |  |  |  |  |  |  |
| Site 39 |  |  |  |  |  |  |  |  |  |  |  |  |  |  |  |  |  |  |  |  |  |  |
| Site 41 |  |  |  |  |  |  |  |  |  |  |  |  |  |  |  |  |  |  |  |  |  |  |
| Total | 2 | 1 | 1 | 2 | 1 | 1 | 1 | 1 | 4 | 1 | 1 | 1 | 1 | 1 | 1 | 2 | 1 | 1 | 1 | 1 | 1 | 1 |

**Supplementary Table 5.** Continued.

| LP | H67 | H68 | H69 | H70 | H71 | H72 | H73 | H74 | H75 | H76 | H77 | H78 | H79 | H80 | H81 | H82 | H83 | H84 | H85 | H86 | H87 | H88 |
| --- | --- | --- | --- | --- | --- | --- | --- | --- | --- | --- | --- | --- | --- | --- | --- | --- | --- | --- | --- | --- | --- | --- |
| Site 01 |  |  |  |  |  |  |  |  |  |  |  |  |  |  |  |  |  |  |  |  |  |  |
| Site 06 |  |  |  |  |  |  |  |  |  |  |  |  |  |  |  |  |  |  |  |  |  |  |
| Site 10 |  |  |  |  |  |  |  |  |  |  |  |  |  |  |  |  |  |  |  |  |  |  |
| Site 12 |  |  |  |  |  |  |  |  |  |  |  |  |  |  |  |  |  |  |  |  |  |  |
| Site 16 |  |  |  |  |  |  |  |  |  |  |  |  |  |  |  |  |  |  |  |  |  |  |
| Site 18 |  |  |  |  |  |  |  |  |  |  |  |  |  |  |  |  |  |  |  |  |  |  |
| Site 22 |  |  |  |  |  |  |  |  |  |  |  |  |  |  |  |  |  |  |  |  |  |  |
| Site 26 |  |  |  |  |  |  |  |  |  |  |  |  |  |  |  |  |  |  |  |  |  |  |
| Site 27 | 1 | 1 | 1 |  |  |  |  |  |  |  |  |  |  |  |  |  |  |  |  |  |  |  |
| Site 28 |  |  |  | 2 | 1 | 1 | 2 | 1 | 1 |  |  |  |  |  |  |  |  |  |  |  |  |  |
| Site 30 |  |  |  |  |  |  |  |  |  | 2 | 1 | 1 | 1 | 1 |  |  |  |  |  |  |  |  |
| Site 31 |  |  |  |  |  |  |  |  |  |  |  |  |  |  | 2 | 1 | 1 | 1 | 1 |  |  |  |
| Site 33 |  |  |  |  |  |  |  |  |  |  |  |  |  |  |  |  |  |  |  | 1 | 1 | 1 |
| Site 34 |  |  |  |  |  |  |  |  |  |  |  |  |  |  |  |  |  |  |  |  |  |  |
| Site 35 |  |  |  |  |  |  |  |  |  |  |  |  |  |  |  |  |  |  |  |  |  |  |
| Site 36 |  |  |  |  |  |  |  |  |  |  |  |  |  |  |  |  |  |  |  |  |  |  |
| Site 37 |  |  |  |  |  |  |  |  |  |  |  |  |  |  |  |  |  |  |  |  |  |  |
| Site 38 |  |  |  |  |  |  |  |  |  |  |  |  |  |  |  |  |  |  |  |  |  |  |
| Site 39 |  |  |  |  |  |  |  |  |  |  |  |  |  |  |  |  |  |  |  |  |  |  |
| Site 41 |  |  |  |  |  |  |  |  |  |  |  |  |  |  |  | 1 |  |  |  |  |  |  |
| Total | 1 | 1 | 1 | 2 | 1 | 1 | 2 | 1 | 1 | 2 | 1 | 1 | 1 | 1 | 2 | 2 | 1 | 1 | 1 | 1 | 1 | 1 |

**Supplementary Table 5.** Continued.

| LP | H89 | H90 | H91 | H92 | H93 | H94 | H95 | H96 | H97 | H98 | Sn | GD | ND |
| --- | --- | --- | --- | --- | --- | --- | --- | --- | --- | --- | --- | --- | --- |
| Site 01 |  |  |  |  |  |  |  |  |  |  | 30 | 0.6437±0.1004 | 0.012792±0.009624 |
| Site 06 |  |  |  |  |  |  |  |  |  |  | 28 | 0.7487±0.0858 | 0.015593±0.011132 |
| Site 10 |  |  |  |  |  |  |  |  |  |  | 26 | 0.8185±0.0733 | 0.020561±0.013747 |
| Site 12 |  |  |  |  |  |  |  |  |  |  | 23 | 0.9407±0.0432 | 0.027156±0.017203 |
| Site 16 |  |  |  |  |  |  |  |  |  |  | 17 | 0.7279±0.1143 | 0.010900±0.008836 |
| Site 18 |  |  |  |  |  |  |  |  |  |  | 28 | 0.8254±0.0512 | 0.016060±0.011377 |
| Site 22 |  |  |  |  |  |  |  |  |  |  | 29 | 0.8177±0.0564 | 0.021617±0.014229 |
| Site 26 |  |  |  |  |  |  |  |  |  |  | 30 | 0.8253±0.0561 | 0.018418±0.012572 |
| Site 27 |  |  |  |  |  |  |  |  |  |  | 22 | 0.7100±0.1064 | 0.016552±0.011754 |
| Site 28 |  |  |  |  |  |  |  |  |  |  | 28 | 0.7751±0.0584 | 0.016682±0.011702 |
| Site 30 |  |  |  |  |  |  |  |  |  |  | 30 | 0.6966±0.0836 | 0.015659±0.011139 |
| Site 31 |  |  |  |  |  |  |  |  |  |  | 30 | 0.6690±0.0910 | 0.011062±0.008691 |
| Site 33 |  |  |  |  |  |  |  |  |  |  | 28 | 0.7169±0.0672 | 0.013477±0.010014 |
| Site 34 |  |  |  |  |  |  |  |  |  |  | 13 | 0.1538±0.1261 | 0.001810±0.003024 |
| Site 35 | 1 | 15 | 2 | 3 | 2 |  |  |  |  |  | 23 | 0.5652±0.1123 | 0.010695±0.008581 |
| Site 36 |  | 18 | 1 | 1 | 1 | 1 | 1 |  |  |  | 23 | 0.3953±0.1279 | 0.006045±0.005896 |
| Site 37 |  | 19 | 1 |  | 2 |  | 1 |  |  |  | 23 | 0.3202±0.1211 | 0.004929±0.005197 |
| Site 38 |  | 21 | 2 |  | 2 |  |  |  |  |  | 26 | 0.3477±0.1149 | 0.006878±0.006366 |
| Site 39 |  |  |  |  |  |  |  | 1 | 1 |  | 17 | 0.4265±0.1468 | 0.005536±0.005671 |
| Site 41 |  |  |  |  | 1 |  |  |  |  | 1 | 6 | 0.9333±0.1217 | 0.023529±0.017712 |
| Total | 1 | 73 | 6 | 4 | 8 | 1 | 2 | 1 | 1 | 1 | 480 | 0.6529±0.0929 | 0.013798±0.010223 |

LP, Local population; Sn, Sample number; GD, Gene diversity; ND, Nucleotide diversity.
